# Widespread Decreases in Cerebral Copper Are Common to Parkinson's Disease Dementia and Alzheimer's Disease Dementia

**DOI:** 10.3389/fnagi.2021.641222

**Published:** 2021-03-03

**Authors:** Melissa Scholefield, Stephanie J. Church, Jingshu Xu, Stefano Patassini, Federico Roncaroli, Nigel M. Hooper, Richard D. Unwin, Garth J. S. Cooper

**Affiliations:** ^1^Division of Cardiovascular Sciences, Faculty of Biology, Medicine and Health, Centre for Advanced Discovery & Experimental Therapeutics, School of Medical Sciences, The University of Manchester, Manchester Academic Health Science Centre, Manchester, United Kingdom; ^2^Faculty of Science, School of Biological Sciences, University of Auckland, Auckland, New Zealand; ^3^Geoffrey Jefferson Brain Research Centre, Manchester Academic Health Science Centre, Manchester, United Kingdom; ^4^Division of Neuroscience and Experimental Psychology, Faculty of Brain and Mental Health, School of Biological Sciences, University of Manchester, Manchester, United Kingdom; ^5^Division of Neuroscience & Experimental Psychology, Faculty of Biology, Medicine and Health, Manchester Academic Health Science Centre, School of Biological Sciences, The University of Manchester, Manchester, United Kingdom; ^6^Stoller Biomarker Discovery Centre & Division of Cancer Sciences, Faculty of Biology, Medicine and Health, School of Medical Sciences, The University of Manchester, Manchester, United Kingdom

**Keywords:** Parkinson's disease dementia, Alzheimer's disease, essential metals, copper, metallomics study, mass spectrometry, inductively coupled plasma mass spectrometry, human brain study

## Abstract

Several studies of Parkinson's disease (PD) have reported dysregulation of cerebral metals, particularly decreases in copper and increases in iron in substantia nigra (SN). However, few studies have investigated regions outside the SN, fewer have measured levels of multiple metals across different regions within the same brains, and there are no currently-available reports of metal levels in Parkinson's disease dementia (PDD). This study aimed to compare concentrations of nine essential metals across nine different brain regions in cases of PDD and controls. Investigated were: primary motor cortex (MCX); cingulate gyrus (CG); primary visual cortex (PVC); hippocampus (HP); cerebellar cortex (CB); SN; locus coeruleus (LC); medulla oblongata (MED); and middle temporal gyrus (MTG), thus covering regions with severe, moderate, or low levels of neuronal loss in PDD. Levels of eight essential metals and selenium were determined using an analytical methodology involving the use of inductively-coupled plasma mass spectrometry (ICP-MS), and compared between cases and controls, to better understand the extent and severity of metal perturbations. Findings were also compared with those from our previous study of sporadic Alzheimer's disease dementia (ADD), which employed equivalent methods, to identify differences and similarities between these conditions. Widespread copper decreases occurred in PDD in seven of nine regions (exceptions being LC and CB). Four PDD-affected regions showed similar decreases in ADD: CG, HP, MTG, and MCX. Decreases in potassium and manganese were present in HP, MTG and MCX; decreased manganese was also found in SN and MED. Decreased selenium and magnesium were present in MCX, and decreased zinc in HP. There was no evidence for increased iron in SN or any other region. These results identify alterations in levels of several metals across multiple regions of PDD brain, the commonest being widespread decreases in copper that closely resemble those in ADD, pointing to similar disease mechanisms in both dementias.

## Introduction

Parkinson's disease (PD) is one of the most common neurodegenerative disorders, affecting around 1% of the world's population above the age of 60 (Tysnes and Storstein, 2017). Clinically, PD is characterized by progressive motor dysfunction resulting in bradykinesia, tremor, and rigidity. Cognitive impairment is also common, with cohort studies reporting around a third of patients as showing mild cognitive impairment (MCI) at the time of diagnosis, and up to 80% developing dementia within 20 years after onset of symptoms (Hanagasi et al., 2017). When dementia has developed, the condition is described as PD with dementia (also known as Parkinson's disease dementia, and Parkinsonian dementia) and abbreviated as PDD.

PD and PDD are characterized by the degeneration of dopaminergic neurons in the substantia nigra and the accumulation of misfolded α-synuclein in Lewy bodies and neuropil threads (Braak et al., 2003). However, research is ongoing on exactly how these inclusions develop or how they lead to neuronal death. Although clinical trials targeting the aggregation and binding of α-synuclein, and/or aiming to changes its levels are ongoing, to date no drugs modifying misfolded α-synuclein have been developed (Oliveri, 2019).

Therefore, research in PD is beginning to focus on other mechanisms that may contribute to pathogenesis, including the roles of mitochondrial dysfunction and oxidative stress (Rocha et al., 2018), autophagy (Cerri and Blandini, 2019), genetic factors (Raza et al., 2019), lipid dysfunction (Xicoy et al., 2019), inflammation (Rocha et al., 2018), and metal homeostasis (Ward et al., 2014; Bjorklund et al., 2018). Perturbations of metals have been reported in PD brain tissues. Increases in iron (Fe) have been consistently reported in the substantia nigra (SN), the region most severely affected in PD (Dexter et al., 1989; Ayton et al., 2013; Kim and Lee, 2014; Pyatigorskaya et al., 2015; Costa-Mallen et al., 2017; Genoud et al., 2017; Xuan et al., 2017). Likewise, there have been several reports of reduced copper (Cu) within the SN (Ayton et al., 2013; Davies et al., 2014; Genoud et al., 2017). Some studies examined other brain regions, usually observing no changes in Cu outside the SN (Riederer et al., 1989; Ayton et al., 2013; Genoud et al., 2017), but with one study showing decreases in the caudate nucleus (CN) and locus coeruleus (LC) (Davies et al., 2014). Additionally, most investigations did not distinguish between PD and PDD and primarily focused on the former; indeed, there are no available studies known to us reporting brain metal levels in PDD itself. It therefore remained unknown whether different or additional metal alterations characterize PDD.

To answer this question, we investigated levels of eight essential metals including sodium (Na), magnesium (Mg), potassium (K), calcium (Ca), manganese (Mn), Fe, Cu, zinc (Zn), and selenium (Se), across nine brain regions including the cerebellum at the level of the dentate nucleus (CB), cingulate gyrus (CG), hippocampus (HP), LC, motor cortex (MCX), medulla (MED), middle temporal gyrus (MTG), occipital cortex at the level of the primary visual cortex (PVC), and SN in nine confirmed cases of PDD and nine age-matched control brains. Essential metals were chosen for investigation due to the physiological necessity of tightly-controlled levels for health and survival in humans, as well as on the basis of previous reports of alterations of such elements not only in PD, but also across multiple brain regions in other neurodegenerative conditions such as Alzheimer's (AD) (Deibel et al., 1996; Loeffler et al., 1996; Xu et al., 2017) and Huntington's disease (HD) (Bartzokis et al., 2007; Reetz et al., 2012; Rosas et al., 2012). The regions selected here were chosen to cover areas highly affected by neurodegeneration in PD/PDD (SN, MED, LC), as well as moderately-affected regions (MCX, HP, PVC, CG) and relatively-spared regions (CB, MTG). In addition to allowing direct comparison of areas differently affected by neurodegeneration, this selection was also expected to allow comparison of results with those of previous parallel studies investigating metallomic perturbations in ADD, in order to identify any similarities or differences between the two forms of dementia. Any identified similarities could point toward shared disturbances or pathogenic mechanisms between these diseases, whilst differences could aid in distinguishing between conditions in cases where clinical presentation may make diagnosis and treatment difficult.

## Materials and Methods

### Reagents

Except where otherwise stated, all reagents were obtained from Sigma-Aldrich (UK).

### Acquisition of Human Brain Tissues From PDD Cases and Controls

Brain tissues from nine neuropathologically confirmed PDD cases and nine age-matched controls were obtained from the Multiple Sclerosis & Parkinson's Tissue Bank, Imperial College, London (UK). Complete sets of tissues from the SN, CG, LC, HP, MCX, CB, PVC, MED, MTG were acquired (i.e., nine regions from each of 18 brains in all). These regions were selected on the basis of their varying levels of involvement in PDD and for direct comparison with previous metallomic studies in AD. Post-mortem delay was 48 h or shorter (see Supplementary Material A for individual donor characterization); such post-mortem delays are known not to affect brain metal levels (Scholefield et al., 2020).

### Diagnosis and Severity of Human Cases

The extent of amyloid, α-synuclein, and tau-related pathologies was determined in all cases; these were assessed using CERAD and Thal phase for amyloid, and Braak grading systems for tau and for α-synuclein. In all cases, dementia only developed at least 1 year following the onset of motor symptoms, ruling out a diagnosis of dementia with Lewy bodies (DLB) (McKeith et al., 2017). Co-morbidities, cause of death, smoking status, post-mortem delay, Thal and CERAD scores, presence of signs of small vessel disease, brain weight, age at onset and duration of disease in PDD cases, were recorded. Cases with signs of other dementia conditions, such as Alzheimer's disease, were excluded.

Control brains only showed age-related changes and no evidence of cognitive impairment was reported in the clinical records. Characteristics of individual donors are shown in Supplementary Material A.

### Tissue Dissection

Brain tissue was transferred on dry ice from London to our University of Manchester laboratory and stored at −80°C upon delivery. On the day of the experiment, the specimens were thawed slightly on ice and dissected into 50 mg (±5%) aliquots for ICP-MS using a metal-free ceramic scalpel and placed into “Safe-Lok” microfuge tubes (Eppendorf AG; Hamburg, Germany). Tubes were weighed before adding tissues to allow samples to be dried and weighed without the need for transferring to new tubes. The use of a ceramic scalpel prevented contamination from metals during sectioning. Following dissection, tissues were immediately dried as detailed below.

### ICP-MS

Here we applied a method previously used for measurement of metal levels in brain tissue based on ICP-MS (Xu et al., 2017). Freshly-dissected samples were briefly centrifuged before being dried to a constant weight using a Savant Speedvac (Thermo Fisher Scientific; MA, USA). Digestion of 50 ± 5 mg samples in 2 ml concentrated nitric acid was then performed in a heat block, along with digestion blanks containing nitric acid alone (see Supplementary Material B). Following digestion, sample solutions were refrigerated overnight at 4°C before undergoing ICP-MS analysis with a 7700x ICP-MS spectrometer (Agilent; Santa Clara, CA, USA) equipped with a MicroMist nebulizer and Scott double-pass spray chamber (Glass Expansion; Melbourne, Australia), and nickel sample and skimmer cones. Samples were separated into batches of either one or two regions, with multi-element calibration using calibration standard dilutions and periodic quality controls included for each batch (see Supplementary Material B). Three technical replicates were performed for each batch. All elements were standardized against scandium, with the exception of Zn and Se which were standardized against germanium.

Agilent's manufacturer recommendations were followed for selection of operation mode, integration times, and internal standard assignments. Samples were introduced to the instrument using an integrated autosampler (Agilent). All elements were analyzed using helium as the collision gas; Se was analyzed in high-energy helium mode (10 ml/min helium) due to its state as a polyatomic element (Se naturally exists as a polyatomic ring of eight atoms, giving it a higher ionization energy), and all other elements were analyzed using standard helium mode (5.0 ml/min helium). For each of the quantified elements, limits of quantitation, LOQs; detection limits, DLs; and background equivalent concentrations, BECs; were automatically calculated by the instrument (see Supplementary Material B). Measured metal concentrations from individual runs were disregarded wherever lower than the detection limit calculated for that run, with average concentrations being instead taken from the remaining two replicates.

### Elemental Data Analysis

Mean element values (±95% CI) were calculated and differences between cases and controls determined by non-parametric Mann-Whitney U tests due to the small sample size. Mann-Whitney U calculations were performed using GraphPad v8.1.2 (Prism; La Jolla, CA, USA) and *p*-values < 0.05 were considered significant. Tests of statistical power were carried out due to the small sample sizes to determine the statistical power and minimum sample size required where significant case-control differences were observed (see Supplementary Material D). These tests were carried out using the DSS Statistical Power and Sample Size Calculator (https://www.dssresearch.com/resources/calculators/statistical-power-calculator-average/).

Where possible, values from control cases from previous experiments on AD (samples obtained from the New Zealand National Brain Bank; Auckland, New Zealand; Manchester Brain Bank; Salford Royal Hospital, UK; and the Newcastle Brain Tissue Resource; University of Newcastle, UK) and Huntington's disease (obtained from the New Zealand National Brain Bank) were combined with the current PDD control group values in order to obtain the largest available sample size. Details of individual donors from these cohorts are also included in Supplementary Material A. Suitability of values from previous control groups for inclusion with values from the current PDD-matched control group, was determined by comparison of cohort characteristics as well as element concentrations in each region. This included comparisons of sex, age, and post-mortem delay values between control groups as determined by Mann-Whitney U test, as well as separation analysis by principal component analysis (PCA) and partial least squares discriminant analysis (PLS-DA; see Supplementary Material C). Orthogonal PLS-DA (OPLS-DA) was used where only two control groups were being compared. Extra controls were accepted if they did not differ significantly across control-cohort variables and were not separated from the current control group by PCA or PLS-DA. PCA and PLS-DA analyses were performed using MetaboAnalyst (McGill University; Montreal, Canada). We maximized available control numbers to minimize loss of power and thus make optimal use of all data available to us through our published prior metallomic, metabolomic and proteomic studies of the age-related dementias.

## Results

### Patient Characteristics

Tissues from nine different brain regions were obtained from nine clinically-diagnosed and neuropathologically-confirmed cases of PDD and nine age-matched controls. Where possible, additional control samples derived from previously-described cohorts used in investigations of AD and HD were also included in the analysis (see section “Control Groups”).

No significant differences were found between cases and controls when sex, PMD, tau Braak stage, Thal and CERAD scores, brain weight, and smoker status were compared (see Table 1). PDD cases were about 10 years younger than controls (mean 77.0 vs. 87.6 years, respectively). The cause of death of PDD and control cases is reported in Supplementary Table 2. Smoking status was unknown for three cases (PD5, PD8, and PD9). PDD cases showed a higher incidence of psychiatric symptoms such as anxiety and depression, whereas hypertension and type II diabetes were more common in controls (see Supplementary Table A2). One control had a family history of PD, but no recorded signs and symptoms or neuropathological features alpha-synucleinopathy or any other neurodegenerative disease were observed (C4).

**Table 1:** Cohort characteristics.

**Variable**	**PDD cases (*n* = 9)**	**Controls (*n* = 9)**	***P*-value**
Male sex, *n* (%)	5 (55.5)	4 (44.4)	0.7
Age	78 (66–93)	87 (79–95)^**^	**0.005**
PMD (hours)	27 (9–48)	25 (15–48)	0.8
α-synuclein Braak	6 (5–6)	0^****^	** <0.0001**
tau Braak	2 (0–4)	2 (1–3)	0.9
Thal	2 (0–5)	2.5 (0–1)	0.4
CERAD	0 (0–2)	0 (0–1)	0.7
Disease duration (years)	13 (6–23)	N/A	N/A
Age onset	67 (49–69)	N/A	N/A
Whole brain weight (g)	1371 (960–1402)	1126 (946–1338)	0.0503
Never smoked, *n* (%)	4 (44.4)	2 (22.2)	0.3

*Comparison of PDD case and control variables. All variables are medians (range); p-values for significance of between-group differences were calculated by Mann-Whitney U test*.

** p < 0.01;

**** *p < 0.0001. CERAD, The Consortium to Establish a Registry for Alzheimer's disease score; PMD, post-mortem delay. Significant case-control differences are highlighted in bold*.

#### Control Groups

Our group has previously carried out metallomic analyses of human brains in AD and HD using the methods employed here. These studies included three AD cohorts obtained from the New Zealand National Brain Bank, Manchester Brain Bank, and Newcastle Brain Bank; as well as an HD cohort from the New Zealand National Brain Bank. Where possible, controls from these cohorts have been combined with the controls from the current PDD cohort in order to maximize sample size and improve reliability of the analysis.

The characteristics of each of these control groups are shown in Table 2 (details of individuals from each cohort are provided in Supplementary Material A). Whilst the Newcastle and Manchester-derived AD groups and Auckland-derived HD group did not differ significantly across any of the comparable variables from the PDD controls, the Auckland-derived AD group showed significantly lower age, tau Braak staging, and age than the PDD controls. However, we have previously shown that these variables do not significantly alter metal concentrations in human AD and control brain tissues (Scholefield et al., 2020) and are thus not considered grounds for exclusion.

**Table 2:** Comparison of control cohort characteristics.

**Variable**	**Manchester AD controls**	**Newcastle AD controls**	**Auckland AD controls**	**PDD controls**	**HD controls**
Number	9	9	9	9	9
Age	89 (82–95)	85 (76–94)	73 (61–8)^**^	87 (79–95)	66 (49–81)^**^
Male sex, n (%)	6 (66.6)	6 (66.6)	5 (55.4)	5 (44.4)	6 (66.6)
Tau Braak stage	I-II	I-II	0^****^	II	NA
PMD (hours)	75 (49–130)	25 (9–40)	12 (5.5–15.0)^**^	25 (15–48)	12 (6.5–15)^**^
Whole-brain weight (g)^†^	1160 (1020–1494)	1235 (1064–1406)	1260 (1094–1461)	1126 (946–1338)	1315 (1210–1495)

*Comparison of control group characteristics between each brain bank cohort. Age, male percentage, tau Braak stage, PMD, and whole brain weight are medians (range); p-values for significance of between-group differences were calculated by the Kruskal-Wallis test followed by Dunn's post-hoc multiple comparisons test against the PDD control group*.

** p < 0.01;

**** *p < 0.0001*.

† *Indicates that brain weights were not available for two samples*.

Whether these control groups were suitable for inclusion in this study was assessed on a region-by-region basis. Controls from the Manchester and Newcastle cohorts were available for the CG. Controls from an Auckland-derived AD study were available for the CG, MCX, HP, CB and MTG. Controls from an Auckland-derived HD study were available for the MC, HP, and CB. PCA and PLS-DA plots of available control groups were performed to determine whether element measurements produced separation of different control cohorts (see Supplementary Material C). If separation occurred, the dissimilar control group would not be included; otherwise, all controls were included in the final analysis for that region. On this basis, the Manchester and Newcastle controls were accepted in the CG control group; Auckland AD controls were accepted in the HP control group, but excluded from the CG control group; and the HD controls were accepted in the HP control group but excluded from the SN control group (see Table 3).

**Table 3:** Final control groups for each region.

**Control group**	**MTG**	**CB**	**CG**	**SN**	**HP**	**LC**	**MED**	**PVC**	**MCX**
Manchester AD (*n* = 9)	-	-	Accepted	-	-	-	-	-	-
Newcastle AD (*n* = 9)	-	-	Accepted	-	-	-	-	-	-
Auckland AD (*n* = 9)	Accepted	Excluded	Excluded	-	Accepted	-	-	-	Accepted
Auckland HD (*n* = 9)	Accepted	Excluded	-	Excluded	Accepted	-	-	-	Accepted
Final number	27	9	27	9	27	9	9	9	26

*A dash indicates that the region was not investigated in the indicated cohort. MTG, middle temporal gyrus; CB, cerebellum; CG, cingulate gyrus; SN, substantia nigra; HP, hippocampus; LC, locus coeruleus; MED, medulla; PVC, primary visual cortex (occipital lobe); MCX, motor cortex*.

### Elemental Analysis

The concentrations of Se and eight essential metals including Na, Mg, K, Ca, Mn, Fe, Cu, Zn were determined in dry tissue from nine regions of the brain in nine PDD cases and nine controls without a history or signs of dementia or other neurodegenerative disease. Where possible, measurements from additional controls from previously-analyzed cohorts were also included in the analysis (see Table 3). See Supplementary Material E for individual raw data expressed in both umol/kg-mmol/kg, and ug/g-mg/g.

**Figure 1:**
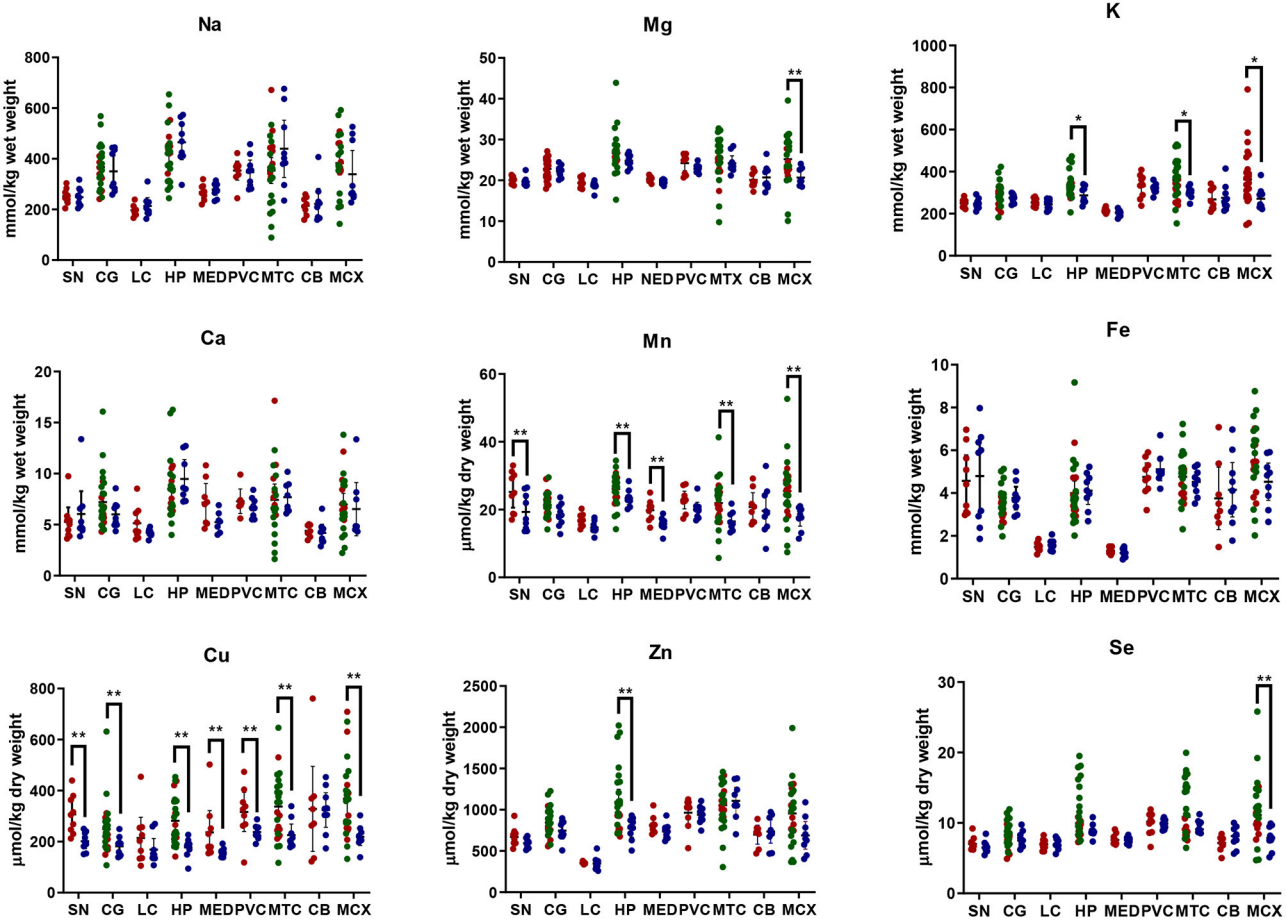
Metal concentrations in PDD cases vs. controls. Case-control differences determined by Mann-Whitney *U*-test. *p < 0.05; **p < 0.01. SN, substantia nigra; CG, cingulate gyrus; LC, locus coeruleus; HP, hippocampus; MED, medulla; PVC, primary visual cortex; MTG, middle temporal gyrus; CB, cerebellum; MCX, motor cortex. Na, sodium; Mg, magnesium; K, potassium; Ca, calcium; Mn, manganese; Fe, iron; Cu, copper; Zn, zinc; Se, selenium.

Element concentrations for each region are shown in Table 4 and Figure 1. Cu concentrations were the most consistently perturbed, being found to be decreased in the CG (*p* = 0.009), SN (*p* = 0.003), HP (*p* = 0.001), MED (*p* = 0.008), PVC (*p* = 0.008), MTG (*p* = 0.005), and MCX (*p* = 0.001) of cases in comparison to controls. Cu levels also trended lower in cases than controls in the remaining two regions, but differences were not significant. Cu concentrations in control regions were largely consistent, averaging around 300 μmol/kg or 18.9 ug/g dry weight, although the MCX had a slightly (but not significantly) higher concentration than other regions (mean of 403.5 μmol/kg or 25.4 μg/g dry weight).

There was evidence that Mn was also decreased in several regions, including the SN (*p* = 0.04), HP (*p* = 0.04), LC (*p* = 0.03), MED (*p* = 0.007), MTG (*p* = 0.006), and MCX (*p* = 0.003). In the remaining regions, there was a non-significant trend toward lower levels in cases compared to controls. Like Cu, Mn concentrations were consistent across control regions, averaging around 20 μmol/kg or 1.1 μg/g dry weight, with the exception of the LC which showed slightly lower values (16.7 μmol/kg or 0.9 μg/g dry weight).

As with Cu and Mn, K levels were lower in cases than controls in every region, but this decrease was only significant in three areas: the HP (*p* = 0.04), MTG (*p* = 0.04), and MCX (*p* = 0.03). *K* levels were lowest in MED controls at 215.8 mmol/kg or 8.4 mg/g dry weight and highest in the MCX of controls at 362.6 mmol/kg or 14.1 mg/g dry weight.

Mg and Se were found to be decreased only in the MCX (*p* = 0.03 and *p* = 0.002, respectively). Control Mg concentrations were also highest in the MCX (26.4 mmol/kg or 0.6 mg/g dry weight) and lowest in the SN (20.0 mmol/kg or 0.5 mg/g dry weight). Se was higher than average in three regions; the MTG (12.6 μmol/kg or 1.0 μg/g dry weight), HP (11.3 μmol/kg or 0.9 μg/g dry weight), and MCX (12.7 μmol/kg or 1.0 μg/g dry weight). All other control regions showed Se levels below 10 μmol/kg or 8 μg/g dry weight.

Zn was only seen be to be lowered in the HP (*p* = 0.009). There was a marked variation in Zn levels across control regions, with a particularly low concentration in the LC (355.4 μmol/kg or 35.7μg/g dry weight) and the highest concentration in the HP itself (1095.5 μmol/kg or 80.6 μg/g dry weight).

No significant differences were found between cases and controls for Na, Ca, or Fe in any region. Na concentrations in control brains were highly variable, with the highest levels observed in the HP (419.4 mmol/kg or 9.6 mg/g dry weight) and the lowest in the LC (196.8 mmol/kg or 4.5 mg/g dry weight). Control Fe concentrations were also highly variable, ranging from 5.8 mmol/kg or 0.3 mg/g dry weight in the MCX to 1.3 mmol/kg or 0.07 mg/g dry weight in the MED, an almost 4-fold difference. Control Ca levels were however fairly similar across regions, with few significant differences between individual areas (data not shown).

**Figure 2:**
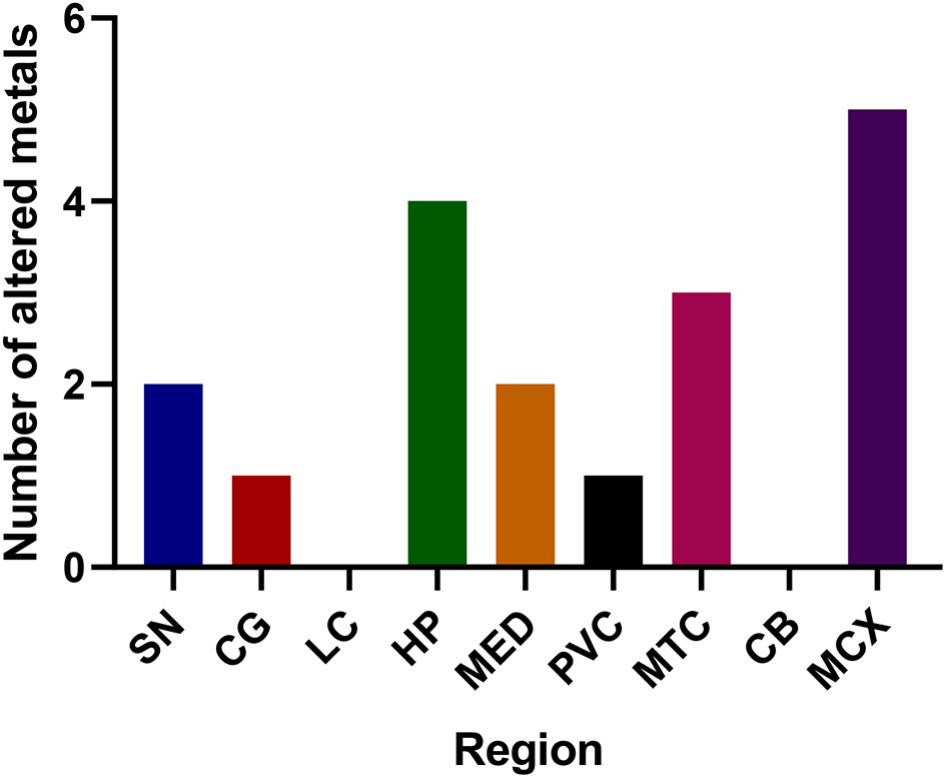
Number of altered metals per brain region. SN, substantia nigra; CG, cingulate gyrus; LC, locus coeruleus; HP, hippocampus; MED, medulla; PVC, primary visual cortex; MTG, middle temporal gyrus; CB, cerebellum; MCX, motor cortex. Na, sodium; Mg, magnesium; K, potassium; Ca, calcium; Mn, manganese; Fe, iron; Cu, copper; Zn, zinc; Se, selenium.

The regions with the highest number of altered elements were the MCX, HP, and MTG (see Figures 1, 2). Interestingly, the SN showed changes in only two metals, Cu and Mn, despite being the region most heavily affected by neurodegeneration in PDD. This indicates a pattern for metal changes that doesn't necessarily reflect α-synuclein deposition or neurodegeneration patterns. Likewise, the LC showed no changes despite having high levels of neuronal loss in PDD. The CB also completely lacked alterations in any of the elements analyzed here. The MED showed changes in two metals, Cu and Mn, like the SN. The CG and PVC both only showed decreases in Cu.

Due to the small sample size used for this study, a retrospective statistical power analysis was carried out to ensure the n number was sufficient to reliably identify significant differences between the case and control groups (see Supplementary Material D). Where significant case-control differences were observed, the tests showed a required sample size ≥18 for significance at *p* > 0.05, with the majority also showing a statistical power of ≥80%, and several showing a statistical power of ≥90%.

### Comparison to AD Findings

We compared our results in PDD with an AD cohort (cases with diagnosed AD dementia at time of death) investigated with the same methodology (Xu et al., 2017) in five regions including the HP, MTG, MCX, CG, and CB. The SN, LC, PVC, and MED were only investigated in PDD, and the entorhinal cortex (ENT) and sensory cortex (SCX) only investigated in AD.

**Figure 3:**
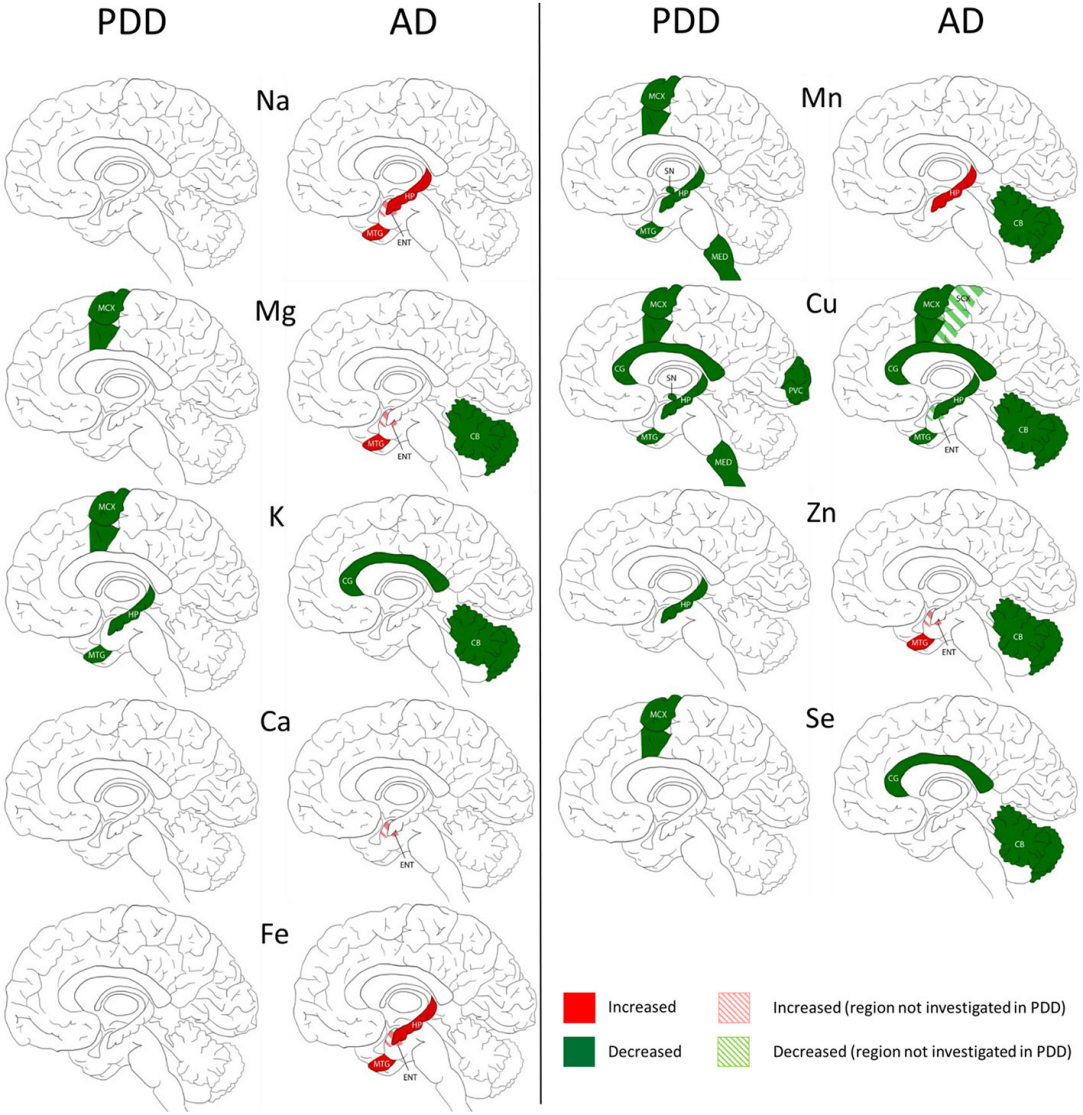
Regional comparison of metal perturbations observed in PDD and AD brains. SN, substantia nigra; CG, cingulate gyrus; LC, locus coeruleus; HP, hippocampus; MED, medulla; PVC, primary visual cortex; MTG, middle temporal gyrus; CB, cerebellum; MCX, motor cortex. Na, sodium; Mg, magnesium; K, potassium; Ca, calcium; Mn, manganese; Fe, iron; Cu, copper; Zn, zinc; Se, selenium.

The widespread decreases in Cu in the MCX, CG, HP, and MTG were the most striking similarity between the PDD and AD (see Figure 3). Cu was also decreased in the SCX, ENT, and CB of AD brains, and in the SN, MED, and PVC of PDD brains. CB was the only region with dissimilar Cu concentration, with decreases in AD and no change in PDD brains. Several perturbations including decreased Mg, K, Mn, Zn, and Se were found in the CB of AD brains but no changes were found at all in the PDD CB, indicating a relative sparing of the CB in PDD compared to AD.

There were no other shared perturbations in metals between AD and PDD. Decreased K was observed in both conditions, however, with regional differences: in the MCX, HP, and MTG of PDD brains, and the CG and CB in AD cases. Likewise, although decreases in Mg, Mn, Zn, and Se were found in both PDD and AD brains, they occurred in differing regions (see Figure 3). Increases in Na, Mg, Ca, Fe, Mn, and Zn were identified in some regions of the AD brain, but not in any region of the PDD brain.

## Discussion

We investigated eight essential metals and Se across nine brain regions in a cohort of clinically and neuropathologically defined PDD cases and documented reduction in Cu levels compared to controls and brains with AD.

Several studies have examined the role of individual metals in the brains with PD or PDD but none have described levels of multiple metals across several brain regions in PDD brains. Some of these studies may use slightly different methodologies in measuring metal levels, for example dry-weight brain tissue was used here whereas some studies may use wet-weight. In this case, an approximate comparison between obtained concentrations can be made by multiplying dry-weight values by five, as the wet brain tissue lost on average 80% of its weight during the drying process (data not shown). Concentrations obtained in this study are reported in both μmol/kg-mmol/kg and μg/g-mg/g in the supplementary material for easier comparison to data from other investigations (see Supplementary Material E). Studies reporting concentrations based on protein content rather than sample weight are more difficult to directly compare to those reported here, but case-control observations should still be comparable.

### Copper (Cu)

That there are widespread decreases in Cu levels across seven of the nine regions investigated represents the most striking observation of this study. Decreased Cu has been reported within the SN (Uitti et al., 1989; Ayton et al., 2013; Davies et al., 2014; Genoud et al., 2017), CN and LC (Davies et al., 2014) of brains with α-synucleinopathy and clinical features of PD but never proved in patients with PD and dementia. Other studies that tested the neocortex (Ayton et al., 2013), occipital cortex (OCC) and frontal gyrus (FG) (Genoud et al., 2017), frontal cortex (FC), CN and CB (Uitti et al., 1989) have reported no change. Levels in the control group were remarkably consistent with previous measures of Cu observed in the MTG, CG, HP, PVC, CB, MED, and midbrain of healthy aged brains (Ramos et al., 2014a). Control levels in the LC were similar to those reported in another study of healthy brains once wet-weight/dry-weight differences were accounted for (Zecca et al., 2004). However, the same study reported much lower Cu levels within the SN compared to the LC, whereas a non-significant trend toward lower Cu was observed in the control LC than the SN in this study. However, this observation in the previous report was based off average Cu levels over an age range of 17–88 years, and did not state if these differences remained in older individuals, such as those used in the current investigation – although it was observed that LC Cu levels decreased with aging, whereas SN Cu showed no change associated with age.

Notably, decreases in Cu levels were observed in several brain regions that we previously found to be affected in AD brains (Xu et al., 2017), including the MCX, CG, HP, and MTG. Comparisons could not be made between the SN, MED, PVC, SCX, or ENT as they were each only investigated in one of the two conditions. One previous study reported no change within the SN in AD (Loeffler et al., 1996) but no data are available to the best of our knowledge on the MED, and PVC in AD, or on the SCX or ENT in PDD.

One region that clearly distinguished PDD from AD in this study was the CB, which has previously shown substantive Cu decreases in AD (Xu et al., 2017), but showed no change here in PDD for Cu or any other metal. It is possible that this dissimilarity might contribute to some of the symptomatic differences between AD and PD, either by cerebellar involvement in the more aggressive cognitive decline seen in AD, or by protection against typical Parkinsonian symptoms that are not often observed in AD such as hallucinations, motor symptoms, or rapid eye movement sleep behavior disorder (Association, 2014). A protective role for the CB is supported by proteomic and metabolomic findings in the AD brain, which have shown that although there are many protein and metabolite changes in the CB in AD, they are drastically different from those observed in regions heavily affected by neuronal loss in AD such as the HP (Xu et al., 2016, 2019). This protective function may be lost in PDD.

Cu is an essential co-factor for several important antioxidants, including superoxide dismutase 1 (SOD1), which is responsible for removing harmful superoxide ions and hydrogen peroxide species from cells. Cu is also an essential component of ceruloplasmin, a ferroxidase which oxidizes reactive ferrous Fe (Fe^2+^) to its non-toxic ferric form (Fe^3+^). This prevents production of hydroxyl radicals by ferrous Fe via the Fenton reaction (Winterbourn, 1995). As such, decreases in Cu may lead to less effective removal and increased production of reactive oxygen species (ROS), resulting in increased oxidative stress in AD and in PDD (Bisaglia and Bubacco, 2020). Indeed, increased oxidative stress has been reported widely in PD cases (Dias et al., 2013). SOD1 itself has been shown to display metal deficiency and to misfold in the SN and LC of PD cases (Trist et al., 2017). Additionally, decreased Cu binding to SOD1 has been proposed to contribute directly to build-up of misfolded and dysfunctional SOD1 in PD brains independently of mutations (Trist et al., 2018).

Cu is also an essential component of cytochrome c oxidase, which is responsible for transferring electrons between subunits III and IV of the mitochondrial electron transport chain (ETC). As such, decreased Cu could impair cytochrome c oxidase function and by extension ATP production via the mitochondrial F_1_F_0_ ATP synthase (also known as the H^+^-ATPase or complex V). Mitochondrial dysfunction in PD is widely recognized (Dias et al., 2013) and experiments in a paraquat-exposed mouse model of PD have reported involvement of cytochrome c oxidase in α-synuclein oligomerisation and radical formation (Kumar et al., 2016).

Together these observations suggest that decreased Cu in PD and AD could result in mitochondrial dysfunction, decreased energy production, increased oxidative stress, and perhaps even increased α-synuclein oligomerisation.

### Manganese (Mn)

Another common finding in this study was decreased Mn, which was observed in five of nine regions investigated here: the MCX, SN, HP, MTG, and MED. Previous studies have reported no change in Mn levels in the SN (Uitti et al., 1989; Genoud et al., 2017), OCC, FG (Genoud et al., 2017), FC, CN or CB (Uitti et al., 1989) of PD patients. No studies investigating any of the other regions covered here could be found. Mn levels in controls were very similar to those observed in the MTG, CG, HP, and PVC of healthy aged brains in a previous investigation, although levels in the MED and CB were slightly higher than the concentrations reported there (Ramos et al., 2014a).

PD has previously been suggested to be linked to increased Mn levels due its clinical similarity to manganism. Manganism is a condition caused by environmental exposure to Mn and is characterized by Parkinsonian symptomology and increased Mn levels in the brain. However, manganism patients do not display neuropathological signs of PD (Perl and Olanow, 2007) or respond to dopaminergic drug treatment (Koller et al., 2004). Mn exposure has been shown to cause dopaminergic neuronal loss (Brouillet et al., 1993) and motor impairments in rats (Cordova et al., 2013). However, surveys on humans show little support for the role of Mn exposure in the risk of developing PD itself, and increased levels have not been reported in the brain (Dusek et al., 2015).

Mn is a cofactor for SOD2. This form of SOD is largely localized to the mitochondria in eukaryotic cells, where it can remove ROS produced by the mitochondrial ETC. As such, decreased Mn may also lead to increased oxidative stress in PDD. Combined with Cu decreases, this could lead to a dramatic decrease in SOD anti-oxidative function. Mn is also required for proper functioning of the urea cycle, with catalyzes the break-down of toxic ammonia-containing compounds to urea for excretion. This requires the enzyme arginase, a Mn-containing enzyme which catalyzes the final step of the urea cycle, converting arginine to ornithine and urea. Decreased Mn could lead to a reduction in arginase activity, resulting in toxic accumulation of ammonia-containing compounds. At present, little to no research has been done investigating a role for the urea cycle in PD or PDD.

### Zinc (Zn)

Zn is the last of the three SOD metal cofactors. Cu/Zn-SOD1 is primarily localized to the cytosol but is also found in the mitochondria (Kawamata and Manfredi, 2008), where it serves an equivalent antioxidative function as Cu-SOD1/3 and Mn-SOD2. This study found decreased Zn in the HP of PDD brains, with no other region affected. Zn has been previously reported to be unchanged in the SN of PD brains (Uitti et al., 1989; Genoud et al., 2017) as well as the OCC and FG (Genoud et al., 2017) and the FC, CN, and CB (Uitti et al., 1989). These reports agree with our own observations in the SN and CB, but no other investigations have yet been performed on the HP. Control levels were consistent with those previously reported in the healthy aged MTG, CG, HP, PVC, and midbrain, although around 50% higher in the MED and 25% higher in the CB (Ramos et al., 2014a). As in the current study, the highest Zn levels were also observed to be in the HP.

In these experiments, the HP of PDD cases was the only region found to have simultaneously decreased Cu, Mn, and Zn, meaning that all three SOD metal cofactors were diminished in this area. Cumulatively, this could have a dramatic effect on SOD function in the PDD HP and resultant oxidative stress. It is possible that this cumulative effect in an area heavily involved in cognitive function contributes to the increase in hippocampal neuronal loss and dysfunction observed in PDD compared to PD without dementia (Hall et al., 2014).

### Selenium (Se)

Se was observed here to be decreased in the MCX only of PDD brains. A single report on Mn in the PD brain reported no change in the FC, CN, SN, or CB (Uitti et al., 1989). There have been no previous investigations of Mn in the PD MCX. Control Se levels were similar to those previously reported in a study on healthy aged brains in the MTG, MED, and CB, but were somewhat lower in the CG, HP, PVC, and particularly within the midbrain, which was around 45% lower than previously reported levels on average (Ramos et al., 2015). These unusually low Se concentrations within the control brains of this cohort may have made it difficult to observe decreases in the PDD cases, particularly as several of these regions showed non-significant trends toward decreases in Se (see Tables 4A–I and Supplementary Material E).

**Table 4A:** Metal concentrations in the SN of PDD cases and controls.

**Metal**	**Units**	**PDD (*n* = 9)**	**Controls (*n* = 9)**	***p*-value**
Na	mmol per kg	248.3 (219.3–277.2)	252.3 (229.5–275.1)	0.7
Mg	mmol per kg	19.7 (18.8–20.6)	20.0 (19.3–20.7)	0.3
K	mmol per kg	246.0 (226.6–265.4)	249.3 (235.0–263.7)	0.6
Ca	mmol per kg	6.0 (3.8–8.3)	5.3 (3.9–6.7)	0.7
Mn	μmol per kg	19.3 (15.2–23.5)	25.2 (20.6–29.9)	**0.04**
Fe	mmol per kg	4.8 (3.1–6.5)	4.6 (3.4–5.8)	1.0
Cu	μmol per kg	201.8 (174.1–229.6)	306.8 (248.4–365.2)	**0.003**
Zn	μmol per kg	595.0 (550.7–639.2)	669.7 (579.4–759.9)	0.2
Se	μmol per kg	6.6 (6.0–7.3)	7.1 (6.4–7.8)	0.2

*Data are means (±95% CI) taken over three replicate runs; P-values for significance of between-group differences were calculated by Mann-Whitney U-test. P < 0.05 is considered significant. Significant case-control differences are highlighted in bold*.

**Table 4B:** Metal concentrations in the CG of PDD cases and controls.

**Metal**	**Units**	**PDD (*n* = 9)**	**Controls (*n* = 27)**	***p*-value**
Na	mmol per kg	350.1 (286.5–413.7)	386.8 (340.1–433.4)	0.6
Mg	mmol per kg	22.2 (21.1–23.3)	22.8 (22.0–23.7)	0.5
K	mmol per kg	274.6 (258.9–290.3)	295.5 (277.7–313.4)	0.13
Ca	mmol per kg	6.0 (5.0–7.0)	7.2 (6.3–8.1)	0.3
Mn	μmol per kg	18.4 (15.8–21.1)	21.4 (20.2–22.6)	0.06
Fe	mmol per kg	3.8 (3.2–4.3)	3.5 (3.3–3.8)	0.5
Cu	μmol per kg	181.6 (152.5–210.8)	249.0 (216.4–281.6)	**0.009**
Zn	μmol per kg	746.5 (652.2–840.8)	845.8 (786.4–905.2)	0.13
Se	μmol per kg	7.7 (6.8–8.6)	8.4 (7.8–9.0)	0.3

*Data are means (±95% CI) taken over three replicate runs; P-values for significance of between-group differences were calculated by Mann-Whitney U-test. P < 0.05 is considered significant. This region includes controls from the Manchester and Newcastle AD cohorts. Significant case-control differences are highlighted in bold*.

**Table 4C:** Metal concentrations in the LC of PDD cases and controls.

**Metal**	**Units**	**PDD (*n* = 9)**	**Controls (*n* = 9)**	***p*-value**
Na	mmol per kg	213.7 (197.6–229.8)	196.8 (163.9–229.8)	0.5
Mg	mmol per kg	18.7 (17.8–19.6)	19.4 (18.6–20.2)	0.30
K	mmol per kg	244.4 (229.8–259.1)	253.3 (234.3–272.3)	0.5
Ca	mmol per kg	4.2 (2.9–5.4)	5.1 (4.8–5.4)	0.3
Mn	μmol per kg	14.7 (13.2–16.2)	16.7 (15.4–18.1)	0.02
Fe	mmol per kg	1.6 (1.4–1.7)	1.5 (1.3–1.7)	0.6
Cu	μmol per kg	168.8 (88.1–249.5)	214.7 (171.0–258.3)	0.7
Zn	μmol per kg	348.3 (339.3–357.4)	355.4 (294.6–416.2)	0.3
Se	μmol per kg	7.0 (6.5–7.6)	6.9 (6.3–7.6)	0.8

*Data are means (±95% CI) taken over three replicate runs; P-values for significance of between-group differences were calculated by Mann-Whitney U-test. P < 0.05 is considered significant*.

**Table 4D:** Metal concentrations in the HP of PDD cases and controls.

**Metal**	**Units**	**PDD (*n* = 9)**	**Controls (*n* = 27)**	***p*-value**
Na	mmol per kg	465.1 (426.1–504.1)	419.4 (350.5–488.3)	0.3
Mg	mmol per kg	24.8 (22.9–26.8)	26.7 (25.4–27.9)	0.1
K	mmol per kg	287.1 (247.0–327.1)	345.9 (315.5–376.2)	**0.04**
Ca	mmol per kg	9.5 (8.3–10.6)	8.5 (6.8–10.2)	0.2
Mn	μmol per kg	23.4 (10.7–26.1)	27.0 (25.1–28.9)	**0.04**
Fe	mmol per kg	4.1 (3.6–4.7)	4.0 (3.4–4.6)	0.3
Cu	μmol per kg	177.4 (120.8–234.0)	303.1 (274.1–332.2)	**0.0010**
Zn	μmol per kg	774.2 (623.7–924.8)	1095.5 (989.6–1201.4)	**0.009**
Se	μmol per kg	8.9 (7.5–10.4)	11.3 (10.5–12.0)	0.1

*Data are means (±95% CI) taken over three replicate runs; P-values for significance of between-group differences were calculated by Mann-Whitney U-test. P < 0.05 is considered significant. This region includes controls from the Auckland AD and HD cohorts. Significant case-control differences are highlighted in bold*.

**Table 4E:** Metal concentrations in the MED of PDD cases and controls.

**Metal**	**Units**	**PDD (*n* = 9)**	**Controls (*n* = 9)**	***p*-value**
Na	mmol per kg	278.2 (249.8–306.6)	268.0 (238.0–298.1)	0.3
Mg	mmol per kg	19.6 (19.1–20.2)	20.4 (19.7–21.0)	**0.03**
K	mmol per kg	204.2 (188.6–219.7)	215.8 (205.1–226.4)	0.1
Ca	mmol per kg	5.1 (4.2–6.1)	6.9 (4.7–9.1)	0.1
Mn	μmol per kg	15.8 (13.7–17.8)	19.8 (16.8–22.9)	**0.007**
Fe	mmol per kg	1.2 (1.0–1.4)	1.3 (1.2–1.4)	0.3
Cu	μmol per kg	158.1 (141.4–174.8)	236.6 (125.8–347.4)	**0.008**
Zn	μmol per kg	736.4 (643.2–829.7)	812.8 (704.2–921.5)	0.1
Se	μmol per kg	7.5 (7.0–8.0)	7.7 (6.9–8.4)	0.8

*Data are means (±95% CI) taken over three replicate runs; P-values for significance of between-group differences were calculated by Mann-Whitney U-test. P < 0.05 is considered significant. Significant case-control differences are highlighted in bold*.

**Table 4F:** Metal concentrations in the PVC of PDD cases and controls.

**Metal**	**Units**	**PDD (*n* = 9)**	**Controls (*n* = 9)**	***p*-value**
Na	mmol per kg	347.0 (299.0–395.0)	353.6 (316.0–391.2)	0.5
Mg	mmol per kg	22.9 (22.1–23.6)	24.2 (22.4–26.0)	0.2
K	mmol per kg	319.6 (301.4–337.9)	332.1 (288.8–375.5)	0.4
Ca	mmol per kg	6.7 (6.0–7.4)	11.7 (4.2–19.2)	0.2
Mn	μmol per kg	20.1 (17.9–22.2)	22.8 (20.2–25.4)	0.06
Fe	mmol per kg	5.1 (4.6–5.7)	4.8 (4.1–5.4)	0.5
Cu	μmol per kg	237.8 (215.6–259.9)	316.4 (239.9–393.0)	**0.008**
Zn	μmol per kg	938.8 (855.5–1022.0)	963.9 (817.3–1110.5)	0.3
Se	μmol per kg	9.9 (9.3–10.4)	9.9 (8.6–11.1)	0.8

*Data are means (±95% CI) taken over three replicate runs; P-values for significance of between-group differences were calculated by Mann-Whitney U-test. P < 0.05 is considered significant. Significant case-control differences are highlighted in bold*.

**Table 4G:** Metal concentrations in the MTG of PDD cases and controls.

**Metal**	**Units**	**PDD (*n* = 9)**	**Controls (*n* = 27)**	***p*-value**
Na	mmol per kg	439.3 (326.5–552.2)	353.9 (302.5–405.3)	0.10
Mg	mmol per kg	24.2 (22.3–26.0)	25.3 (23.1–27.5)	0.2
K	mmol per kg	299.9 (278.8–320.9)	359.7 (322.0–397.3)	**0.04**
Ca	mmol per kg	12.6 (8.6–16.6)	11.3 (7.4–15.3)	0.8
Mn	μmol per kg	16.5 (14.4–18.6)	21.9 (19.2–24.6)	**0.006**
Fe	mmol per kg	4.5 (4.0–5.0)	4.8 (4.3–5.2)	0.6
Cu	μmol per kg	225.4 (182.2–268.7)	337.7 (289.1–386.4)	**0.0050**
Zn	μmol per kg	1107.6 (940.4–1274.8)	1055.3 (948.2–1162.4)	0.6
Se	μmol per kg	9.5 (8.8–10.2)	12.6 (10.3–14.9)	0.3

*Data are means (±95% CI) taken over three replicate runs; P-values for significance of between-group differences were calculated by Mann-Whitney U-test. P < 0.05 is considered significant. This region includes controls from the Auckland AD and HD cohorts. Significant case-control differences are highlighted in bold*.

**Table 4H:** Metal concentrations in the CB of PDD cases and controls.

**Metal**	**Units**	**PDD (*n* = 9)**	**Controls (*n* = 9)**	***p*-value**
Na	mmol per kg	222.4 (161.6–283.1)	214.3 (184.8–243.9)	0.8
Mg	mmol per kg	20.7 (18.4–23.0)	20.1 (18.5–21.7)	0.9
K	mmol per kg	275.3 (226.5–324.1)	267.7 (224.8–310.5)	1.0
Ca	mmol per kg	4.2 (3.4–5.0)	4.4 (3.9–4.9)	0.4
Mn	μmol per kg	19.6 (14.1–25.1)	20.8 (16.6–25.0)	0.6
Fe	mmol per kg	4.2 (2.9–5.4)	3.8 (2.3–5.2)	0.6
Cu	μmol per kg	324.2 (256.5–391.8)	328.3 (161.5–495.2)	0.6
Zn	μmol per kg	736.4 (595.0–877.9)	698.4 (582.5–814.4)	0.8
Se	μmol per kg	8.0 (6.8–9.1)	7.1 (6.2–8.0)	0.2

*Data are means (±95% CI) taken over three replicate runs; P-values for significance of between-group differences were calculated by Mann-Whitney U-test. P < 0.05 is considered significant*.

**Table 4I:** Metal concentrations in the MCX of PDD cases and controls.

**Metal**	**Units**	**PDD (*n* = 9)**	**Controls (*n* = 26)**	***p*-value**
Na	mmol per kg	339.3 (217.2–461.4)	403.3 (251.4–555.2)	0.3
Mg	mmol per kg	20.7 (18.7–22.7)	26.4 (17.6–35.2)	**0.003**
K	mmol per kg	270.9 (218.3–323.5)	362.6 (225.1–500.1)	**0.03**
Ca	mmol per kg	6.5 (3.4–9.6)	7.0 (4.3–9.7)	0.4
Mn	μmol per kg	17.7 (14.4–21.1)	25.5 (16.4–34.5)	**0.003**
Fe	mmol per kg	4.5 (3.4–5.7)	5.8 (3.6–8.0)	0.07
Cu	μmol per kg	219.1 (174.5–263.6)	403.5 (171.7–635.3)	**0.0010**
Zn	μmol per kg	689.0 (470.6–907.5)	954.9 (585.0–1324.8)	0.06
Se	μmol per kg	7.9 (6.2–9.6)	12.7 (5.9–19.6)	**0.002**

*Data are means (±95% CI) taken over three replicate runs; P-values for significance of between-group differences were calculated by Mann-Whitney U-test. P < 0.05 is considered significant. This region includes controls from the Auckland AD and HD cohorts. Significant case-control differences are highlighted in bold*.

Like Cu, Zn, and Mn, Se contributes to anti-oxidation in the cell by acting as a regulatory cofactor of antioxidative enzymes such as glutathione peroxidases, which are involved in removal of hydrogen peroxide via the oxidation of reduced glutathione (GSH) to oxidized glutathione (GSSG). Previous investigations have observed upregulated glutathione peroxidase 4 (GPX4) around surviving dopaminergic cells in the PD SN despite decreased levels of the enzyme overall, suggesting that GPX4 may play a neuroprotective role in PD (Bellinger et al., 2011). Se is also a cofactor for several other antioxidative enzymes, including thioredoxin reductases, the first of which has been reported to be decreased in the SN of PD mouse models (Liu et al., 2013).

### Magnesium (Mg)

Like Se, Mg was also found to be decreased only in the MCX of PDD cases. Previous studies have reported decreased Mg in the CN but not in the FC, CN, CB (Uitti et al., 1989), SN (Uitti et al., 1989; Genoud et al., 2017), OCC or FG (Genoud et al., 2017). There are no previous reports on Mg in the PDD MCX. Mg concentrations in controls were found to be consistent with those previously reported in healthy aged brains within the MTG, CG, HP, PVC, midbrain, MED, and CB (Correia et al., 2014).

Restricting Mg intake in the rat over several generations has been shown to result in SN-exclusive dopaminergic neuron loss, although the effects on the MCX have not been reported (Oyanagi et al., 2006). Mg is also bound to a specific site in cytochrome c oxidase and regulates release of the complex into the mitochondria, as well as increasing NADH oxidation in the ETC (La Piana et al., 2008). As such, lowering of Mg may result in dysfunction of the mitochondrial ETC and diminished ATP production. This could be compounded by simultaneous decreases in Cu and Mg in the PDD MCX.

### Potassium (K)

Decreased K was found in three PDD brain regions investigated in this study; the MCX, HP, and MTG. A single previous investigation has reported no change in K levels in the PD FC, CN, SN, or CB (Uitti et al., 1989). This corresponds with the lack of change observed by ourselves in the PDD SN and CB. Other investigations on the MCX, HP, or MTG could not be found. Control concentrations were consistent with previously reported values in the MTG, HP, PVC, midbrain, MED, and CB, although slightly lower in the CG (~15% on average) (Ramos et al., 2016).

During neuronal apoptosis, K channels are upregulated, resulting in increased K efflux from the cell. This in turn promotes apoptosis itself, contributing to widespread neuronal loss in neurodegenerative conditions such as AD or stroke (Shah and Aizenman, 2014). Decreased K levels in the MCX, HP, and MTG may reflect and contribute to increased apoptosis in these areas.

### Iron (Fe)

Many investigations in the current literature report increased Fe in the substantia nigra (SN (Jellinger et al., 1990; Dexter et al., 1991; Ayton et al., 2013; Kim and Lee, 2014; Pyatigorskaya et al., 2015; Costa-Mallen et al., 2017; Genoud et al., 2017; Xuan et al., 2017). Some studies however have found no change in Fe levels within the SN of PD cases (Uitti et al., 1989; Loeffler et al., 1995; Galazka-Friedman et al., 1996). Where additional regions have been investigated, a lack of change has generally been observed, including in the white matter (Costa-Mallen et al., 2017); the OCC and FG (Genoud et al., 2017); and the FC (Uitti et al., 1989). Two sources have found no change in the raphe nucleus (Costa-Mallen et al., 2017; Xuan et al., 2017), the CB (Uitti et al., 1989; Dexter et al., 1991), the putamen (Jellinger et al., 1990; Costa-Mallen et al., 2017), and the cerebral cortex (Dexter et al., 1991; Costa-Mallen et al., 2017), and three reports have also observed no change in the CN (Uitti et al., 1989; Dexter et al., 1991; Xuan et al., 2017) and globus pallidus (Jellinger et al., 1990; Costa-Mallen et al., 2017; Xuan et al., 2017). There are however single reports of increased Fe in the putamen of PD patients (Xuan et al., 2017) and in the globus pallidus (Dexter et al., 1991). These observations largely agree with our own here, as we also observed no Fe changes in the PVC (located in the OCC), CB, or other cortical regions.

However, we also did not observe any change in the SN. There have been reports of increases in this region, with others observing no change in this region (Uitti et al., 1989; Jellinger et al., 1990; Dexter et al., 1991; Loeffler et al., 1995; Friedman et al., 2009; Ayton et al., 2013; Kim and Lee, 2014; Pyatigorskaya et al., 2015; Costa-Mallen et al., 2017; Genoud et al., 2017; Xuan et al., 2017). These discrepancies have prompted a consideration of factors which may contribute to such disparate findings. One possibility is that whilst there may be differences in labile Fe, this may not be reflected by a change in total Fe concentration, which was measured in this study (Friedman et al., 2009). Fe levels have also been observed to be highly heterogeneous between PD patients (Dashtipour et al., 2015). This may contribute to differing findings between studies, especially as investigations of human brain tissues are generally only possible on small cohorts of patients, either due to the limited availability of post-mortem tissue or the practical difficulties of imaging large numbers of volunteers. Of additional note, two studies which investigated the substantia nigra pars compacta (SNpc) and substantia nigra pars reticula (SNpr) separately, observed increased Fe in the former and no change in the latter (Jellinger et al., 1990; Costa-Mallen et al., 2017). This is contrary to observations of increased Fe in both the SNpc and SNpr elsewhere (Xuan et al., 2017). As such, it is possible that the area of the SN selected for study may affect observed metal levels. The effect of this possibility on the current study is indeterminable as different sub-regions of the SN were not distinguished during tissue dissection.

The Fe levels reported here in the control group are very consistent with those previously reported in healthy aged MTG, CG, HP, PVC, midbrain, MED, and CB (Ramos et al., 2014b). Interestingly, both the previous study and this investigation observed relatively low concentrations within the MED compared to other regions. Fe control levels are also similar with those reported in healthy LC and SN in another study, once wet-weight/dry-weight differences are accounted for (Zecca et al., 2004). The same study reported much lower Fe levels in the healthy LC compared to the SN, a finding which was also observed here, with around 3-fold higher Fe levels in the SN compared to the LC (4.6 mmol/kg or 0.26 mg/g v 1.5 mmol/kg or 0.08 mg/g; *p* < 0.0001, data not shown). This finding is interesting, considering the high concentrations of Fe-chelating neuromelanin in both the LC and the SN, although, decreased Fe-sequestration has been reported in neuromelanin in the LC than the SN previously, along with a lesser number of Fe deposits in the LC (Zucca et al., 2006).

The case group in the current study was on average 10 years younger than controls (mean 77.0 vs. 87.6 years respectively). This may be significant as several studies have observed Fe levels to increase with aging (Hebbrecht et al., 1999; Zecca et al., 2004; Pfefferbaum et al., 2009; Bilgic et al., 2012; Ramos et al., 2014b; Pirpamer et al., 2016). However, it appears this effect may level off eventually in older age, as a study investigating Fe levels in a cohort of individuals ranging from 70 to 103 years of age was unable to find any significant correlation between Fe levels and age (Exley et al., 2012). As the majority of individuals in the cohort used here were within this age range at time of death, this suggests that aging-related Fe accumulations should not have much effect on the current study.

Increased cerebral Fe levels have long been associated with different neurodegenerative diseases. Neurodegeneration with brain iron accumulation (NBIA) itself is a term used to describe a heterogeneous group of conditions characterized primarily by motor and cognitive dysfunction directly associated with increased Fe in the brain, particularly with the basal ganglia, independent of the presence of any other neurodegenerative disease (Wiethoff and Houlden, 2017). Increased cerebral Fe has likewise been associated with other neurodegenerative conditions including PD, AD, HD, and multiple sclerosis, among others (Ward et al., 2014). Fe is an essential metal, being involved in oxygen transport through the circulatory system, an essential metallic cofactor in the ETC during respiration, myelination of neurons, redox recycling, and neurotransmitter synthesis and release (Crichton, 2001). However, iron accumulation can have toxic effects, including production of ROS via the Fenton reaction and disruption of Fe-mediated processes such as those in the ETC and dopamine metabolism in the brain (Ward et al., 2014). As well as a possible role in several dementia pathogenic mechanisms, Fe has been proposed as a potential biomarker in neurodegenerative diseases such as AD and PD, as it is possible to image the metal in the brains of living individuals using MRI technology (Ward et al., 2014; Moller et al., 2019). Such imaging could allow for more definitive diagnoses ante-mortem, as well as monitoring of disease progression.

Neuromelanin-containing dopaminergic neurons within the SN have been reported to have particularly high levels of Fe in PD (Davies et al., 2014). Neuromelanin is a darkly-colored, Fe-chelating biopolymer that is present in the highest amounts in the SN and LC. It is produced during dopamine and norepinephrine metabolism, the former of which may contribute to the apparent vulnerability of areas such as the SN and LC, which have high numbers of dopaminergic neurons (Rabey and Hefti, 1990). However, in healthy brains, Fe levels are higher in glial cells than neuronal cells, with highest concentrations in oligodendrocytes, microglia, astrocytes, and then neurons in descending order (Reinert et al., 2019). This raises the importance of glial cells in possible Fe-mediated neurotoxicity as well as neuronal cells.

The majority of Fe in the brain is present bound to ferritin (Reinert et al., 2019). During aging, Fe binding to ferritin shifts from the heavy (H-ferritin) to the light (L-ferritin) form (Zecca et al., 2001). H-ferritin has higher antioxidant ferroxidase activity, so a decreased H/L-ferritin ratio may result in lower ROS clearance, whereas L-ferritin can better sequester Fe and prevent levels from reaching toxic levels (Harrison and Arosio, 1996). As such, the H/L-ferritin ratio is crucial to maintain Fe homeostasis. It has been reported that L-ferritin is decreased in PD brains compared to controls (Connor et al., 1995; Koziorowski et al., 2007). Fe-neuromelanin binding also increases with aging (Zecca et al., 2001). It has been proposed that neuromelanin may initially sequester Fe, but later release it in high levels upon reaching a critical concentration of Fe (Zecca et al., 2006). Together, these observations may contribute to a susceptibility to Fe-related neurodegeneration with aging, particularly in neuromelanin-rich areas of the brain which are vulnerable in PD.

As well as potential involvement in pathogenic mechanisms in PD, Fe may have potential as a biomarker of.

### Sodium (Na) and Calcium (Ca)

Na and Ca were not found to change in any region of the PDD brain investigated here. This supports previous reports of no change in Ca or Na levels in the PD FC, CN, SN, and CB (Uitti et al., 1989). Na levels in controls were similar to those reported previously in healthy aged MTG, CG, HP, PVC, midbrain, MED and CB (Ramos et al., 2016). However, Ca levels in the control group here appeared to be consistently higher than those observed in a previous study of these regions, ranging from an average of 44% higher in the midbrain to 126% higher in the MTG, with only the CB showing similar values (data not shown) (Correia et al., 2014). It's possible these discrepancies are accounted for by the wide variation in Ca levels observed in this cohort (see Supplementary Material E), but suggests that it would be difficult to observe any possible case-control differences in Ca that may be present in PDD using these samples.

## Conclusion

This study is the first to investigate differences in essential metals across multiple regions of the PDD brain. Although several studies looking at specific metals such as Cu, Fe, and Zn have been conducted in PD, these usually focus on the substantia nigra and do not distinguish between PD cases with and without dementia. PDD has not been as widely investigated as PD without dementia. The observations from this study confirm that PDD is driven by increased oxidative stress and mitochondrial dysfunction due to a loss of antioxidative and ETC enzyme metallomic co-factors including Cu, Mn, Zn, Se, K, and Mg. This includes widespread Cu decreases in PDD brains, reported for the first time here, affecting seven of the nine regions investigated, including the SN, CG, HP, MED, PVC, MTG, and MCX. This report also includes the first direct comparison of multiple essential metals across several brain regions in AD and PDD – observing equivalent decreases in Cu levels in the CG, HP, MTG, and MCX, as well as the relative sparing of the CB in PDD compared to AD. These findings indicate both shared mechanisms and regional variations between these two conditions that have not been previously observed.

## Data Availability Statement

The raw data supporting the conclusions of this article will be made available by the authors, without undue reservation.

## Ethics Statement

The studies involving human participants were reviewed and approved by Manchester REC (09/H0906/52+5), NZ Neurological Foundation Douglas Human Brain Bank, and the UK MRC Brain Bank Network. The patients/participants provided their written informed consent to participate in this study.

## Author Contributions

MS designed and performed research, analyzed and interpreted data, and wrote the first draft and subsequent drafts of the manuscript. SC performed research, analyzed data, and read and revised the manuscript. SC and NH performed research and revised the manuscript. FR, NH, and RU read and revised the manuscript. GC conceived, designed and supervised research, analyzed and interpreted data, wrote the manuscript, and bears overall responsibility for the integrity of the study and of the manuscript. JX performed research on previous Auckland AD cohort. SP performed research on previous HD cohort. All authors contributed to the article and approved the submitted version.

## Conflict of Interest

The authors declare that the research was conducted in the absence of any commercial or financial relationships that could be construed as a potential conflict of interest.
