# Supplementary material for: Widespread Decreases in Cerebral Copper Are Common to Parkinson's Disease Dementia and Alzheimer's Disease Dementia
Source: Front Aging Neurosci. 2021 Mar 3;13:641222. doi: 10.3389/fnagi.2021.641222 (PMC7966713; doi:10.3389/fnagi.2021.641222)
Supplement: Supplementary file 1 [file Data_Sheet_1.zip › PDD Paper - Supplementary Material A (Cohort Characteristics).docx]

Supplementary Material A

## Supplementary Table 1: Characteristics of individuals in the PDD cohort

| Code | Sex | Age at death | Clinical diagnosis | a-syn Braak stage | Tau Braak stage | Post-Mortem Delay (hours) | Whole-brain weight (g) | CERAD | Thal | Disease Duration (years) |
| --- | --- | --- | --- | --- | --- | --- | --- | --- | --- | --- |
| PDD1 | Male | 65 | PDD | 5 | 0 | 39 | 1642 | 0 | 2 | 7 |
| PDD2 | Male | 80 | PDD | 6 | 2 | 26 | 1165 | 0 | 0 | 11 |
| PDD3 | Male | 75 | PDD | 6 | 1 | 48 | 1399 | 0 | 1 | 6 |
| PDD4 | Female | 93 | PDD | 6 | 3 | 10 | 1222 | 1 | 3 | 15 |
| PDD5 | Male | 66 | PDD | 6 | 4 | 27 | 1371 | 2 | 5 | 17 |
| PDD6 | Female | 80 | PDD | 6 | 2 | 20 | 1152 | 1 | 3 | 23 |
| PDD7 | Male | 80 | PDD | 5 | 2 | 31 | 1402 | 0 | 0 | 13 |
| PDD8 | Female | 78 | PDD | 6 | 3 | 47 | 1386 | 0 | 2 | 10 |
| PDD9 | Female | 76 | PDD | 6 | 1 | 9 | 960 | 0 | 2 | 22 |
| C1 | Female | 82 | No dementia or brain disease | 0 | 2 | 15 | 946 | 0 | - | N/A |
| C2 | Female | 95 | No dementia or brain disease | 0 | 3 | 28 | 1126 | 1 | 2 | N/A |
| C3 | Male | 91 | No dementia or brain disease | 0 | 2 | 18 | 1338 | 0 | 2 | N/A |
| C4 | Male | 85 | No dementia or brain disease | 0 | 2 | 29 | 989 | 1 | 3 | N/A |
| C5 | Male | 79 | No dementia or brain disease | 0 | 2 | 25 | 1331 | 0 | 1 | N/A |
| C6 | Female | 87 | No dementia or brain disease | 0 | 2 | 15 | 978 | 0 | 3 | N/A |
| C7 | Female | 94 | No dementia or brain disease | 0 | 2 | 40 | 1158 | 0 | 5 | N/A |
| C8 | Female | 88 | No dementia or brain disease | 0 | 3 | 23 | 1033 | 0 | 2 | N/A |
| C9 | Male | 87 | No dementia or brain disease | 0 | 1 | 48 | 1301 | 0 | 3 | N/A |

Abbreviation: N/A = not applicable. Presence of a dash – indicates that data is not available.

## Supplementary Table 2: Recorded comorbidities, causes of death, and neuropathological diagnoses of PDD cohort

| Code | Combordities | Cause of Death | Neuropathological Diagnosis |
| --- | --- | --- | --- |
| PDD1 | Depression; anxiety; halluci-tions | Dementia with Lewy Bodies | Limbic type of Lewy body disease |
| PDD2 | Depression; epilepsy | Bronchopneumonia; PD | Neocortical Lewy body disease |
| PDD3 | Anxiety and depression | Bronchopneumonia; PD | Parkinson's disease; diffuse neocortical Lewy body pathology |
| PDD4 | Osteoperosis; asymptomatic CVD; osteoarthritis | Myocardial infarction; PD | Parkinson's disease; diffuse neocortical Lewy body pathology |
| PDD5 | Actinic keratosis; Depression; Anxiety; Carcinoma | Bronchopneumonia | Neocortical Lewy body disease |
| PDD6 | Depression; hyperthyroid; epilepsy | PD | Neocortical Lewy body disease |
| PDD7 | Depression; anxiety; COPD | Stroke | Limbic type of Lewy body disease |
| PDD8 | Osteoporosis; RBD; hallucinations | Abdominal obstruction; PD | Neocortical lewy body disease |
| PDD9 | Hallucinations; epilepsy; arthritis; glaucoma | PD | Neocortical Lewy body disease |
| C1 | Hypertension; hyperlipidaemia; alcoholism | Lung cancer | Neuritic Braak stage II |
| C2 | Hypertension; hypothyroidism; CKD | Pneumonia; chronic kidney disease | Low AD neuropathologic change |
| C3 | Postural hypotension; bradychardia; scoliosis | Unknown | Low AD neuropathologic change |
| C4 | Pacemaker fitted following aortic valve replacement; bradychardia | Lobar pneumonia | Low AD neuropathologic change |
| C5 | Type 2 diabetes; ischaemic heart disease; family history of PD | Brainstem stroke; bronchopneumonia | Low AD neuropathologic change |
| C6 | CKD, gout, osteoarthritis, polymalgia rheumatica; sleep apnoea; atrial fibrillation; gout | Acutely ischaemic left leg – unsalvageable; cellulitis left foot; atrial fibrillation; cerebrovascular event; COPD; peripheral vascular disease; heart failure | Low AD neuropathologic change; cerebellar infarct |
| C7 | CKD stage 3, osteoperosis, hypercholesterolemia; postural hypotension | Respiratory failure; bronchopneumonia; fractured sternum due to fall | Low AD neuroprehologic change; CAA type II |
| C8 | Osteoperosis, type 2 diabetes, atrial fibrillation, hypertension; short term memory problems (determined to be stress-related) | Old age | Intermediate AD neuropathologic change |
| C9 | Hypertension; arthritis; minor stroke; prostatism; atrial fibrillation | Congestive cardiac failure; atrial fibrillation; mitral regurgitation; chronic kidney disease Stage 5 | Low AD neuropathologic change |

## Supplementary Table 3: Characteristics of individuals in the Auckland Alzheimer’s disease (AD) cohort

| Code | Sex | Age at death | Clinical diagnosis | Braak stage (AD) | APOE  Status | Post-Mortem Delay (hours) | Whole-brain weight (g) | Brain pH | Cause of Death |
| --- | --- | --- | --- | --- | --- | --- | --- | --- | --- |
| AD1 | M | 60 | Alzheimer's Disease | 6 | - | 7.0 | 1020 | 7.0 | Alzheimer’s disease |
| AD2 | F | 62 | Alzheimer's disease | 6 | - | 6.0 | 831 | 6.0 | Alzheimer’s disease |
| AD3 | F | 63 | Alzheimer's disease | 6 | - | 7.0 | 1080 | 7.0 | Bronchopneumonia |
| AD4 | F | 70 | Alzheimer's disease | 5 | - | 7.0 | 1044 | 7.0 | Lung cancer |
| AD5 | M | 73 | Alzheimer's disease | 4 | - | 4.0 | 1287 | 4.0 | GI haemorrhage |
| AD6 | F | 74 | Alzheimer's disease | 5 | - | 8.5 | 1062 | 8.5 | Metastatic cancer |
| AD7 | M | 74 | Alzheimer's disease | 6 | - | 12.0 | 1355 | 12.0 | Pseudomonas bacteraemia |
| AD8 | M | 77 | Alzheimer's disease | 6 | - | 4.5 | 1180 | 4.5 | Myocardial infarction |
| AD9 | M | 80 | Alzheimer's disease | 5 | - | 5.5 | 1039 | 5.5 | Bronchopneumonia/ pulmonary oedema |
| C10 | M | 61 | No dementia or brain disease | 0 | - | 7.0 | 1258 | 7.0 | Ischaemic heart disease |
| C11 | F | 64 | No dementia or brain disease | 0 | - | 5.5 | 1260 | 5.5 | Pulmonary embolism |
| C12 | F | 63 | No dementia or brain disease | 0 | - | 12.0 | 1280 | 12.0 | Ruptured aorta |
| C13 | F | 72 | No dementia or brain disease | 0 | - | 9.0 | 1230 | 9.0 | Emphysema |
| C14 | M | 66 | No dementia or brain disease | 0 | - | 9.0 | 1461 | 9.0 | Ischaemic heart disease |
| C15 | F | 76 | No dementia or brain disease | 2 | - | 12.0 | 1094 | 12.0 | Metastatic carcinoma |
| C16 | M | 73 | No dementia or brain disease | 0 | - | 13.0 | 1315 | 13.0 | Ischaemic heart disease |
| C17 | M | 78 | No dementia or brain disease | 0 | - | 7.5 | 1260 | 7.5 | Ruptured aortic aneurysm |
| C18 | M | 78 | No dementia or brain disease | 0 | - | 12.0 | 1416 | 12.0 | Ruptured MI |

Presence of a dash – indicates that data is not available.

## Supplementary Table 4: Characteristics of individuals in the Manchester cohort

| Code | Sex | Age at death | Clinical diagnosis | Braak stage (AD) | APOE  Status | Post-Mortem Delay (hours) | Whole-brain weight (g) | Brain pH | | Cause of Death | |
| --- | --- | --- | --- | --- | --- | --- | --- | --- | --- | --- | --- |
| AD10 | M | 88 | Alzheimer's Disease | V | 3/4 | 75 | 1027 | | - | | - |
| AD11 | M | 69 | Alzheimer's disease | VI | 3/3 | 96 | 1160† | | - | | - |
| AD12 | M | 65 | Alzheimer's disease | VI | 3/3 | *72** | - | | - | | - |
| AD13 | F | 61 | Alzheimer's disease | VI | 3/4 | 130 | 1120 | | - | | - |
| AD14 | F | 89 | Alzheimer's disease | V-VI | 3/3 | 72 | - | | - | | - |
| AD15 | M | 76 | Alzheimer's disease | V-VI | 3/4 | *96** | 1359 | | - | | - |
| AD16 | M | 83 | Alzheimer's disease | IV-V | 3/3 | 96 | 1046† | | - | | - |
| AD17 | F | 88 | Alzheimer's disease | VI | 3/3 | 72 | 900† | | - | | - |
| AD18 | M | 87 | Alzheimer's disease | IV | 3/4 | 49 | 1066 | | - | | - |
| C19 | F | 92 | No dementia or brain disease | II | 3/4 | 37 | 1080† | | - | | Myocardial infarction |
| C20 | F | 87 | No dementia or brain disease | I-II | 3/3 | 39 | 1160† | | - | | Anteroseptal myocardial infarction; coronary atherosclerosis; left ventricular hypertrophy |
| C21 | M | 89 | No dementia or brain disease | II | 3/3 | 27 | 1400† | | - | | Multi-organ failure, septicaemia, cellulitis, heart failure |
| C22 | M | 95 | No dementia or brain disease | I-II | 3/3 | 12 | 1200† | | - | | Prostatic carcinoma |
| C23 | F | 87 | No dementia or brain disease | 0-I | 3/4 | 24 | 1152 | | - | | Cardiac failure, COPD, renal impairment, osteoarthritis |
| C24 | M | 84 | No dementia or brain disease | I | 3/3 | 69.5 | 1494 | | 5.84 | | - |
| C25 | F | 90 | No dementia or brain disease | 0-I | 3/3 | 39 | 1050 | | - | | Frailty of old age |
| C26 | F | 82 | No dementia or brain disease | 0-I | 3/3 | 61 | 1020† | | - | | Metastatic ovarian cancer |
| C27 | F | 94 | No dementia or brain disease | I-II | 3/3 | 70 | 1276 | | 5.87 | | Carcinoma of the appendix |

Presence of a dash – indicates that data is not available.

## Supplementary Table 5: Characteristics of individuals in the Newcastle cohort

| Code | Gender | Age at death | Clinical diagnosis | Braak stage (AD) | APOE  Status | Post-Mortem Delay (hours) | Whole-brain weight (g) | CERAD | Brain pH | Cause of Death |
| --- | --- | --- | --- | --- | --- | --- | --- | --- | --- | --- |
| AD19 | M | 81 | Alzheimer's Disease | 6 | 3/4 | 41 | 1351 | High | 7.08 | Chest infection |
| AD20 | M | 87 | Alzheimer's disease | 6 | 3/4 | 21 | 1200 | High | 6.23 | Pneumonia |
| AD21 | F | 80 | Alzheimer's disease | 6 | 4/4 | 10 | 985 | High | 5.35 | Bronchopneumonia & Alzheimer’s disease |
| AD22 | F | 95 | Alzheimer's disease | 6 | 3/4 | 23 | 968 | Moderate | 6.21 | Stroke |
| AD23 | M | 84 | Alzheimer's disease | 6 | 3/4 | 40 | 1166 | Moderate | 6.70 | - |
| AD24 | M | 86 | Alzheimer's disease | 6 | 2/4 | 9 | 1066 | High | 6.47 | Bronchopneumonia & metastatic bladder cancer |
| AD25 | M | 90 | Alzheimer's disease | 6 | 2/3 | 13 | 1303 | High | 6.43 | Frailty old age & chest infection & Alzheimer’s disease |
| AD26 | M | 86 | Alzheimer's disease | 6 | 3/3 | 40 | 1043 | High | 6.34 | - |
| AD27 | F | 70 | Alzheimer's disease | 6 | 4/4 | 24 | 959 | High | 6.46 | Alzheimer’s disease |
| C28 | M | 88 | No dementia or brain disease | 1 | 2/3 | 28 | 1400 | None | 6.30 | Chronic lymphocytic leukaemia |
| C29 | M | 80 | No dementia or brain disease | 2 | 3/3 | 16 | 1406 | None | 6.36 | Prostate cancer |
| C30 | M | 88 | No dementia or brain disease | 2 | 3/3 | 26 | 1362 | None | 6.10 | Chronic COPD |
| C31 | F | 81 | No dementia or brain disease | 1 | 3/3 | 40 | - | - | - | - |
| C32 | M | 94 | No dementia or brain disease | 2 | 3/4 | 25 | 1175 | None | 6.66 | Bronchopneumonia |
| C33 | F | 91 | No dementia or brain disease | 2 | 3/3 | 14 | 1238 | None | 6.55 | Aspiration pneumonia + severe inoperable pharyngeal pouch |
| C34 | M | 76 | No dementia or brain disease | 2 | 3/4 | 34 | 1363 | None | 6.52 | Pneumonic exacerbation of COPD |
| C35 | F | 81 | No dementia or brain disease | 1 | - | 19 | 1064 | None | 6.09 | Metastatic liver cancer |
| C36 | M | 92 | No dementia or brain disease | 2 | 2/3 | 9 | 1319 | None | 6.28 | Uro-sepsis + metastatic prostate cancer, liver and adrenal metastasis + CKD3 |

Presence of a dash – indicates that data is not available.

## Supplementary Table 6: Characteristics of individuals in the Auckland Huntington’s disease (HD) cohort

| Code | Sex | Age at death | Clinical diagnosis | Braak stage (AD) | APOE  Status | Post-Mortem Delay (hours) | Whole-brain weight (g) | Brain pH | Cause of Death |
| --- | --- | --- | --- | --- | --- | --- | --- | --- | --- |
| HD1 | 1 | 80 | Huntington’s disease | - | - | 9 | 1105 | - | Bronchopneumonia |
| HD2 | 2 | 67 | Huntington’s disease | - | - | 9 | 1139 | - | Myocardial infarction |
| HD3 | 2 | 59 | Huntington’s disease | - | - | 7 | 787 | - | Bronchopneumonia |
| HD4 | 2 | 62 | Huntington’s disease | - | - | 11 | 826 | - | Bronchopneumonia |
| HD5 | 1 | 62 | Huntington’s disease | - | - | 9 | 992 | - | Huntington’s disease |
| HD6 | 1 | 83 | Huntington’s disease | - | - | 13 | 1168 | - | Bronchopneumonia |
| HD7 | 1 | 58 | Huntington’s disease | - | - | 14 | 1497 | - | Bronchopneumonia |
| HD8 | 1 | 51 | Huntington’s disease | - | - | 15 | 1200 | - | Bronchopneumonia |
| HD9 | 1 | 65 | Huntington’s disease | - | - | 14 | 1224 | - | Renal failure |
| C37 | 1 | 63 | No dementia or brain disease | - | - | 9 | 1432 | - | Ischaemic heart disease |
| C38 | 1 | 61 | No dementia or brain disease | - | - | 7 | 1258 | - | Ischaemic heart disease |
| C39 | 2 | 77 | No dementia or brain disease | - | - | 12 | 1227 | - | Ischaemic heart disease |
| C40 | 2 | 73 | No dementia or brain disease | - | - | 13 | 1210 | - | Coronary atherosclerosis |
| C41 | 1 | 73 | No dementia or brain disease | - | - | 13 | 1315 | - | Ischaemic heart disease |
| C42 | 1 | 49 | No dementia or brain disease | - | - | 13 | 1495 | - | Ischaemic heart disease |
| C43 | 1 | 66 | No dementia or brain disease | - | - | 15 | 1360 | - | Ischaemic heart disease |
| C44 | 2 | 64 | No dementia or brain disease | - | - | 6.5 | 1260 | - | Pulmonary embolism |
| C45 | 1 | 81 | No dementia or brain disease | - | - | 7 | 1343 | - | Coronary atherosclerosis |

Presence of a dash – indicates that data is not available.
