# Supplementary material for: Widespread Decreases in Cerebral Copper Are Common to Parkinson's Disease Dementia and Alzheimer's Disease Dementia
Source: Front Aging Neurosci. 2021 Mar 3;13:641222. doi: 10.3389/fnagi.2021.641222 (PMC7966713; doi:10.3389/fnagi.2021.641222)
Supplement: Supplementary file 1 [file Data_Sheet_1.zip › PDD Paper - Supplementary Material B (Blank & Standard Data).docx]

Supplementary Material B

**Contents**

[**Supplementary Table B1: Metal concentrations in digestion blanks in PDD Cohort** 2](#_Toc33524327)

[**Supplementary Table B2: Detection limit, limit of quantitation and background equivalent concentration for each physiological metal measured in the PDD cohort** 11](#_Toc33524328)

[**Supplementary Figure B1: Standard curves for each physiological metal measured in the PDD cohort** 17](#_Toc33524329)

[**Supplementary Table B3: Metal concentrations in digestion blanks in Manchester & Newcastle cohorts** 31](#_Toc33524330)

[**Supplementary Table B4: Detection limit, limit of quantitation and background equivalent concentration for each physiological metal measured in the additional cohorts** 34](#_Toc33524331)

[**Supplementary Figure B2: Standard curves for each physiological metal measured in the additional cohorts** 36](#_Toc33524332)

## **Supplementary Table B1: Metal concentrations in digestion blanks in PDD Cohort**

| Run | ^23^Na (µg/L) | ^24^Mg (µg/L) | 39 K (µg/L) | 44 Ca (µg/L) | 55 Mn (µg/L) | 56 Fe (µg/L) | 63 Cu (µg/L) | 66 Zn (µg/L) | 78 Se (µg/L) |
| --- | --- | --- | --- | --- | --- | --- | --- | --- | --- |
| MTC  *Run 1* | | | | | | | | | |
| Digestion Blank 1 | 2.80 | 0.51 | <0.00 | 3.73 | <0.00 | 0.39 | 0.04 | 0.65 | <0.00 |
| Digestion Blank 2 | 3.84 | 0.90 | <0.00 | 3.12 | <0.00 | 0.21 | 0.00 | 0.01 | 0.01 |
| Lowest Sample | 6762.80 | 464.23 | 9400.89 | 288.25 | 0.74 | 171.52 | 10.94 | 54.27 | 0.53 |
| % highest blank/ lowest sample | **0.06** | **0.19** | **<0.0000** | **1.29** | **<0.0000** | **0.23** | **0.38** | **1.19** | **1.81** |
| *Run 2* | | | | | | | | | |
| Digestion Blank 1 | 2.71 | 0.48 | <0.00 | <0.00 | 0.04 | 5.55 | 0.10 | 0.50 | <0.00 |
| Digestion Blank 2 | 3.42 | 0.02 | <0.00 | <0.00 | <0.00 | 0.14 | 0.07 | <0.00 | <0.00 |
| Lowest Sample | 6255.47 | 438.12 | 8677.22 | 272.52 | 0.74 | 151.70 | 11.52 | 56.50 | 0.46 |
| % highest blank/ lowest sample | **0.05** | **0.11** | **<0.0000** | **<0.0000** | **5.04** | **3.66** | **0.84** | **0.88** | **<0.0000** |
| *Run 3* | | | | | | | | | |
| Digestion Blank 1 | 62.11 | 24.79 | <0.00 | 5.51 | <0.00 | 0.39 | <0.00 | 0.26 | <0.00 |
| Digestion Blank 2 | 58.43 | 27.21 | <0.00 | 7.38 | <0.00 | 1.39 | <0.00 | 1.89 | <0.00 |
| Lowest Sample | 4456.93 | 338.17 | 7045.70 | 188.46 | 0.56 | 142.77 | 7.44 | 39.18 | 0.33 |
| % highest blank/ lowest sample | **1.39** | **8.04** | **<0.0000** | **3.91** | **<0.0000** | **0.97** | **<0.0000** | **4.82** | **<0.0000** |
| CB  *Run 1* | | | | | | | | | |
| Digestion Blank 1 | 5.06 | 1.78 | <0.00 | 38.43 | 0.00 | 2.04 | 0.01 | 0.05 | 0.00 |
| Digestion Blank 2 | 3.38 | 0.38 | <0.00 | 1.38 | 0.01 | 0.34 | 0.07 | 0.17 | <0.00 |
| Lowest Sample | 5794.57 | 587.01 | 10558.52 | 227.17 | 0.91 | 150.67 | 11.31 | 47.03 | 0.55 |
| % highest blank/ lowest sample | **0.09** | **0.30** | **<0.000** | **16.92** | **1.34** | **1.35** | **0.65** | **0.37** | **0.01** |
| *Run 2* | | | | | | | | | |
| Digestion Blank 1 | 2.71 | 0.48 | <0.00 | <0.00 | 0.04 | 5.55 | 0.10 | 0.50 | <0.00 |
| Digestion Blank 2 | 3.42 | 0.02 | <0.00 | <0.00 | <0.00 | 0.14 | 0.07 | <0.00 | <0.00 |
| Lowest Sample | 6255.47 | 438.12 | 8677.22 | 272.52 | 0.74 | 151.70 | 11.52 | 56.50 | 0.46 |
| % highest blank/ lowest sample | **0.05** | **0.11** | **<0.0000** | **<0.0000** | **5.04** | **3.66** | **0.84** | **0.88** | **<0.0000** |
| *Run 3* | | | | | | | | | |
| Digestion Blank 1 | 44.37 | 27.28 | <0.00 | 1.60 | <0.00 | 0.06 | <0.00 | <0.00 | <0.00 |
| Digestion Blank 2 | 38.17 | 16.65 | <0.00 | 10.13 | 0.03 | 2.24 | <0.00 | 0.90 | <0.00 |
| Lowest Sample | 4195.60 | 464.36 | 8090.52 | 147.02 | 0.67 | 84.20 | 7.20 | 34.27 | 0.46 |
| % highest blank/ lowest sample | **1.06** | **5.87** | **<0.0000** | **6.89** | **4.96** | **2.67** | **<0.0000** | **2.62** | **<0.0000** |
| CG  *Run 1* | | | | | | | | | |
| Digestion Blank 1 | 0.16 | <0.00 | <0.00 | 2.80 | <0.00 | <0.00 | <0.00 | <0.00 | <0.00 |
| Digestion Blank 2 | 0.28 | 0.57 | <0.00 | 65.82 | 0.00 | <0.00 | <0.00 | <0.00 | <0.00 |
| Lowest Sample | 6601.59 | 502.38 | 9216.55 | 221.33 | 0.84 | 160.95 | 8.63 | 41.76 | 0.47 |
| % highest blank/ lowest sample | **0.004** | **0.11** | **<0.0000** | **29.74** | **0.13** | **<0.0000** | **<0.0000** | **<0.0000** | **<0.0000** |
| *Run 2* | | | | | | | | | |
| Digestion Blank 1 | <0.00 | <0.00 | <0.00 | 16.20 | 0.05 | 4.33 | 0.23 | <0.00 | <0.00 |
| Digestion Blank 2 | <0.00 | <0.00 | <0.00 | <0.00 | 0.02 | 1.11 | <0.00 | <0.00 | <0.00 |
| Lowest Sample | 6889.55 | 507.92 | 10353.11 | 247.46 | 0.81 | 161.52 | 10.72 | 40.01 | 0.50 |
| % highest blank/ lowest sample | **<0.0000** | **<0.0000** | **<0.0000** | **6.55** | **6.46** | **2.68** | **2.11** | **<0.0000** | **<0.0000** |
| *Run 3* | | | | | | | | | |
| Digestion Blank 1 | 11.79 | <0.00 | 1.74 | 10.51 | 0.07 | 6.86 | 0.03 | 0.13 | 0.00 |
| Digestion Blank 2 | 6.66 | <0.00 | 2.33 | 5.13 | <0.00 | 0.18 | <0.00 | 0.10 | <0.00 |
| Lowest Sample | 6161.79 | 462.58 | 8600.54 | 188.47 | 0.85 | 154.50 | 7.60 | 43.71 | 0.51 |
| % highest blank/ lowest sample | **0.19** | **<0.0000** | **0.03** | **5.57** | **8.76** | **4.44** | **0.37** | **0.29** | **0.36** |
| HP  *Run 1* | | | | | | | | | |
| Digestion Blank 1 | 2.54 | 8.35 | <0.00 | 8.23 | 0.01 | 0.47 | <0.00 | 0.07 | <0.00 |
| Digestion Blank 2 | 6.57 | 7.76 | <0.00 | 21.05 | 0.00 | 0.38 | <0.00 | 1.08 | <0.00 |
| Lowest Sample | 6064.09 | 424.01 | 8729.82 | 192.02 | 0.88 | 131.60 | 6.18 | 44.49 | 0.49 |
| % highest blank/ lowest sample | **0.11** | **1.97** | **<0.0000** | **10.96** | **1.58** | **0.36** | **<0.0000** | **2.42** | **<0.0000** |
| Run 2 | | | | | | | | | |
| Digestion Blank 1 | 5.07 | 12.81 | <0.00 | 6.93 | 0.01 | 0.40 | 0.03 | 0.21 | <0.00 |
| Digestion Blank 2 | 4.32 | 10.49 | <0.00 | 8.18 | 0.04 | 1.45 | 0.02 | 0.20 | 0.00 |
| Lowest Sample | 6507.34 | 481.95 | 8864.68 | 249.53 | 0.97 | 143.61 | 8.04 | 55.63 | 0.56 |
| % highest blank/ lowest sample | **0.08** | **2.66** | **<0.0000** | **3.28** | **3.81** | **1.01** | **0.38** | **0.38** | **0.25** |
| *Run 3* | | | | | | | | | |
| Digestion Blank 1 | <0.00 | 0.30 | <0.00 | <0.00 | 0.01 | 0.57 | 0.01 | <0.00 | 0.00 |
| Digestion Blank 2 | <0.00 | 0.35 | <0.00 | 0.89 | 0.03 | 0.94 | 0.07 | 0.11 | <0.00 |
| Lowest Sample | 7502.99 | 509.60 | 9530.53 | 246.46 | 1.03 | 165.44 | 7.44 | 57.60 | 0.55 |
| % highest blank/ lowest sample | **<0.0000** | **0.07** | **<0.0000** | **0.36** | **3.38** | **0.57** | **0.89** | **0.20** | **0.06** |
| LC  *Run 1* | | | | | | | | | |
| Digestion Blank 1 | <0.00 | 6.49 | <0.00 | 2.32 | 0.04 | 0.75 | <0.00 | 0.02 | <0.00 |
| Digestion Blank 2 | 26.94 | 5.06 | <0.00 | 4.21 | 0.03 | 0.59 | 0.63 | 0.32 | <0.00 |
| Lowest Sample | 5071.31 | 423.98 | 8749.34 | 178.42 | 0.89 | 84.66 | 8.26 | 24.15 | 0.44 |
| % highest blank/ lowest sample | **0.53** | **1.53** | **<0.0000** | **2.36** | **4.07** | **0.89** | **7.58** | **1.30** | **<0.0000** |
| *Run 2* | | | | | | | | | |
| Digestion Blank 1 | 0.45 | 10.11 | <0.00 | 2.22 | 0.15 | 2.46 | 0.25 | 0.20 | <0.00 |
| Digestion Blank 2 | 4.48 | 11.42 | <0.00 | 42.20 | 0.28 | 15.87 | 0.13 | 0.62 | 0.003 |
| Lowest Sample | 5475.42 | 373.75 | 7702.90 | 165.35 | 0.90 | 85.96 | 9.56 | 28.36 | 0.41 |
| % highest blank/ lowest sample | **0.08** | **3.06** | **<0.0000** | **25.52** | **30.79** | **18.46** | **2.64** | **2.19** | **0.67** |
| *Run 3* | | | | | | | | | |
| Digestion Blank 1 | <0.00 | 0.34 | <0.00 | <0.00 | 0.14 | 14.81 | 0.04 | <0.00 | <0.00 |
| Digestion Blank 2 | <0.00 | 0.44 | <0.00 | <0.00 | 0.03 | 2.14 | 0.02 | 0.00 | 0.01 |
| Lowest Sample | 5665.10 | 583.71 | 11616.12 | 224.52 | 1.02 | 91.96 | 9.45 | 42.59 | 0.74 |
| % highest blank/ lowest sample | **<0.0000** | **0.08** | **<0.0000** | **<0.0000** | **13.83** | **16.11** | **0.39** | **0.01** | **1.24** |
| MED  *Run 1* | | | | | | | | | |
| Digestion Blank 1 | 3.38 | 1.72 | <0.00 | 13.74 | 0.42 | 4.11 | 0.52 | 2.13 | <0.00 |
| Digestion Blank 2 | <0.00 | <0.00 | <0.00 | 1.28 | <0.00 | <0.00 | 0.02 | 0.34 | <0.00 |
| Lowest Sample | 6237.57 | 554.45 | 9257.73 | 200.11 | 0.89 | 73.35 | 10.68 | 54.57 | 0.68 |
| % highest blank/ lowest sample | **0.05** | **0.31** | **<0.0000** | **6.86** | **47.20** | **5.61** | **4.86** | **3.90** | **<0.0000** |
| *Run 2* | | | | | | | | | |
| Digestion Blank 1 | 0.36 | 0.50 | <0.00 | 13.48 | <0.00 | 0.84 | 0.04 | 0.26 | <0.00 |
| Digestion Blank 2 | 0.31 | 0.35 | <0.00 | <0.00 | <0.00 | 0.25 | 0.01 | 0.90 | 0.00 |
| Lowest Sample | 6295.84 | 574.99 | 9397.09 | 231.75 | 0.86 | 52.25 | 5.78 | 41.49 | 0.61 |
| % highest blank/ lowest sample | **0.01** | **0.09** | **<0.0000** | **5.82** | **<0.0000** | **1.62** | **0.67** | **2.18** | **0.36** |
| *Run 3* | | | | | | | | | |
| Digestion Blank 1 | 0.88 | 3.22 | <0.00 | 5.23 | <0.00 | 1.34 | 0.05 | 0.28 | 0.00 |
| Digestion Blank 2 | 6.45 | 0.48 | <0.00 | 1.31 | 0.04 | 3.73 | <0.00 | <0.00 | 0.00 |
| Lowest Sample | 6659.22 | 578.75 | 9598.33 | 263.51 | 0.99 | 72.62 | 11.63 | 48.27 | 0.56 |
| % highest blank/ lowest sample | **0.10** | **0.56** | **<0.0000** | **1.98** | **3.91** | **5.14** | **0.47** | **0.58** | **0.73** |
| PVC  *Run 1* | | | | | | | | | |
| Digestion Blank 1 | <0.00 | <0.00 | <0.00 | 4.37 | 0.02 | 0.19 | 0.08 | 0.11 | <0.00 |
| Digestion Blank 2 | 0.23 | 0.61 | <0.00 | 4.44 | <0.00 | <0.00 | 0.06 | 0.87 | <0.00 |
| Lowest Sample | 5571.40 | 497.75 | 11780.77 | 216.17 | 0.80 | 224.77 | 10.88 | 52.99 | 0.71 |
| % highest blank/ lowest sample | **0.004** | **0.12** | **<0.0000** | **2.06** | **2.93** | **0.08** | **0.69** | **1.64** | **<0.0000** |
| *Run 2* | | | | | | | | | |
| Digestion Blank 1 | <0.00 | 0.54 | <0.00 | 9.14 | <0.00 | 2.27 | 0.00 | 0.48 | 0.00 |
| Digestion Blank 2 | <0.00 | 0.10 | <0.00 | <0.00 | <0.00 | 0.10 | <0.00 | 0.17 | <0.00 |
| Lowest Sample | 6671.18 | 524.20 | 10592.86 | 258.70 | 0.82 | 210.85 | 8.59 | 40.21 | 0.54 |
| % highest blank/ lowest sample | **<0.0000** | **0.10** | **<0.0000** | **3.53** | **<0.0000** | **1.08** | **0.04** | **1.20** | **0.40** |
| *Run 3* | | | | | | | | | |
| Digestion Blank 1 | 8.06 | 0.51 | 3.44 | 6.59 | 0.07 | 2.03 | 0.07 | <0.00 | 0.01 |
| Digestion Blank 2 | 4.85 | 0.15 | <0.00 | 4.68 | 0.02 | 0.78 | 0.02 | <0.00 | 0.00 |
| Lowest Sample | 6732.11 | 516.33 | 10714.39 | 240.06 | 0.99 | 211.74 | 8.35 | 37.68 | 0.55 |
| % highest blank/ lowest sample | **0.12** | **0.10** | **0.03** | **2.74** | **6.55** | **0.96** | **0.85** | **<0.0000** | **1.83** |
| SN  *Run 1* | | | | | | | | | |
| Digestion Blank 1 | <0.00 | 0.02 | <0.00 | 21.78 | 0.01 | 2.13 | <0.00 | <0.00 | <0.00 |
| Digestion Blank 2 | <0.00 | <0.00 | <0.00 | 1.57 | <0.00 | <0.00 | <0.00 | <0.00 | <0.00 |
| Lowest Sample | 5741.90 | 512.64 | 10142.25 | 204.33 | 0.91 | 129.22 | 9.39 | 44.74 | 0.54 |
| % highest blank/ lowest sample | **<0.0000** | **0.004** | **<0.0000** | **10.66** | **1.30** | **1.65** | **<0.0000** | **<0.0000** | **<0.0000** |
| *Run 2* | | | | | | | | | |
| Digestion Blank 1 | <0.00 | <0.00 | <0.00 | <0.00 | <0.00 | 1.57 | <0.00 | <0.00 | <0.00 |
| Digestion Blank 2 | <0.00 | <0.00 | <0.00 | 0.44 | 0.01 | <0.00 | <0.00 | <0.00 | <0.00 |
| Lowest Sample | 5597.36 | 526.85 | 10443.31 | 186.74 | 0.88 | 145.44 | 13.35 | 42.10 | 0.57 |
| % highest blank/ lowest sample | **<0.0000** | **<0.0000** | **<0.0000** | **0.24** | **1.29** | **1.08** | **<0.0000** | **<0.0000** | **<0.0000** |
| *Run 3* | | | | | | | | | |
| Digestion Blank 1 | 4.45 | 0.41 | <0.00 | 1.67 | 0.02 | 7.73 | <0.00 | 0.08 | <0.00 |
| Digestion Blank 2 | <0.00 | <0.00 | <0.00 | 1.99 | <0.00 | 0.77 | <0.00 | <0.00 | <0.00 |
| Lowest Sample | 5998.05 | 467.62 | 9273.86 | 202.80 | 1.10 | 140.07 | 11.98 | 43.05 | 0.57 |
| % highest blank/ lowest sample | **0.07** | **0.09** | **<0.0000** | **0.98** | **2.18** | **5.52** | **<0.0000** | **0.18** | **<0.0000** |
| MCX  *Run 1* | | | | | | | | | |
| Digestion Blank 1 | 3.68 | 3.59 | <0.00 | 1.63 | 0.04 | 12.46 | 1.19 | 0.45 | 0.00 |
| Digestion Blank 2 | 12.71 | 2.77 | 3.36 | 0.02 | <0.00 | 0.76 | <0.00 | <0.00 | <0.00 |
| Lowest Sample | 7080.62 | 408.71 | 8671.25 | 233.71 | 0.87 | 161.32 | 11.95 | 28.73 | 0.57 |
| % highest blank/ lowest sample | **0.18** | **0.88** | **0.04** | **0.70** | **5.08** | **7.73** | **9.95** | **1.55** | **0.67** |
| *Run 2* | | | | | | | | | |
| Digestion Blank 1 | <0.00 | 6.43 | <0.00 | 2.66 | 0.04 | 1.13 | 0.10 | 0.08 | <0.00 |
| Digestion Blank 2 | <0.00 | 11.43 | <0.00 | 10.83 | 0.03 | 1.31 | 0.05 | 0.32 | <0.00 |
| Lowest Sample | 6655.24 | 426.86 | 9776.80 | 218.23 | 0.79 | 179.44 | 10.66 | 38.23 | 0.57 |
| % highest blank/ lowest sample | **<0.0000** | **2.68** | **<0.0000** | **4.96** | **5.12** | **0.73** | **0.98** | **0.85** | **<0.0000** |
| *Run 3* | | | | | | | | | |
| Digestion Blank 1 | 2.02 | 0.21 | <0.00 | <0.00 | 0.06 | 1.41 | 0.32 | <0.00 | 0.002 |
| Digestion Blank 2 | 0.85 | <0.00 | <0.00 | <0.00 | 0.08 | 0.37 | 0.01 | <0.00 | 0.01 |
| Lowest Sample | 6756.12 | 490.97 | 10172.67 | 212.15 | 0.93 | 195.95 | 9.26 | 37.34 | 0.51 |
| % highest blank/ lowest sample | **0.03** | **0.04** | **<0.0000** | **<0.0000** | **8.07** | **0.72** | **3.49** | **<0.0000** | **2.45** |

## **Supplementary Table B2: Detection limit, limit of quantitation and background equivalent concentration for each physiological metal measured in the PDD cohort**

|  | 23 Na (µg/L) | 24 Mg (µg/L) | 39 K (µg/L) | 44 Ca (µg/L) | 55 Mn (µg/L) | 56 Fe (µg/L) | 63 Cu (µg/L) | 66 Zn (µg/L) | 78 Se (µg/L) |
| --- | --- | --- | --- | --- | --- | --- | --- | --- | --- |
| CB  Run 1 | | | | | | | | | |
| DL | 1.25 | 0.19 | 8.79 | 5.11 | 0.02 | 0.33 | 0.01 | 0.06 | 0.02 |
| LOQ | 50 | 50 | 50 | 50 | 0.5 | 50 | 0.5 | 0.5 | 0.5 |
| BEC | 20.19 | 0.39 | 62.23 | 4.95 | 0.08 | 0.32 | 0.05 | 0.11 | 0.02 |
| Lowest Sample | **5794.57** | **587.01** | **10558.52** | **227.17** | **0.91** | **150.67** | **11.31** | **47.03** | **0.55** |
| Run 2 | | | | | | | | | |
| DL | 4.22 | 0.05 | 11.13 | 7.37 | 0.04 | 2.41 | 0.02 | 0.08 | 0.02 |
| LOQ | 50 | 50 | 50 | 50 | 0.5 | 50 | 1 | 1 | 0.5 |
| BEC | 23.02 | 0.55 | 59.81 | 12.89 | 0.07 | 1.22 | 0.08 | 0.37 | 0.02 |
| Lowest Sample | **6255.47** | **438.12** | **8677.22** | **272.52** | **0.74** | **151.70** | **11.52** | **56.50** | **0.46** |
| Run 3 | | | | | | | | | |
| DL | 4.22 | 0.05 | 11.13 | 7.37 | 0.04 | 2.41 | 0.02 | 0.08 | 0.02 |
| LOQ | 50 | 50 | 50 | 50 | 0.5 | 50 | 1 | 1 | 0.5 |
| BEC | 23.02 | 0.55 | 59.81 | 12.89 | 0.07 | 1.22 | 0.08 | 0.37 | 0.02 |
| Lowest Sample | **4195.60** | **464.36** | **8090.52** | **147.02** | **0.67** | **84.20** | **7.20** | **34.27** | **0.46** |
| MTC  Run 1 | | | | | | | | | |
| DL | 1.25 | 0.19 | 8.79 | 5.11 | 0.02 | 0.33 | 0.01 | 0.06 | 0.02 |
| LOQ | 50 | 50 | 50 | 50 | 0.5 | 50 | 0.5 | 0.5 | 0.5 |
| BEC | 20.19 | 0.39 | 62.23 | 4.95 | 0.08 | 0.32 | 0.05 | 0.11 | 0.02 |
| Lowest Sample | **6762.80** | **464.23** | **9400.89** | **288.25** | **0.74** | **171.52** | **10.94** | **54.27** | **0.53** |
| Run 2 | | | | | | | | | |
| DL | 4.22 | 0.05 | 11.13 | 7.37 | 0.04 | 2.41 | 0.02 | 0.08 | 0.02 |
| LOQ | 50 | 50 | 50 | 50 | 0.5 | 50 | 1 | 1 | 0.5 |
| BEC | 23.02 | 0.55 | 59.81 | 12.89 | 0.07 | 1.22 | 0.08 | 0.37 | 0.02 |
| Lowest Sample | **6255.47** | **438.12** | **8677.22** | **272.52** | **0.74** | **151.70** | **11.52** | **56.50** | **0.46** |
| Run 3 | | | | | | | | | |
| DL | 4.22 | 0.05 | 11.13 | 7.37 | 0.04 | 2.41 | 0.02 | 0.08 | 0.02 |
| LOQ | 50 | 50 | 50 | 50 | 0.5 | 50 | 1 | 1 | 0.5 |
| BEC | 23.02 | 0.55 | 59.81 | 12.89 | 0.07 | 1.22 | 0.08 | 0.37 | 0.02 |
| Lowest Sample | **4456.93** | **338.17** | **7045.70** | **188.46** | **0.56** | **142.77** | **7.44** | **39.18** | **0.33** |
| CG  Run 1 | | | | | | | | | |
| DL | 2.36 | 0.51 | 12.91 | 9.29 | 0.04 | 0.18 | 0.05 | 0.18 | 0.04 |
| LOQ | 50 | 50 | 100 | 50 | 0.5 | 50 | 0.5 | 0.5 | 0.5 |
| BEC | 33.85 | 1.09 | 71.37 | 14.59 | 0.09 | 3.77 | 3.80 | 1.04 | 0.05 |
| Lowest Sample | **6601.59** | **502.38** | **9216.55** | **221.33** | **0.84** | **160.95** | **8.63** | **41.76** | **0.47** |
| Run 2 | | | | | | | | | |
| DL | 2.10 | 0.68 | 9.91 | 2.70 | 0.01 | 0.46 | 0.05 | 0.15 | 0.03 |
| LOQ | 400 | 50 | 50 | 50 | 0.5 | 50 | 0.5 | 1 | 0.5 |
| BEC | 61.21 | 1.48 | 78.25 | 12.07 | 0.03 | 1.15 | 0.40 | 0.60 | 0.03 |
| Lowest Sample | **6889.55** | **507.92** | **10353.11** | **247.46** | **0.81** | **161.52** | **10.72** | **40.01** | **0.50** |
| Run 3 | | | | | | | | | |
| DL | 1.60 | 0.52 | 10.49 | 6.72 | 0.02 | 0.31 | 0.01 | 0.03 | 0.02 |
| LOQ | 50 | 100 | 100 | 100 | 0.5 | 50 | 0.5 | 0.5 | 1 |
| BEC | 28.20 | 0.75 | 67.34 | 8.27 | 0.04 | 0.48 | 0.34 | 0.31 | 0.03 |
| Lowest Sample | **6161.79** | **462.58** | **8600.54** | **188.47** | **0.85** | **154.50** | **7.60** | **43.71** | **0.51** |
| HP  Run 1 | | | | | | | | | |
| DL | 1.54 | 0.63 | 9.94 | 7.51 | 0.01 | 0.23 | 0.05 | 0.08 | 0.01 |
| LOQ | 50 | 50 | 100 | 100 | 0.5 | 50 | 0.5 | 1 | 0.5 |
| BEC | 26.36 | 5.40 | 68.45 | 10.29 | 0.03 | 0.69 | 0.18 | 0.38 | 0.03 |
| Lowest Sample | **6064.09** | **424.01** | **8729.82** | **192.02** | **0.88** | **131.60** | **6.18** | **44.49** | **0.49** |
| Run 2 | | | | | | | | | |
| DL | 3.24 | 1.01 | 2.63 | 3.56 | 0.01 | 0.12 | 0.01 | 0.09 | 0.01 |
| LOQ | 100 | 100 | 100 | 100 | 1 | 100 | 1 | 1 | 1 |
| BEC | 24.63 | 5.52 | 68.80 | 7.17 | 0.01 | 0.36 | 0.05 | 0.09 | 0.02 |
| Lowest Sample | **6507.34** | **481.95** | **8864.68** | **249.53** | **0.97** | **143.61** | **8.04** | **55.63** | **0.56** |
| Run 3 | | | | | | | | | |
| DL | 1.59 | 0.27 | 4.56 | 6.11 | 0.02 | 0.08 | 0.01 | 0.16 | 0.01 |
| LOQ | 50 | 50 | 50 | 50 | 0.5 | 50 | 1 | 1 | 0.5 |
| BEC | 21.74 | 0.65 | 58.95 | 12.49 | 0.01 | 0.50 | 0.08 | 0.28 | 0.02 |
| Lowest Sample | **7502.99** | **509.60** | **9530.53** | **246.46** | **1.03** | **165.44** | **7.44** | **57.60** | **0.55** |
| LC  Run 1 | | | | | | | | | |
| DL | 1.54 | 0.63 | 9.94 | 7.51 | 0.01 | 0.23 | 0.05 | 0.08 | 0.01 |
| LOQ | 100 | 50 | 100 | 100 | 0.5 | 50 | 0.5 | 1 | 0.5 |
| BEC | 26.36 | 5.40 | 68.45 | 10.29 | 0.03 | 0.69 | 0.18 | 0.38 | 0.03 |
| Lowest Sample | **5071.31** | **423.98** | **8749.34** | **178.42** | **0.89** | **84.66** | **8.26** | **24.15** | **0.44** |
| Run 2 | | | | | | | | | |
| DL | 3.24 | 1.01 | 2.63 | 3.56 | 0.01 | 0.12 | 0.01 | 0.09 | 0.01 |
| LOQ | 100 | 100 | 100 | 100 | 1 | 100 | 1 | 1 | 1 |
| BEC | 24.63 | 5.52 | 68.80 | 7.17 | 0.01 | 0.36 | 0.05 | 0.09 | 0.02 |
| Lowest Sample | **5475.42** | **373.75** | **7702.90** | **165.35** | **0.90** | **85.96** | **9.56** | **28.36** | **0.41** |
| Run 3 | | | | | | | | | |
| DL | 1.59 | 0.27 | 4.56 | 6.11 | 0.02 | 0.08 | 0.01 | 0.16 | 0.01 |
| LOQ | 50 | 50 | 50 | 50 | 5 | 50 | 1 | 1 | 0.5 |
| BEC | 21.74 | 0.65 | 58.95 | 12.49 | 0.01 | 0.50 | 0.08 | 0.28 | 0.02 |
| Lowest Sample | **5665.10** | **583.71** | **11616.12** | **224.52** | **1.02** | **91.96** | **9.45** | **42.59** | **0.74** |
| MED  Run 1 | | | | | | | | | |
| DL | 4.19 | 0.83 | 14.94 | 9.25 | 0.01 | 0.63 | 0.02 | 0.12 | 0.04 |
| LOQ | 50 | 50 | 50 | 50 | 0.5 | 50 | 1 | 1 | 0.5 |
| BEC | 22.90 | 0.85 | 62.19 | 6.99 | 0.03 | 0.71 | 0.05 | 0.15 | 0.02 |
| Lowest Sample | **6237.57** | **554.45** | **9257.73** | **200.11** | **0.89** | **73.35** | **10.68** | **54.57** | **0.68** |
| Run 2 | | | | | | | | | |
| DL | 4.45 | 0.55 | 9.51 | 2.13 | 0.04 | 0.27 | 0.04 | 0.08 | 0.03 |
| LOQ | 50 | 50 | 50 | 100 | 1 | 50 | 2 | 1 | 1 |
| BEC | 31.33 | 0.66 | 59.31 | 13.54 | 0.09 | 0.97 | 0.16 | 0.26 | 0.03 |
| Lowest Sample | **6295.84** | **574.99** | **9397.09** | **231.75** | **0.86** | **52.25** | **5.78** | **41.49** | **0.61** |
| Run 3 | | | | | | | | | |
| DL | 2.29 | 0.43 | 5.18 | 4.84 | 0.04 | 0.21 | 0.05 | 0.20 | 0.01 |
| LOQ | 50 | 50 | 50 | 50 | 0.5 | 50 | 0.5 | 1 | 0.5 |
| BEC | 24.87 | 0.40 | 63.75 | 5.39 | 0.03 | 0.28 | 0.14 | 0.64 | 0.02 |
| Lowest Sample | **6659.22** | **578.75** | **9598.33** | **263.51** | **0.99** | **72.62** | **11.63** | **48.27** | **0.56** |
| PVC  Run 1 | | | | | | | | | |
| DL | 4.19 | 0.83 | 14.94 | 9.25 | 0.01 | 0.63 | 0.02 | 0.12 | 0.04 |
| LOQ | 50 | 50 | 50 | 50 | 0.5 | 50 | 1 | 1 | 0.5 |
| BEC | 22.90 | 0.85 | 62.19 | 6.99 | 0.03 | 0.71 | 0.05 | 0.15 | 0.02 |
| Lowest Sample | **5571.40** | **497.75** | **11780.77** | **216.17** | **0.80** | **224.77** | **10.88** | **52.99** | **0.71** |
| Run 2 | | | | | | | | | |
| DL | 4.45 | 0.55 | 9.51 | 2.13 | 0.04 | 0.27 | 0.04 | 0.08 | 0.03 |
| LOQ | 50 | 50 | 50 | 100 | 1 | 50 | 1 | 1 | 1 |
| BEC | 31.33 | 0.66 | 59.31 | 13.54 | 0.09 | 0.97 | 0.16 | 0.26 | 0.03 |
| Lowest Sample | **6671.18** | **524.20** | **10592.86** | **258.70** | **0.82** | **210.85** | **8.59** | **40.21** | **0.54** |
| Run 3 | | | | | | | | | |
| DL | 2.29 | 0.43 | 5.18 | 4.84 | 0.04 | 0.21 | 0.05 | 0.20 | 0.01 |
| LOQ | 50 | 50 | 50 | 50 | 0.5 | 50 | 0.5 | 1 | 0.5 |
| BEC | 24.87 | 0.40 | 63.75 | 5.39 | 0.03 | 0.28 | 0.14 | 0.64 | 0.02 |
| Lowest Sample | **6732.11** | **516.33** | **10714.39** | **240.06** | **0.99** | **211.74** | **8.35** | **37.68** | **0.55** |
| SN  Run 1 | | | | | | | | | |
| DL | 2.36 | 0.51 | 12.91 | 9.29 | 0.04 | 0.18 | 0.05 | 0.18 | 0.04 |
| LOQ | 50 | 50 | 100 | 50 | 0.5 | 50 | 0.5 | 0.5 | 0.5 |
| BEC | 33.85 | 1.09 | 71.37 | 14.59 | 0.09 | 3.77 | 3.80 | 1.04 | 0.05 |
| Lowest Sample | **5741.90** | **512.64** | **10142.25** | **204.33** | **0.91** | **129.22** | **9.39** | **44.74** | **0.54** |
| Run 2 | | | | | | | | | |
| DL | 2.10 | 0.68 | 9.91 | 2.70 | 0.01 | 0.46 | 0.05 | 0.15 | 0.03 |
| LOQ | 400 | 50 | 100 | 50 | 0.5 | 50 | 1 | 1 | 1 |
| BEC | 61.21 | 1.48 | 78.25 | 12.07 | 0.03 | 1.15 | 0.40 | 0.60 | 0.03 |
| Lowest Sample | **5597.36** | **526.85** | **10443.31** | **186.74** | **0.88** | **145.44** | **13.35** | **42.10** | **0.57** |
| Run 3 | | | | | | | | | |
| DL | 1.60 | 0.52 | 10.49 | 6.72 | 0.02 | 0.31 | 0.01 | 0.03 | 0.02 |
| LOQ | 50 | 100 | 100 | 100 | 0.5 | 50 | 0.5 | 0.5 | 1 |
| BEC | 28.20 | 0.75 | 67.34 | 8.27 | 0.04 | 0.48 | 0.34 | 0.31 | 0.03 |
| Lowest Sample | **5998.05** | **467.62** | **9273.86** | **202.80** | **1.10** | **140.07** | **11.98** | **43.05** | **0.57** |
| MCX  Run 1 | | | | | | | | | |
| DL | 3.14 | 1.91 | 8.81 | 10.67 | 0.02 | 0.33 | 0.01 | 0.13 | 0.02 |
| LOQ | 50 | 50 | 50 | 100 | 0.5 | 50 | 0.5 | 1 | 0.5 |
| BEC | 56.27 | 7.75 | 56.63 | 12.12 | 0.05 | 0.60 | 0.09 | 0.55 | 0.02 |
| Lowest Sample | **7080.62** | **408.71** | **8671.25** | **233.71** | **0.87** | **161.32** | **11.95** | **28.73** | **0.57** |
| Run 2 | | | | | | | | | |
| DL | 7.98 | 0.50 | 7.80 | 5.15 | 0.02 | 0.09 | 0.003 | 0.03 | 0.01 |
| LOQ | 200 | 100 | 200 | 100 | 1 | 100 | 1 | 1 | 1 |
| BEC | 61.80 | 4.60 | 61.70 | 5.51 | 0.04 | 0.53 | 0.04 | 0.27 | 0.02 |
| Lowest Sample | **6655.24** | **426.86** | **9776.80** | **218.23** | **0.79** | **179.44** | **10.66** | **38.23** | **0.57** |
| Run 3 | | | | | | | | | |
| DL | 3.39 | 0.42 | 5.30 | 14.35 | 0.01 | 0.31 | 0.06 | 0.18 | 1.00 |
| LOQ | 50 | 50 | 50 | 50 | 0.5 | 50 | 1 | 0.5 | 0.5 |
| BEC | 46.64 | 0.87 | 65.54 | 15.81 | 0.01 | 0.62 | 0.26 | 1.77 | 0.01 |
| Lowest Sample | **6756.12** | **490.97** | **10172.67** | **212.15** | **0.93** | **195.95** | **9.26** | **37.34** | **0.51** |

## **Supplementary Figure B1: Standard curves for each physiological metal measured in the PDD cohort**

**MTC + CB 1**


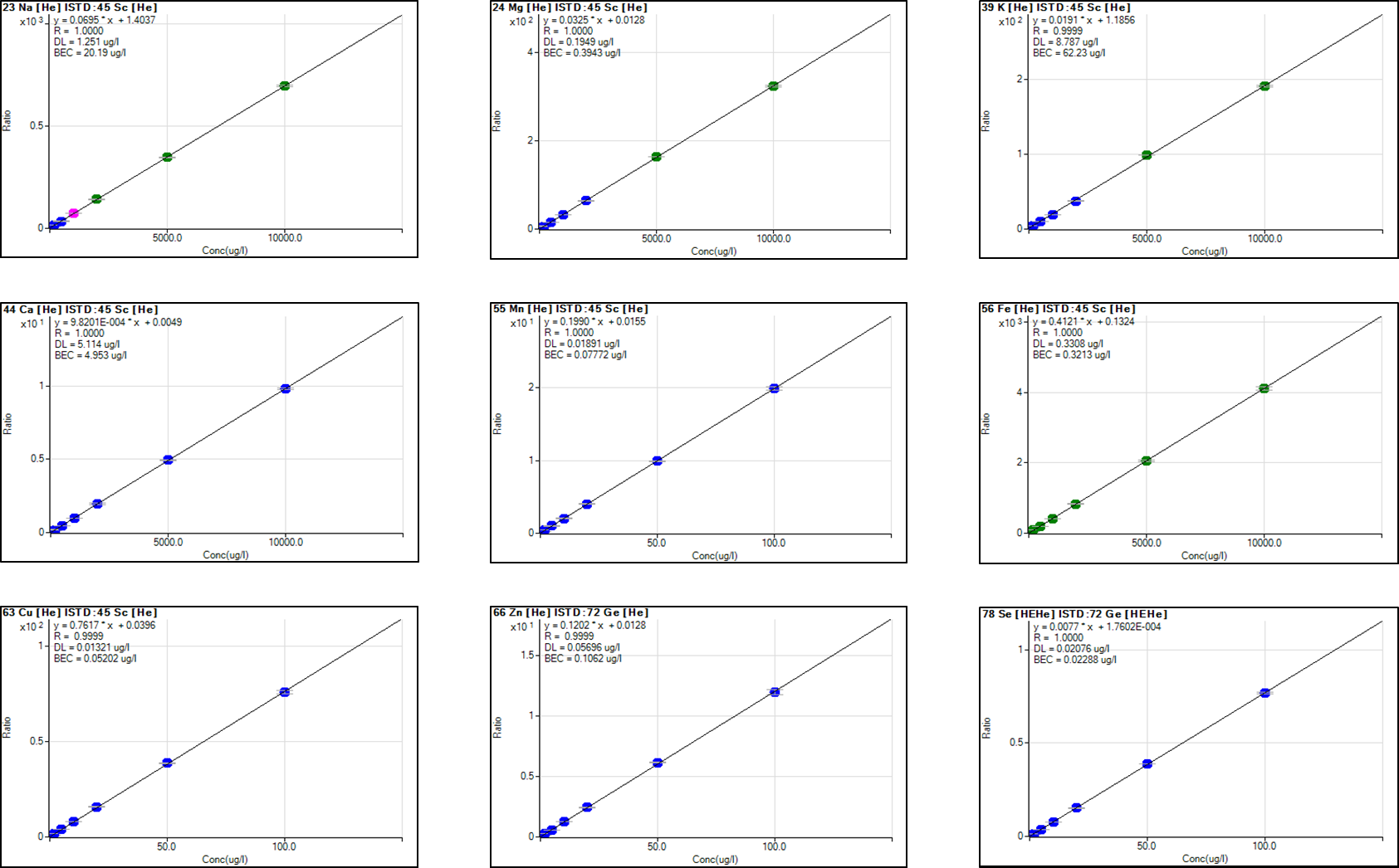


**MTC + CB 2**


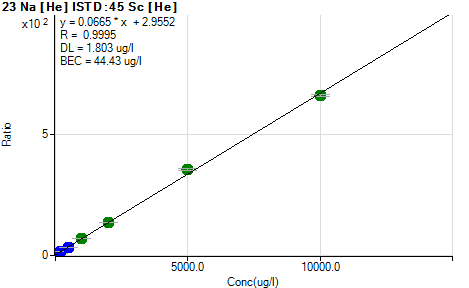

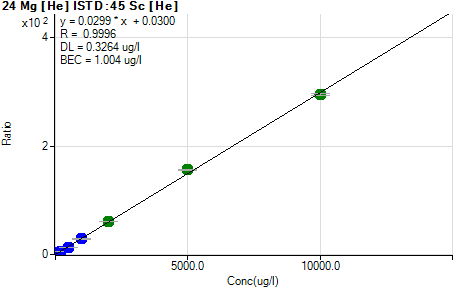

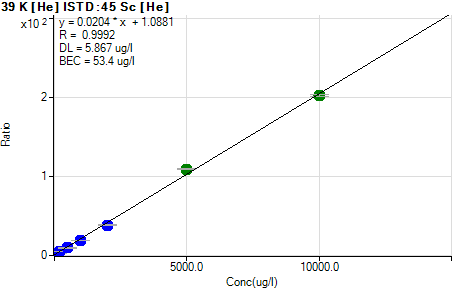

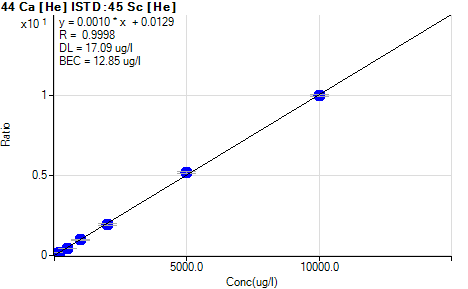

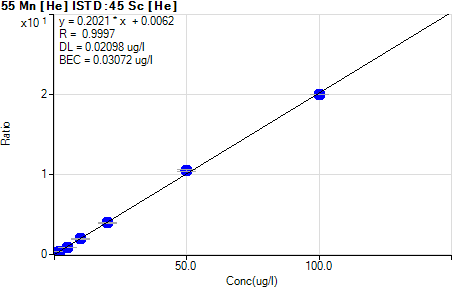

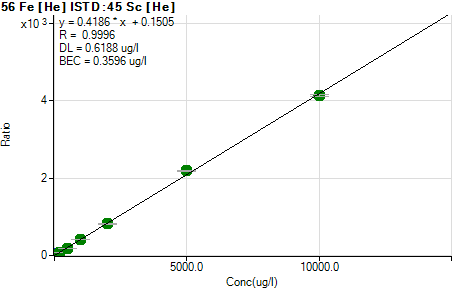

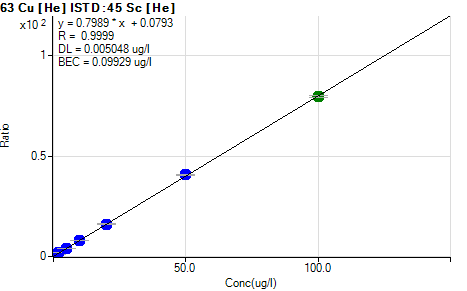

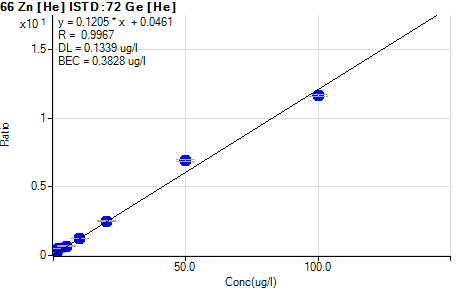

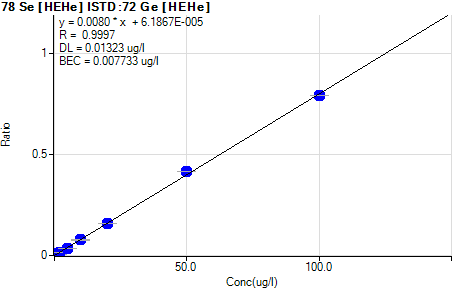

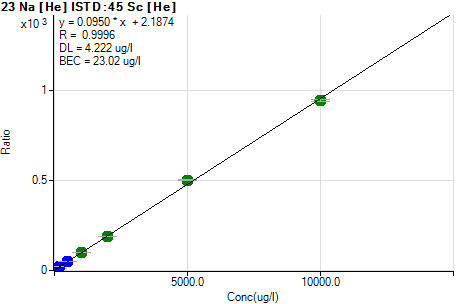

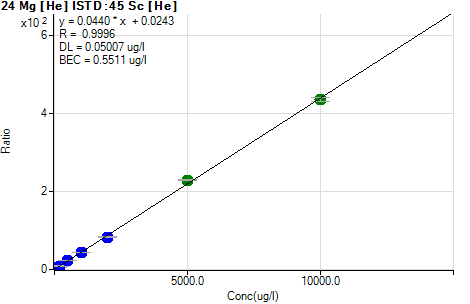

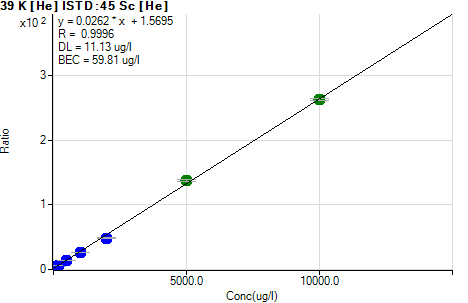

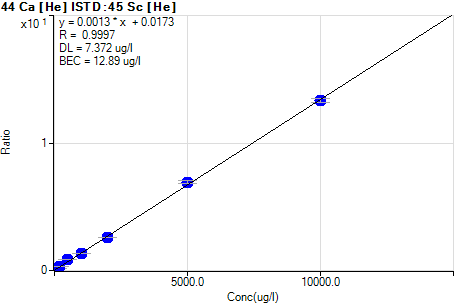

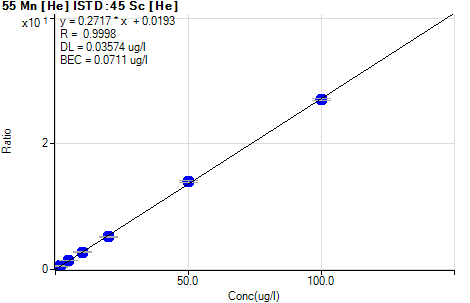

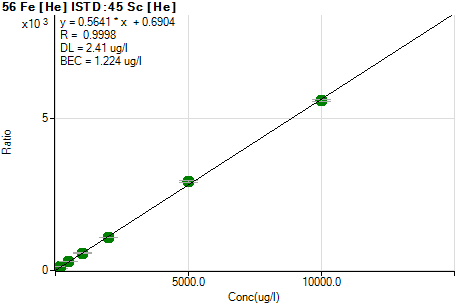

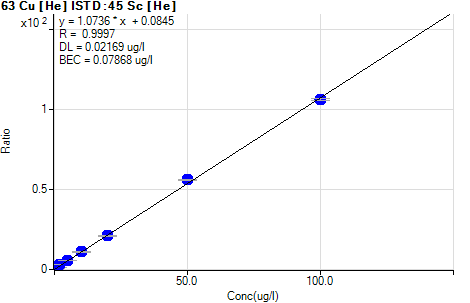

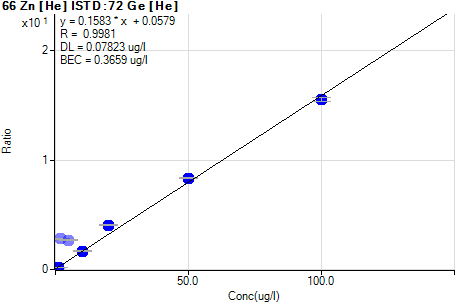

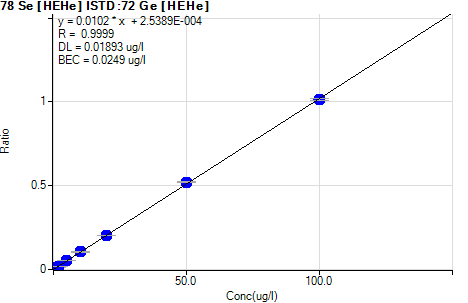


**LC + HP 1**


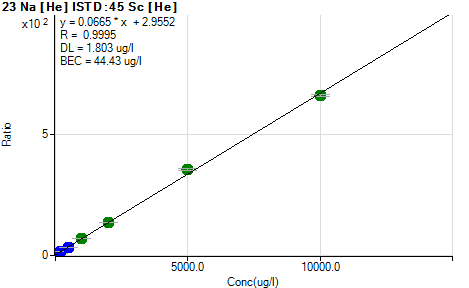

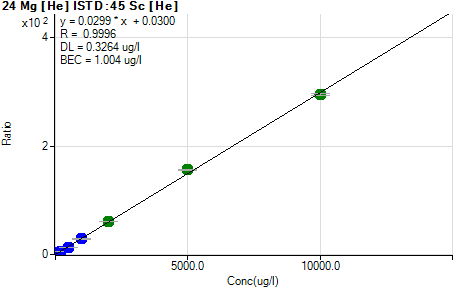

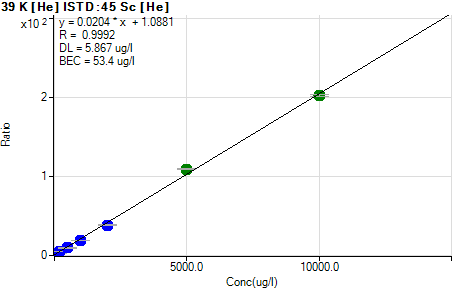

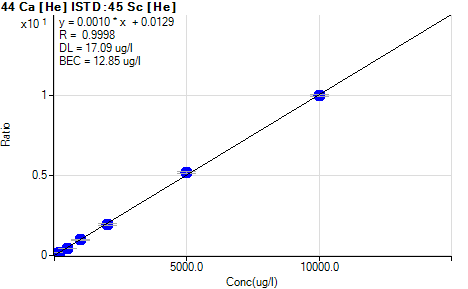

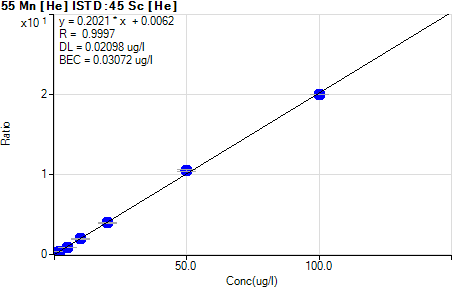

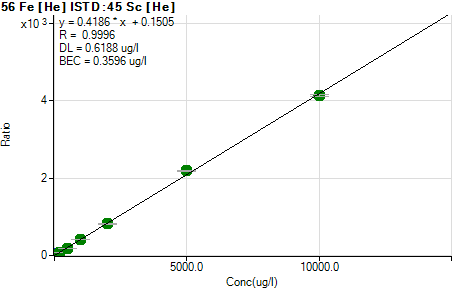

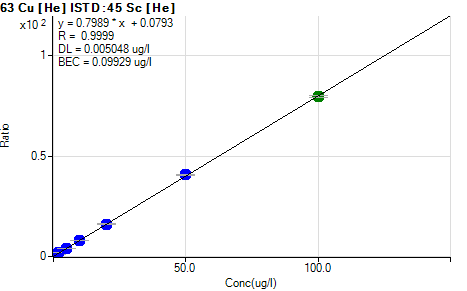

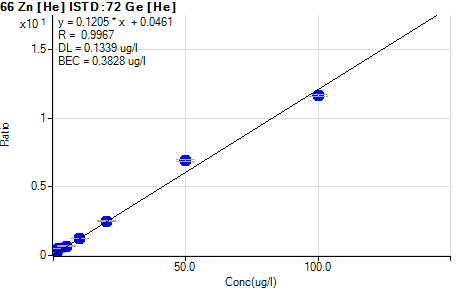

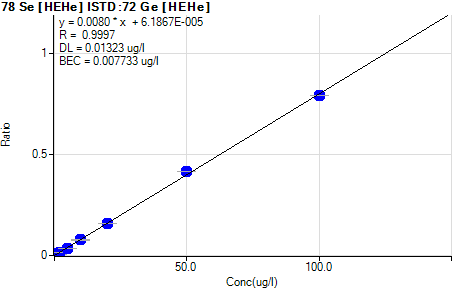

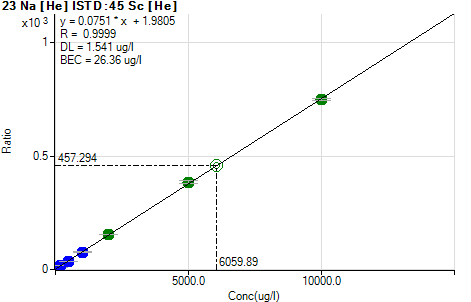

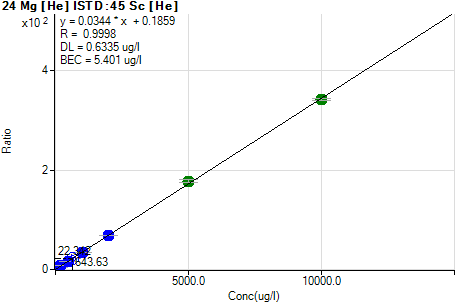

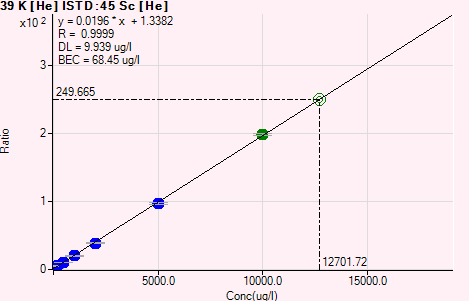

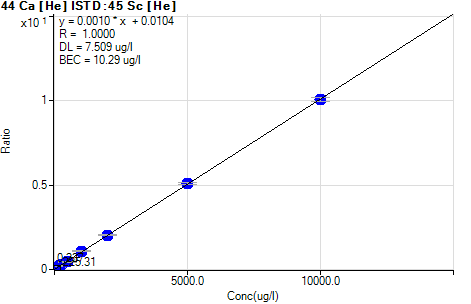

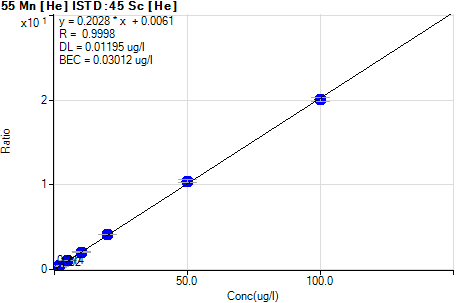

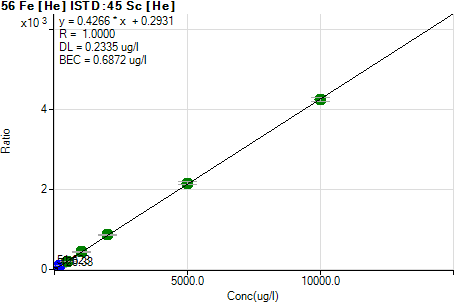

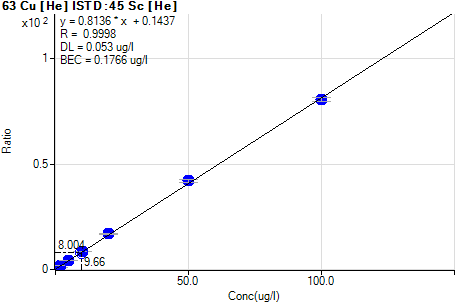

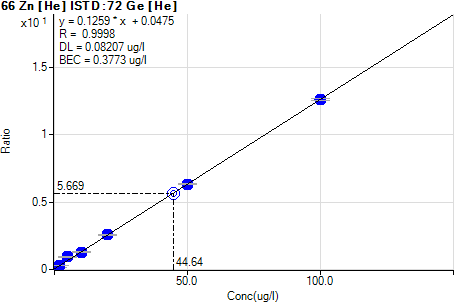

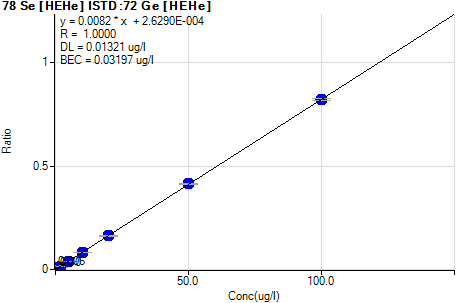


**LC + HP 2**


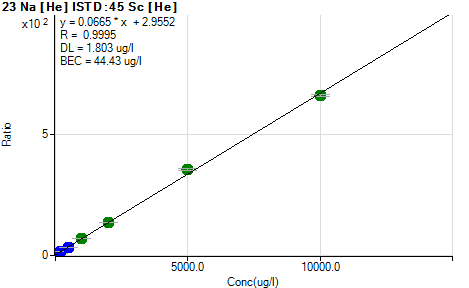

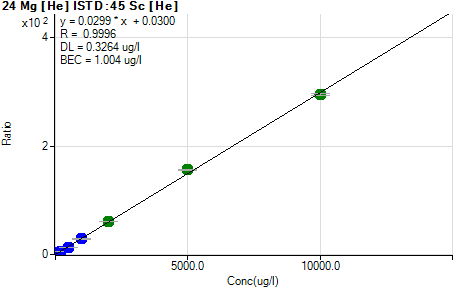

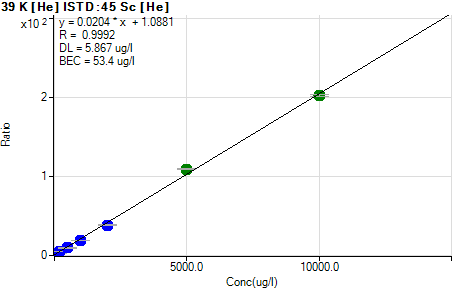

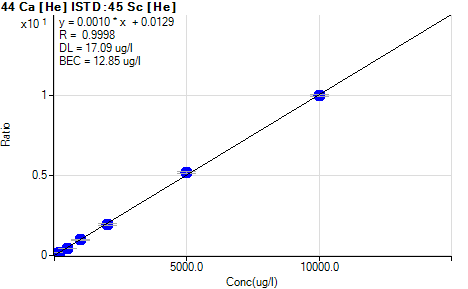

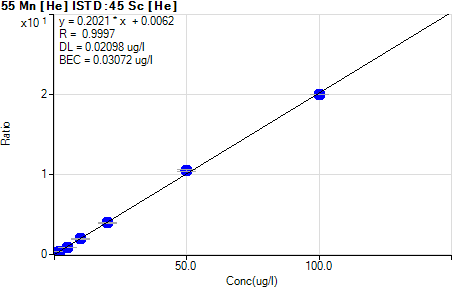

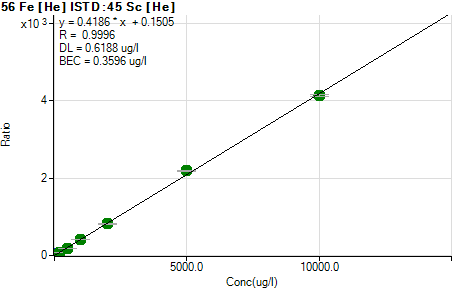

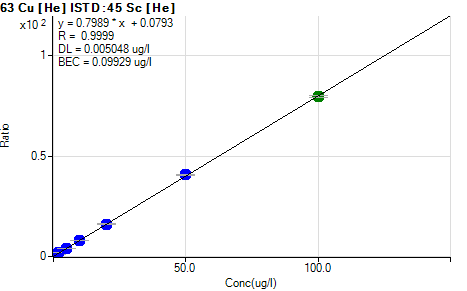

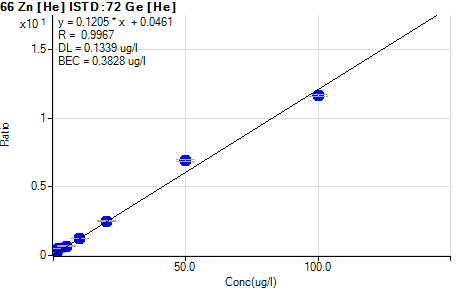

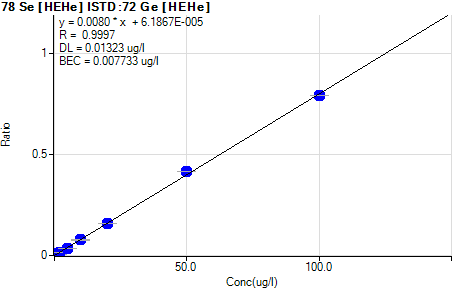

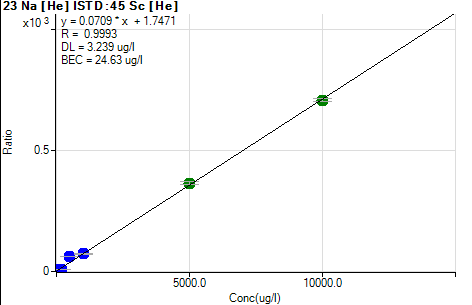

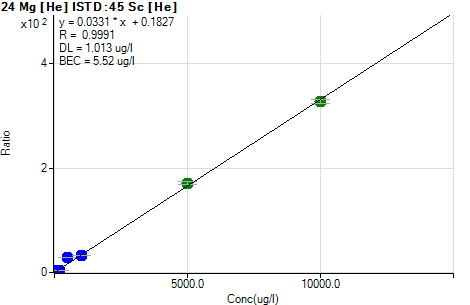

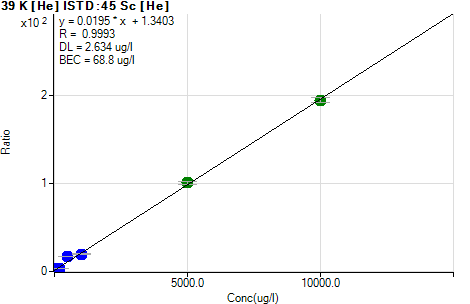

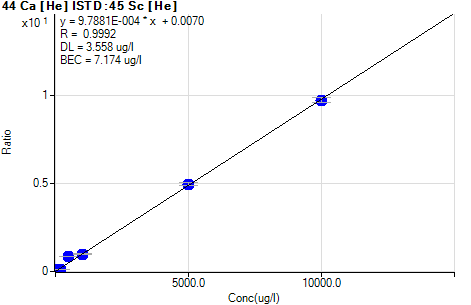

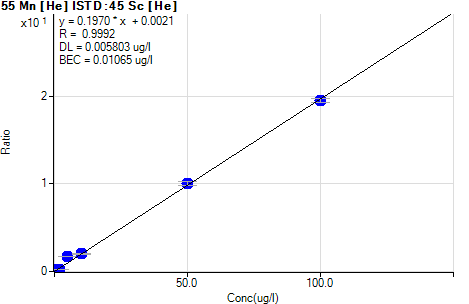

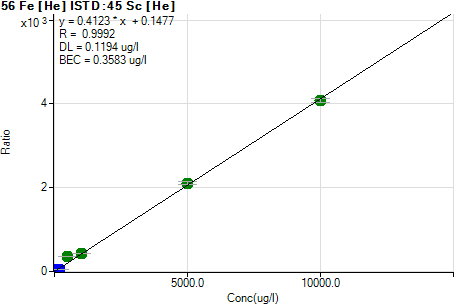

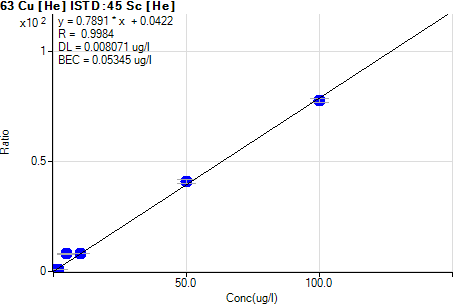

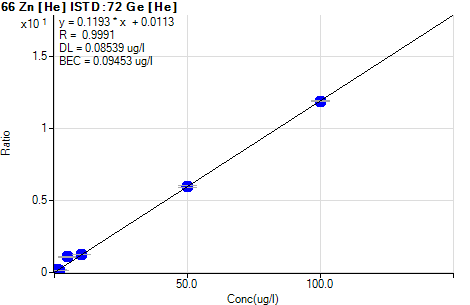

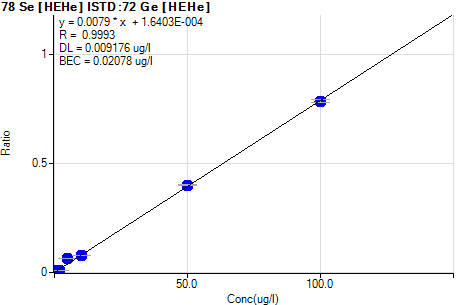


**LC + HP 3**


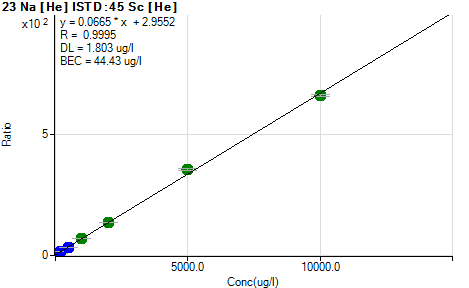

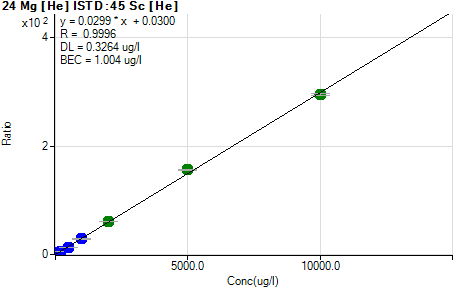

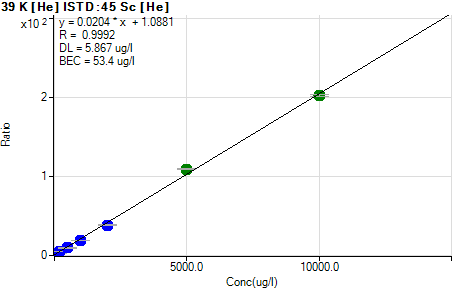

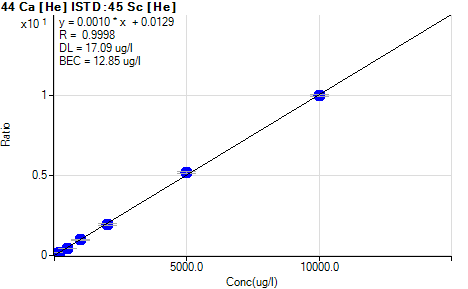

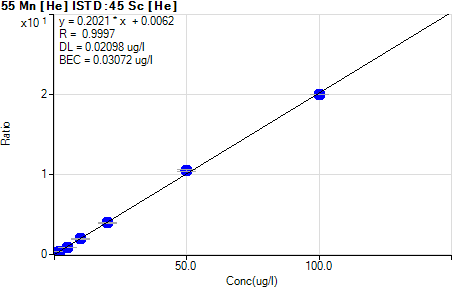

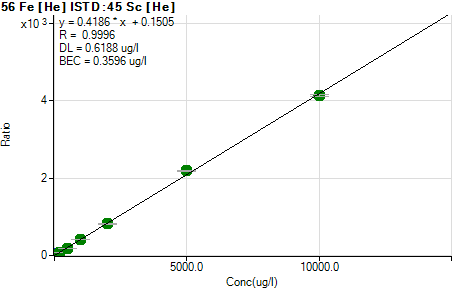

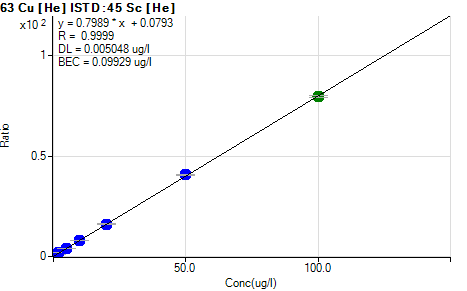

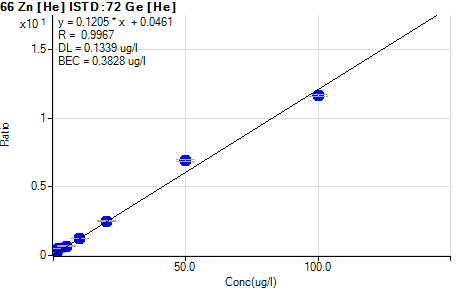

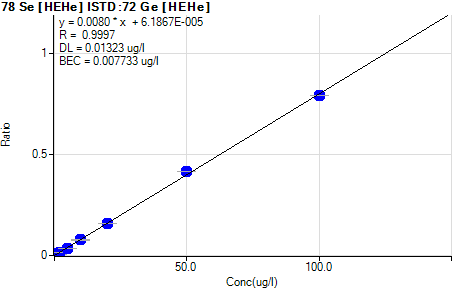

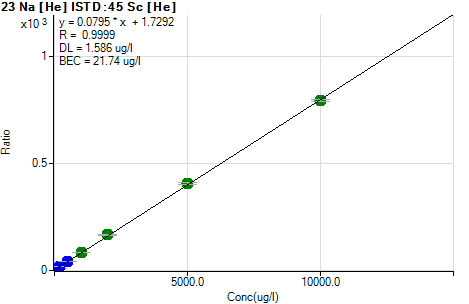

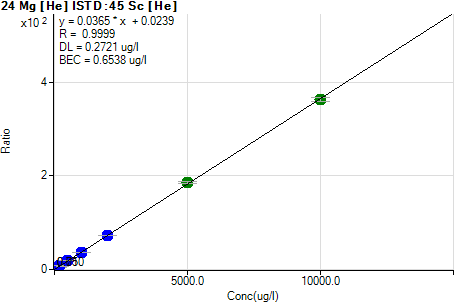

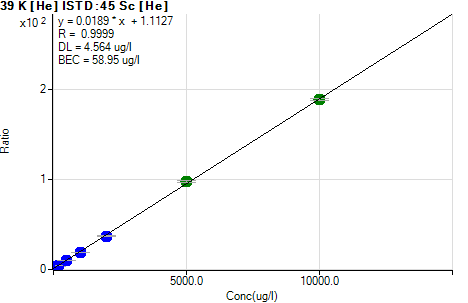

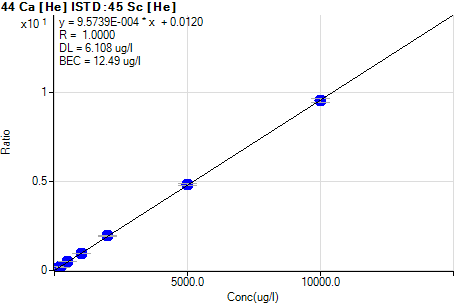

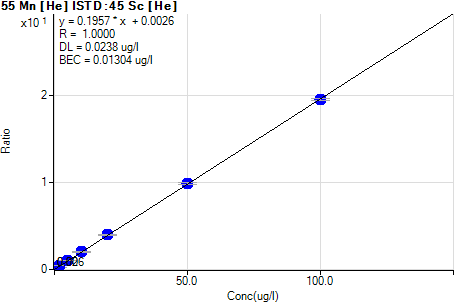

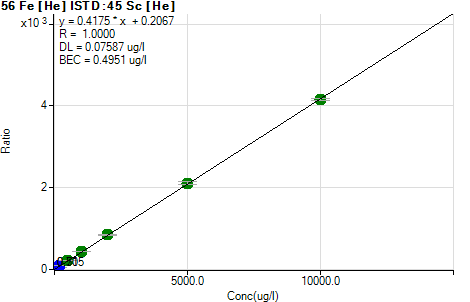

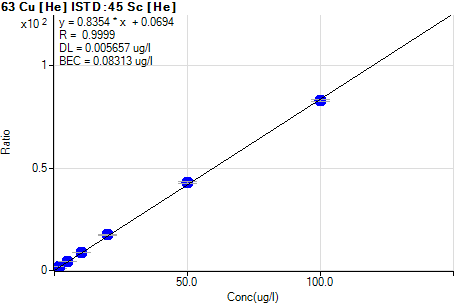

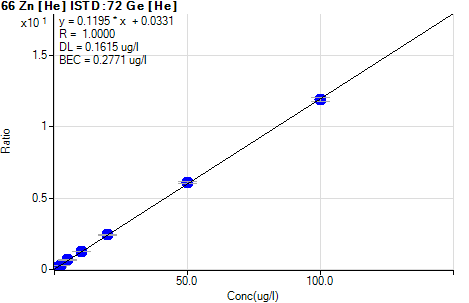

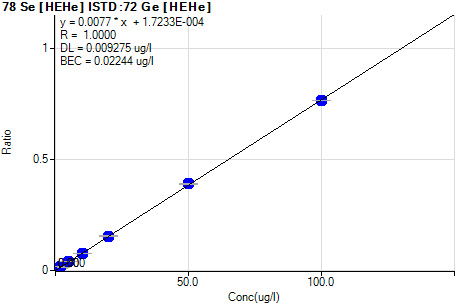


**MTC + PVC 1**


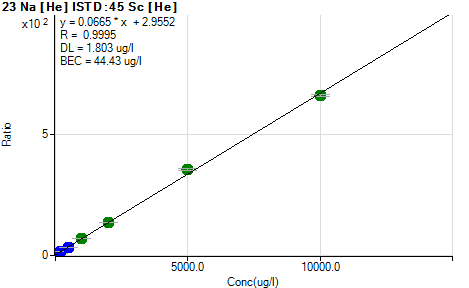

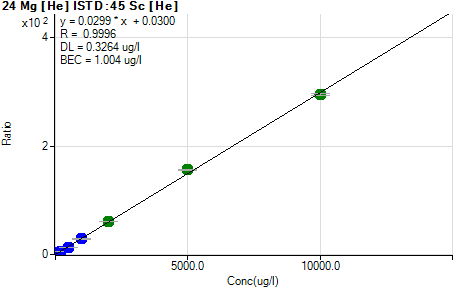

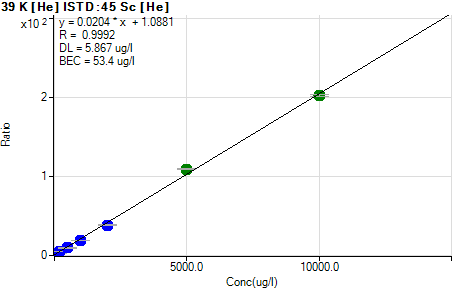

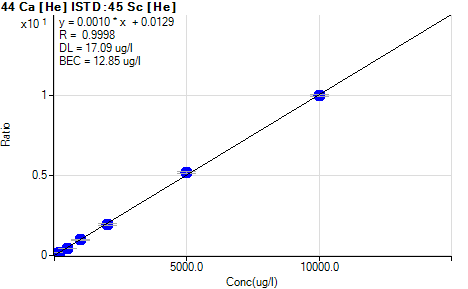

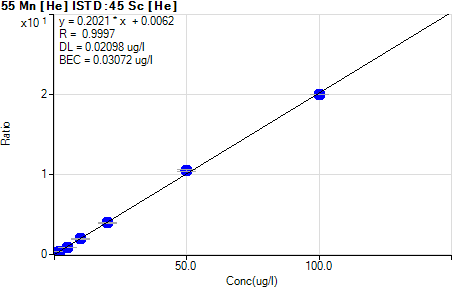

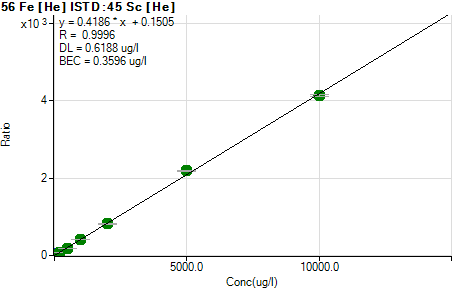

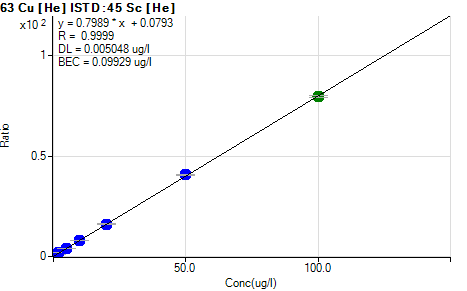

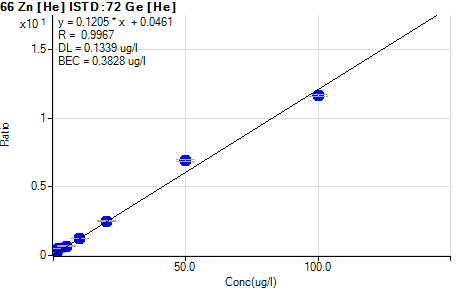

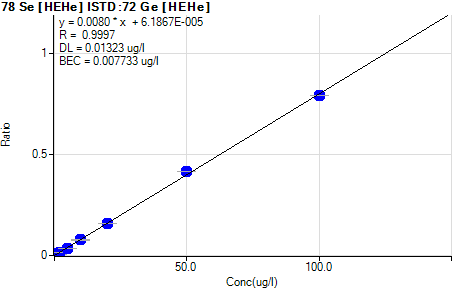

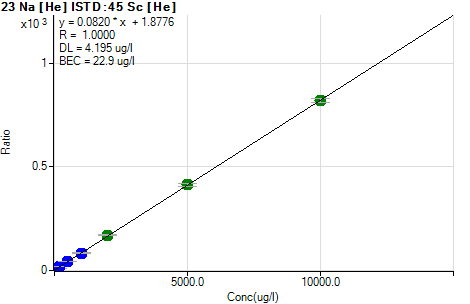

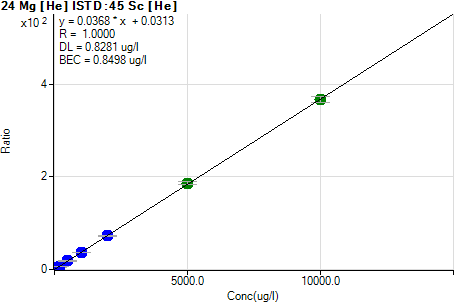

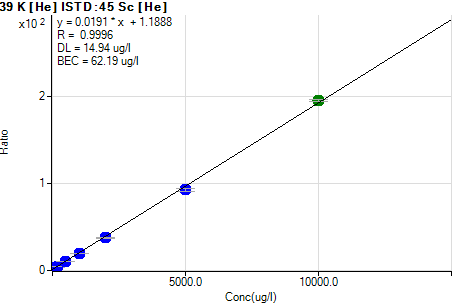

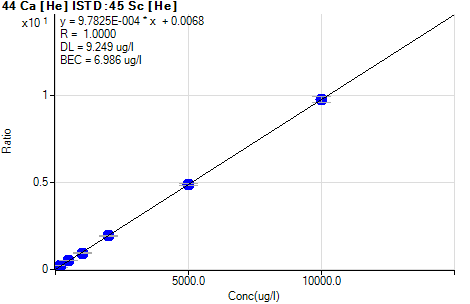

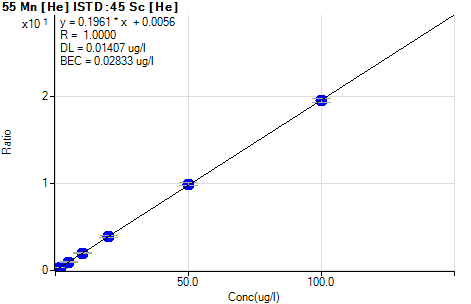

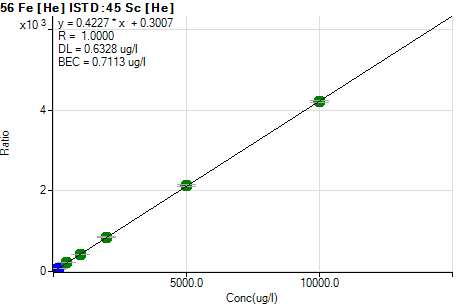

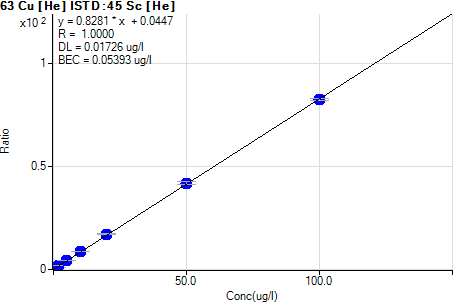

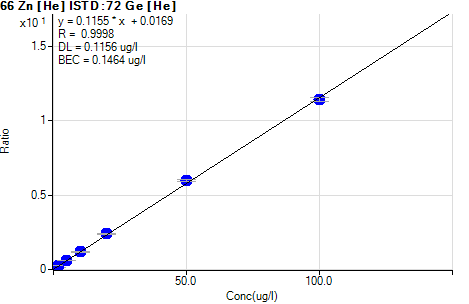

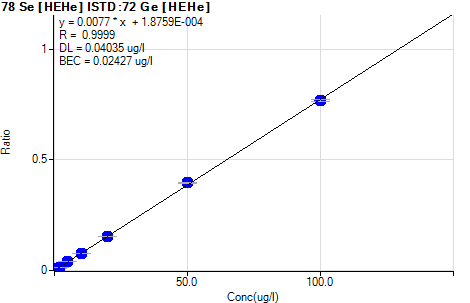


**MED + PVC 2**


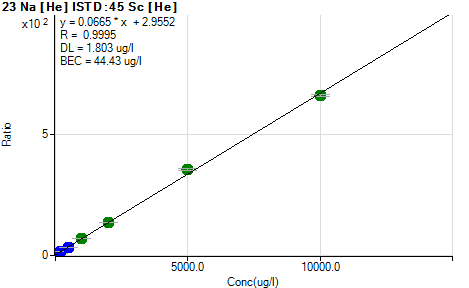

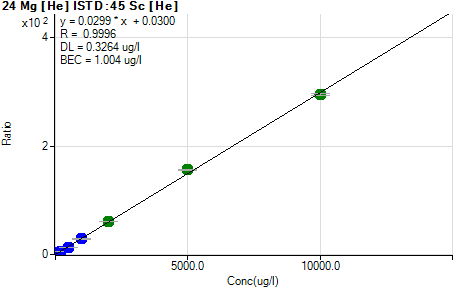

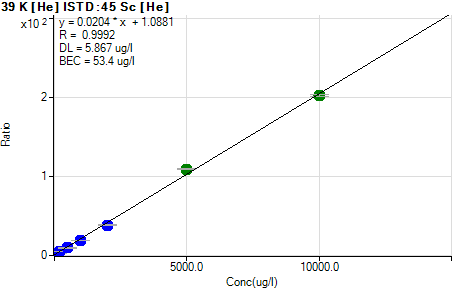

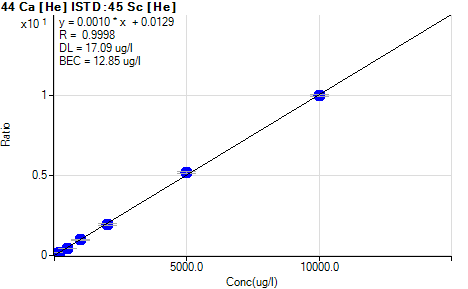

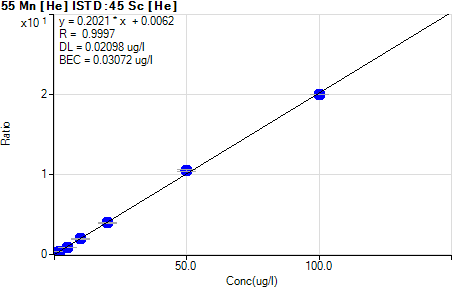

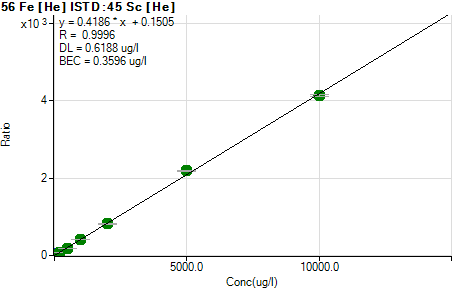

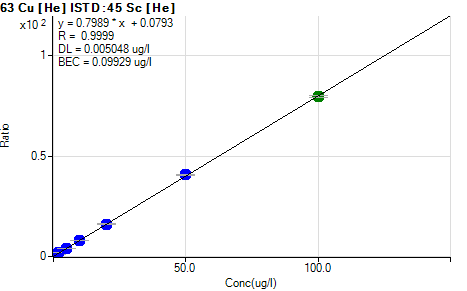

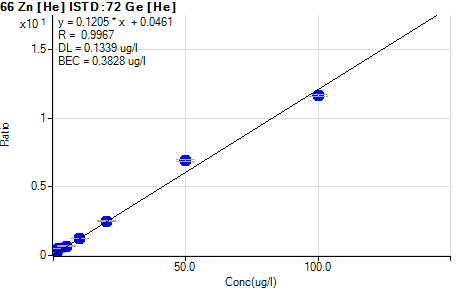

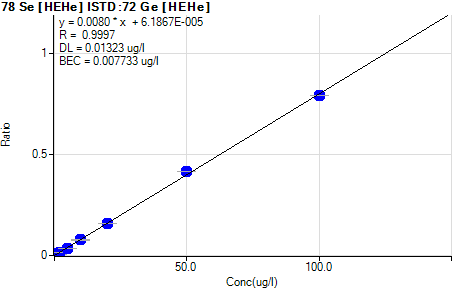


**MED + PVC 3**

**CG + SN 1**

**CG + SN 2**

**CG + SN 3**

**MCX 1**

**MCX 2**

**MCX 3**

## **Supplementary Table B3: Metal concentrations in digestion blanks in Manchester & Newcastle cohorts**

| Run | 23 Na (µg/L) | 24 Mg (µg/L) | 39 K (µg/L) | 44 Ca (µg/L) | 55 Mn (µg/L) | 56 Fe (µg/L) | 63 Cu (µg/L) | 66 Zn (µg/L) | 78 Se (µg/L) |
| --- | --- | --- | --- | --- | --- | --- | --- | --- | --- |
| Manchester Cohort  *Run 1* | | | | | | | | | |
| Digestion Blank 1 | <0.00 | 0.37 | 0.34 | <0.00 | 0.02 | 1.18 | 0.37 | 0.27 | 0.01 |
| Digestion Blank 2 | <0.00 | 0.69 | 11.05 | 1.23 | 0.03 | 1.47 | 0.38 | 0.90 | 0.001 |
| Lowest Sample | 6304.68 | 352.33 | 5770.44 | 212.75 | 0.66 | 76.76 | 5.93 | 42.14 | 0.42 |
| % highest blank/ lowest sample | **<0.0000** | **0.20** | **0.19** | **0.58** | **4.37** | **1.91** | **6.47** | **2.14** | **1.21** |
| *Run 2* | | | | | | | | | |
| Digestion Blank 1 | 184.40 | 0.87 | 6.06 | 9.18 | 0.04 | 1.47 | 0.61 | 0.73 | 0.003 |
| Digestion Blank 2 | <0.00 | 0.36 | 2.48 | 5.02 | 0.02 | 0.69 | 0.27 | 0.71 | <0.00 |
| Lowest Sample | 6127.83 | 317.39 | 5920.04 | 206.36 | 0.89 | 126.80 | 5.12 | 24.85 | 0.19 |
| % highest blank/ lowest sample | **3.01** | **0.27** | **0.10** | **4.45** | **4.48** | **1.16** | **11.89** | **2.94** | **1.77** |
| *Run 3* | | | | | | | | | |
| Digestion Blank 1 | 2.06 | <0.00 | 0.36 | <0.00 | 0.02 | 0.59 | 0.21 | 0.66 | <0.00 |
| Digestion Blank 2 | 6.23 | 1.65 | 0.42 | <0.00 | 0.03 | 2.87 | 0.44 | 0.56 | <0.00 |
| Lowest Sample | 7208.63 | 405.63 | 7457.42 | 172.51 | 0.74 | 99.45 | 4.13 | 24.70 | 0.24 |
| % highest blank/ lowest sample | **0.09** | **0.41** | **0.006** | **<0.0000** | **4.24** | **2.89** | **10.56** | **2.68** | **<0.0000** |
| Newcastle Cohort  *Run 1* | | | | | | | | | |
| Digestion Blank 1 | 7.95 | 0.24 | 3.24 | 13.65 | 0.09 | 2.49 | 0.35 | 2.00 | 0.007 |
| Digestion Blank 2 | 13.17 | 0.24 | 3.06 | 6.83 | 0.15 | 2.58 | 0.56 | 0.72 | <0.00 |
| Lowest Sample | 6205.78 | 430.66 | 7115.22 | 216.40 | 0.85 | 148.49 | 3.62 | 32.93 | 0.49 |
| % highest blank/ lowest sample | **0.21** | **0.06** | **0.05** | **6.31** | **17.40** | **1.74** | **15.52** | **6.09** | **1.50** |
| Auckland AD Cohort  *Batch 1* | | | | | | | | | |
| Digestion Blank 1 | 6.38 | 0.80 | 23.5 | 3.58 | 0.04 | 0.02 | <0.0000 | <0.0000 | <0.0000 |
| Digestion Blank 2 | 10.9 | 3.11 | 24.9 | 11.7 | 0.04 | 1.43 | <0.0000 | 0.35 | <0.0000 |
| Lowest Sample | 6052 | 523 | 8982 | 190 | 0.89 | 201 | 7.57 | 42.0 | 0.66 |
| % highest blank/ lowest sample | **0.18** | **0.59** | **0.28** | **6.15** | **4.44** | **0.71** | **<0.0000** | **0.84** | **<0.0000** |
| *Batch 2* | | | | | | | | | |
| Digestion Blank 1 | 22.7 | 3.13 | 3.87 | 11.5 | 0.03 | 0.61 | 0.18 | 0.25 | 0.01 |
| Digestion Blank 2 | 7.36 | 0.41 | 5.21 | 4.04 | 0.01 | 0.15 | 0.03 | 0.02 | 0.01 |
| Lowest Sample | 4146 | 441 | 6552 | 191 | 0.91 | 154 | 5.91 | 45.0 | 0.59 |
| % highest blank/ lowest sample | **0.55** | **0.71** | **0.008** | **6.00** | **3.00** | **0.40** | **3.10** | **0.55** | **1.64** |
| *Batch 3* | | | | | | | | | |
| Digestion Blank 1 | 11.6 | 1.91 | 12.7 | 4.94 | 0.02 | 0.46 | 0.0002 | 0.03 | 0.01 |
| Digestion Blank 2 | 12.3 | 2.23 | 6.67 | 3.66 | 0.01 | 0.52 | 0.02 | 0.12 | 0.01 |
| Lowest Sample | 6348 | 497 | 8631 | 207 | 0.99 | 118 | 5.33 | 38.4 | 0.55 |
| % highest blank/ lowest sample | **0.19** | **0.45** | **0.15** | **2.39** | **2.26** | **0.44** | **0.44** | **0.31** | **2.68** |

<0.000 indicates that concentration was lower than that of the calibration blank. The Manchester cohort was analysed across three runs, whilst only one run was performed for the Newcastle and Auckland cohorts. Each run included two digestion blanks, as well as tubes containing nitric acid and standards but not samples in order to determine background metal levels. The highest concentration in a blank compared to the lowest concentration in a sample was for Mn in the Newcastle cohort at 17.4%. In all but five incidences, blank concentrations were <5 % of the lowest sample concentration for all metals. In the Manchester cohort, the lowest Cu level was in sample 0919 in each run. Notably, this sample had a Cu concentration almost 4-fold lower than the average (19.4 ug/L). However, even with this unusually low Cu concentration, blank concentrations were still ~10-fold lower. This is similar to the Newcastle cohort, where the lowest Cu concentration was ~5-fold lower than the average (13.8 ug/L). Blanks data for the Auckland HD cohort was not available.

## **Supplementary Table B4: Detection limit, limit of quantitation and background equivalent concentration for each physiological metal measured in the additional cohorts**

|  | 23 Na (µg/L) | 24 Mg (µg/L) | 39 K (µg/L) | 44 Ca (µg/L) | 55 Mn (µg/L) | 56 Fe (µg/L) | 63 Cu (µg/L) | 66 Zn (µg/L) | 78 Se (µg/L) |
| --- | --- | --- | --- | --- | --- | --- | --- | --- | --- |
| Lowest Standard | 50 | 50 | 50 | 50 | 0.5 | 50 | 0.5 | 0.5 | 0.5 |
| Manchester Cohort  *Run 1* | | | | | | | | | |
| DL | 3.47 | 0.44 | 6.04 | 12.79 | 0.02 | 0.39 | 0.01 | 0.06 | 0.01 |
| LOQ | 50 | 50 | 50 | 100 | 0.5 | 50 | 2 | 4 | 0.5 |
| BEC | 124.54 | 0.42 | 51.56 | 8.31 | 0.02 | 0.46 | 0.07 | 0.57 | 0.02 |
| Lowest Sample | 6304.68 | 352.33 | 5770.44 | 212.75 | 0.66 | 76.76 | 5.93 | 42.14 | 0.42 |
| *Run 2* | | | | | | | | | |
| DL | 3.08 | 0.12 | 2.74 | 8.36 | 0.01 | 0.17 | 0.02 | 0.21 | 0.01 |
| LOQ | 100 | 100 | 100 | 100 | 1 | 100 | 0.5 | 4 | 1 |
| BEC | 59.81 | 0.42 | 46.02 | 6.86 | 0.01 | 0.24 | 0.09 | 0.41 | 0.01 |
| Lowest Sample | 6127.83 | 317.39 | 5920.04 | 206.36 | 0.89 | 126.80 | 5.12 | 24.85 | 0.19 |
| *Run 3* | | | | | | | | | |
| DL | 2.64 | 0.45 | 2.58 | 6.25 | 0.01 | 0.08 | 0.02 | 0.13 | 0.02 |
| LOQ | 50 | 50 | 100 | 400 | 0.5 | 50 | 0.5 | 4 | 1 |
| BEC | 29.28 | 0.53 | 40.73 | 73.01 | 0.00 | 0.20 | 0.06 | 0.13 | 0.02 |
| Lowest Sample | 7208.63 | 405.63 | 7457.42 | 172.51 | 0.74 | 99.45 | 4.13 | 24.70 | 0.24 |
| Newcastle Cohort  *Run 1* | | | | | | | | | |
| DL | 1.80 | 0.33 | 5.87 | 17.09 | 0.02 | 0.62 | 0.01 | 0.13 | 0.01 |
| LOQ | 50 | 50 | 100 | 50 | 0.5 | 50 | 1 | 1 | 1 |
| BEC | 44.43 | 1.00 | 53.40 | 12.85 | 0.03 | 0.36 | 0.10 | 0.38 | 0.01 |
| Lowest Sample | 6205.78 | 430.66 | 7115.22 | 216.40 | 0.85 | 148.49 | 3.62 | 32.93 | 0.49 |
| Auckland Cohort  *Batch 1* | | | | | | | | | |
| DL | 13.4 | 0.62 | 2.84 | 7.35 | 0.02 | 0.35 | 0.26 | 0.09 | 0.04 |
| LOQ | 100 | 50 | 200 | 100 | 0.5 | 50 | 2 | 1 | 0.5 |
| BEC | 22.3 | 3.39 | 58.0 | 9.56 | 0.02 | 0.59 | 0.49 | 0.27 | 0.02 |
| Lowest Sample | 6052 | 523 | 8982 | 190 | 0.89 | 201 | 7.57 | 42.0 | 0.66 |
| *Batch 2* | | | | | | | | | |
| DL | 0.94 | 0.55 | 6.04 | 10.60 | 0.006 | 0.16 | 0.02 | 0.06 | 0.007 |
| LOQ | 50 | 50 | 200 | 100 | 0.5 | 50 | 0.5 | 2 | 0.5 |
| BEC | 18.3 | 2.57 | 58.4 | 5.62 | 0.01 | 0.20 | 0.05 | 0.11 | 0.01 |
| Lowest Sample | 4146 | 441 | 6552 | 191 | 0.91 | 154 | 5.91 | 45.0 | 0.59 |
| *Batch 3* | | | | | | | | | |
| DL | 2.20 | 0.30 | 4.17 | 8.11 | 0.02 | 0.27 | 0.004 | 0.08 | 0.02 |
| LOQ | 100 | 50 | 200 | 100 | 0.5 | 50 | 0.5 | 1 | 0.5 |
| BEC | 15.7 | 2.88 | 59.90 | 6.02 | 0.02 | 0.06 | 0.09 | 0.17 | 0.01 |
| Lowest Sample | 6348 | 497 | 8631 | 207 | 0.99 | 118 | 5.33 | 38.4 | 0.55 |

Lowest calibration standards analysed were 50µg/L for Na, Mg, K, Ca and Fe and 0.5µg/L for Mn, Cu, Zn, and Se. The software employed (Mass Hunter, Agilent) automatically calculated values for DLs (detection limits) and BECs (background equivalent concentrations) corresponding to each element analysed. LOQs (limits of quantitation) were calculated by comparison of calibration blanks and standards. Value shown for lowest sample are lowest raw measurements without correction for corresponding tissue mass. The lowest sample level of Se was lower than the LOQ in each run; however it remained well above the DL and so was retained for analysis.

## **Supplementary Figure B2: Standard curves for each physiological metal measured in the additional cohorts**

**Manchester Cohort – Run 1**

**Manchester Cohort – Run 2**

**Manchester Cohort – Run 3**

**Newcastle Cohort – Run 1**

**Auckland Cohort AD – Batch 1**

**Auckland Cohort AD – Batch 2**

**Auckland AD Cohort – Batch 3**

Standard curves were not available for the Auckland HD cohort.
