# Supplementary material for: Widespread Decreases in Cerebral Copper Are Common to Parkinson's Disease Dementia and Alzheimer's Disease Dementia
Source: Front Aging Neurosci. 2021 Mar 3;13:641222. doi: 10.3389/fnagi.2021.641222 (PMC7966713; doi:10.3389/fnagi.2021.641222)
Supplement: Supplementary file 1 [file Data_Sheet_1.zip › PDD Paper - Supplementary Material C (PCA & PLS-DA Plots).docx]

Supplementary Data: Comparisons among Cohort Controls

**Contents**

[**Cerebellum: PDD v AD & HD Cohort Controls** 2](#_Toc31979497)

[**Graphs:** 3](#_Toc31979498)

[**Cingulate Gyrus: PDD v AD Cohort Controls** 4](#_Toc31979499)

[**Graphs:** 5](#_Toc31979500)

[**Hippocampus: PDD v AD & HD Cohort Controls** 6](#_Toc31979501)

[**Graphs:** 7](#_Toc31979502)

[**Middle Temporal Cortex: PDD v AD & HD Cohort Controls** 8](#_Toc31979503)

[**Graphs:** 9](#_Toc31979504)

[**Substantia Nigra: PDD v HD Cohort Controls** 10](#_Toc31979505)

[**Graphs:** 11](#_Toc31979506)

[**Motor Cortex: PDD v AD & HD Cohort Controls** 12](#_Toc31979507)

[**Graphs:** 13](#_Toc31979508)

## **Cerebellum: PDD v AD & HD Cohort Controls**

Auckland AD and HD controls rejected for inclusion due to clear separation on both PCA and PLS-DA plots.

**PCA Plots:**


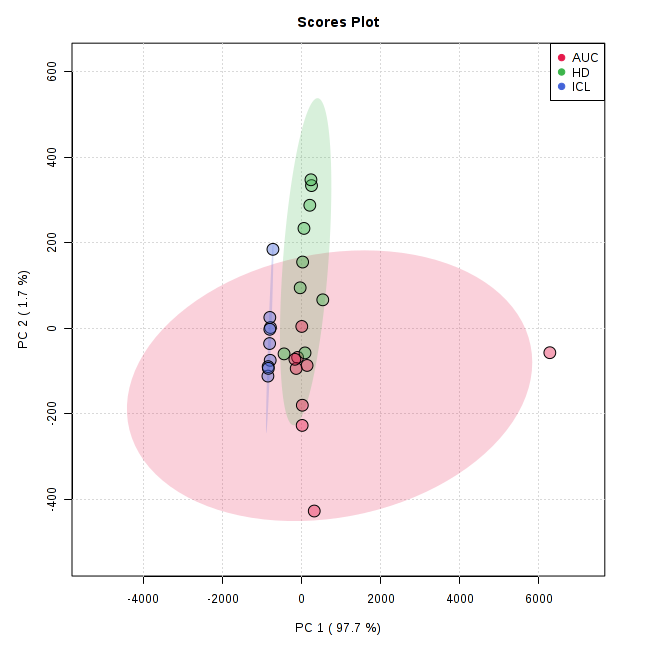

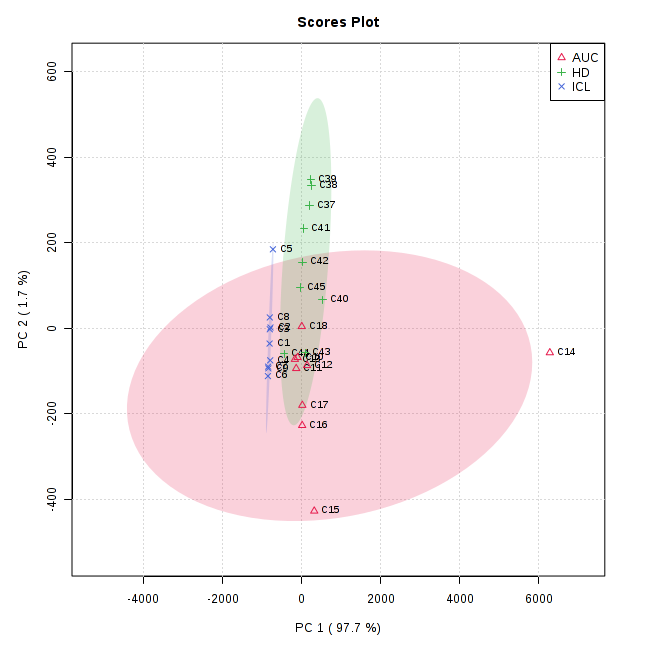


**PLS-DA Plots:**


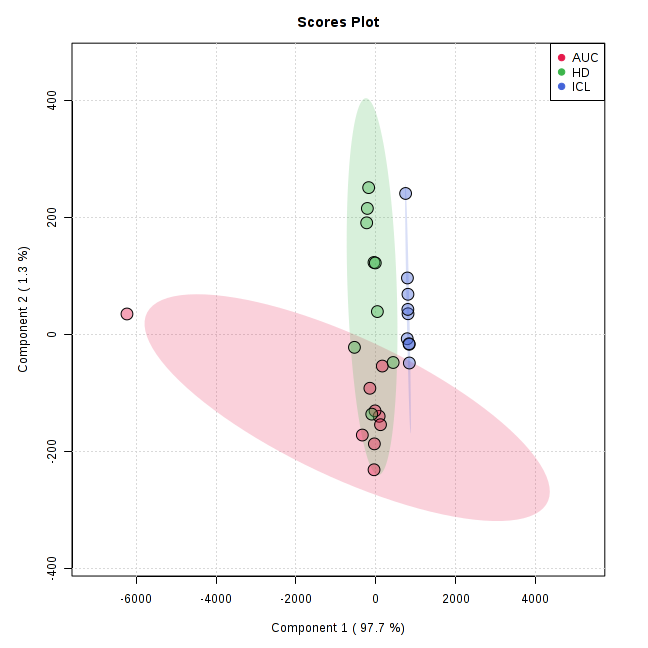

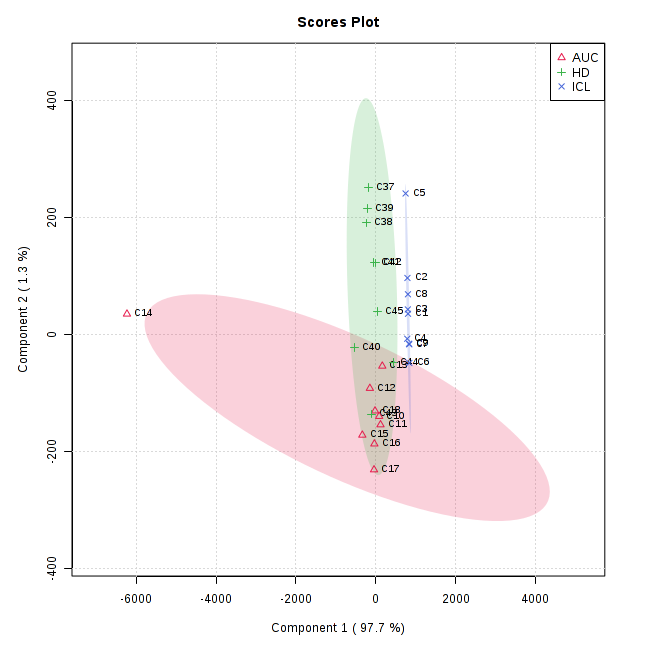


## **Graphs:**

Inter-cohort differences determined by Kruskal-Wallis test followed by Dunn’s post-hoc multiple comparisons test. ICL = Imperial college London; HD = Huntington’s disease; * p < 0.05; ** p < 0.01; *** p < 0.001; **** p < 0.0001

## **Cingulate Gyrus: PDD v AD Cohort Controls**

AD controls from Manchester and Newcastle cohorts accepted for inclusion due to no separation on either PCA or PLS-DA plots. Auckland samples rejected due to separation from PDD controls.

**PCA Plots:**


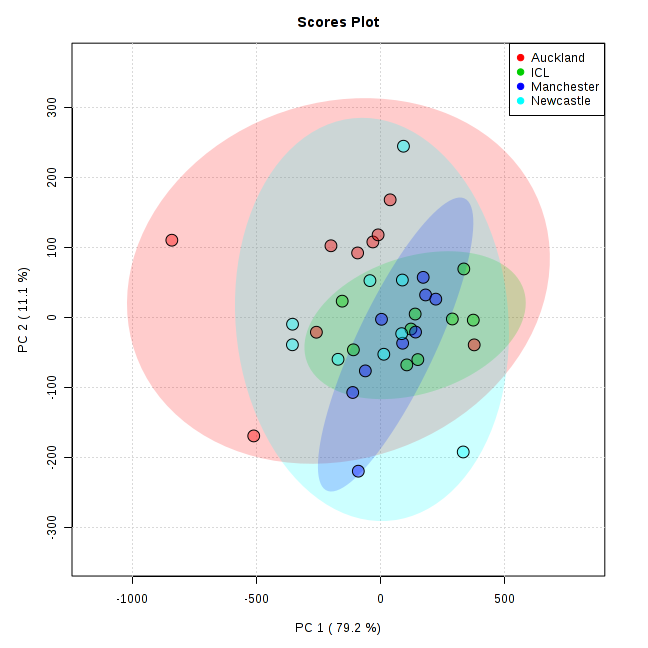

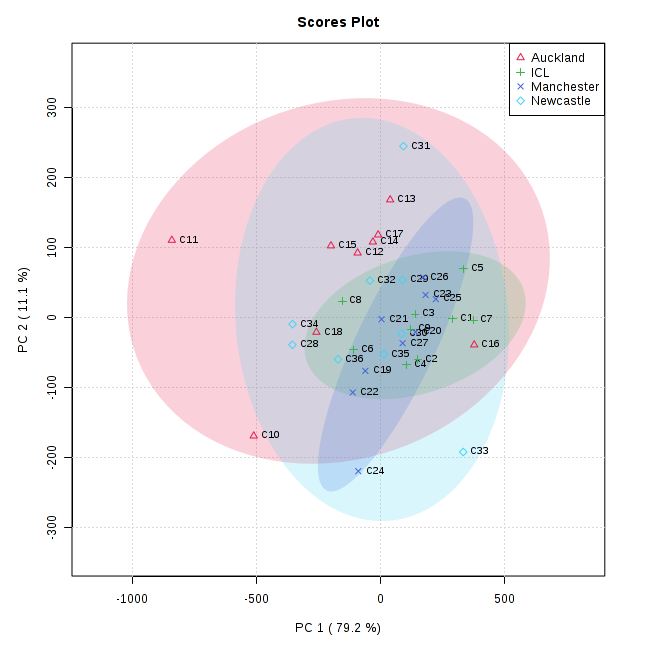


**PLS-DA Plots:**


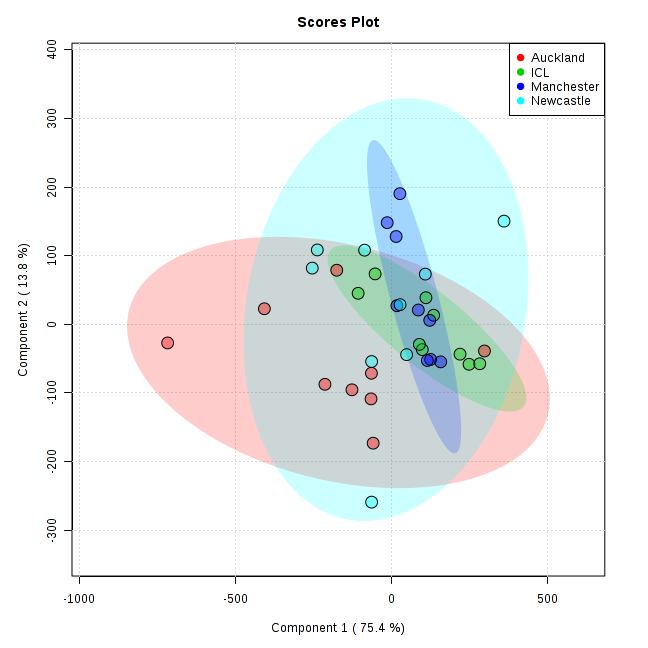

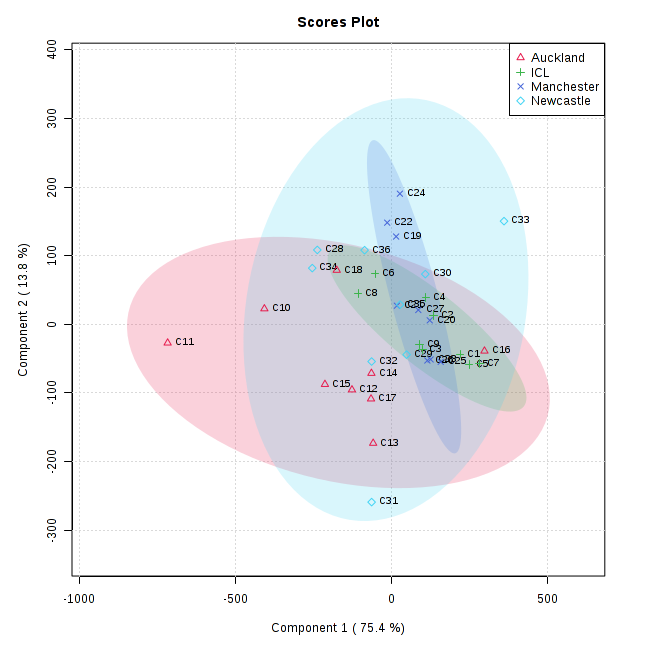


## **Graphs:**

Inter-cohort differences determined by Kruskal-Wallis test followed by Dunn’s post-hoc multiple comparisons test. ICL = Imperial college London; * p < 0.05; ** p < 0.01; *** p < 0.00

## **Hippocampus: PDD v AD & HD Cohort Controls**

AD controls from Auckland and HD controls were accepted for inclusion due to no separation on either PCA or PLS-DA plots.

**PCA Plots:**


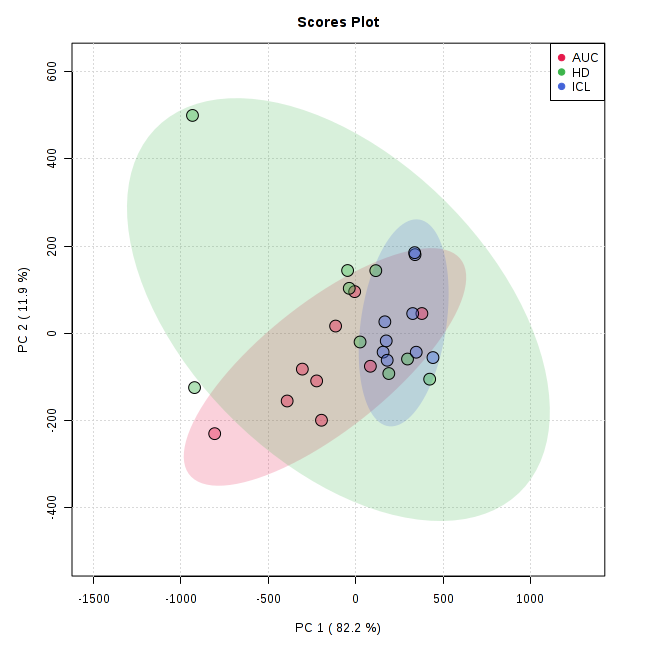

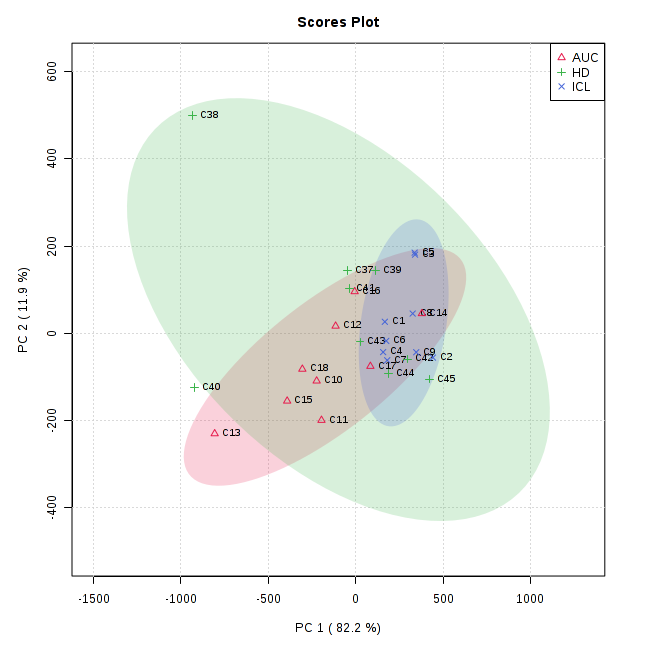


**PLS-DA Plots:**


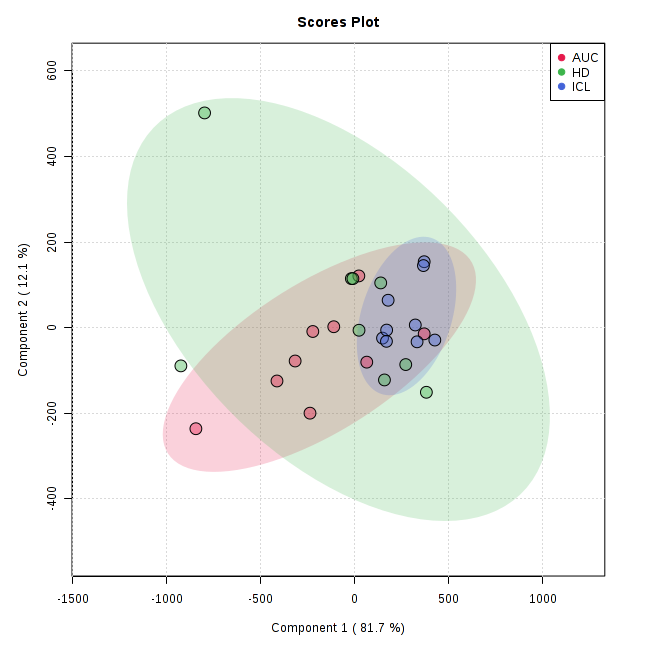

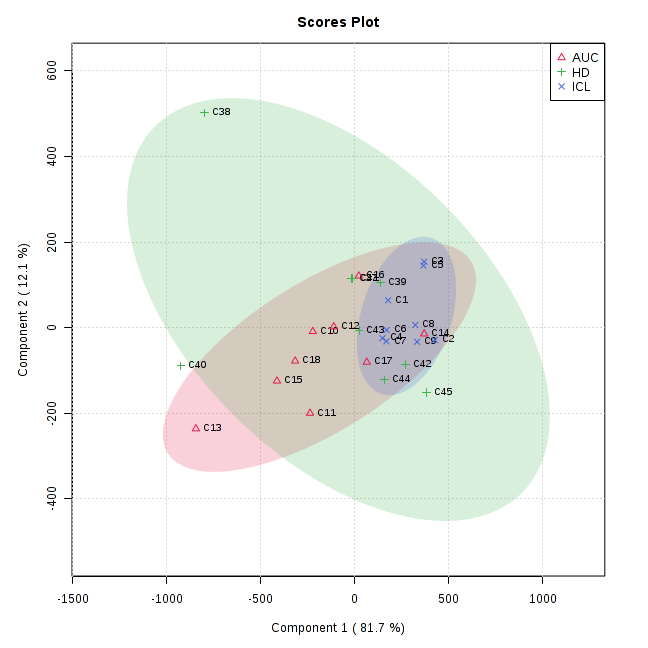


## **Graphs:**

Inter-cohort differences determined by Kruskal-Wallis test followed by Dunn’s post-hoc multiple comparisons test. ICL = Imperial college London; HD = Huntington’s disease; ** p < 0.01

## **Middle Temporal Cortex: PDD v AD & HD Cohort Controls**

AD controls from Auckland and HD controls were accepted for inclusion due to no separation on either PCA or PLS-DA plots.

**PCA Plots:**


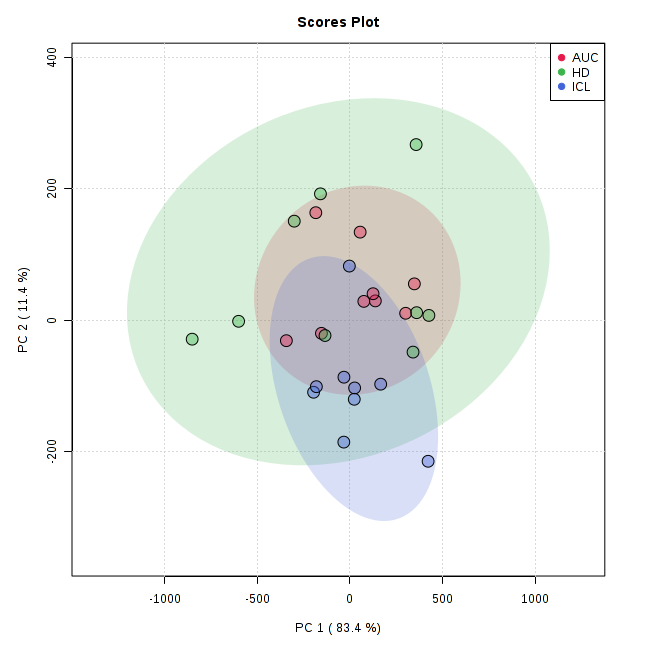

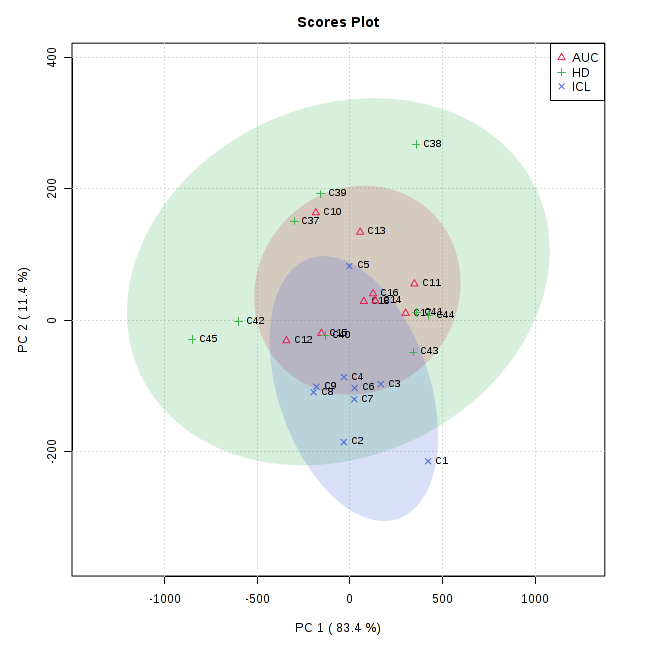


**PLS-DA Plots:**


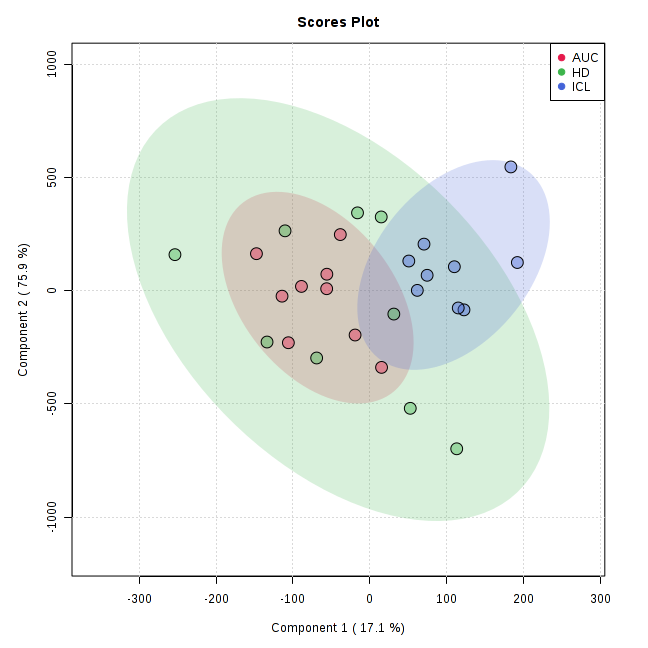

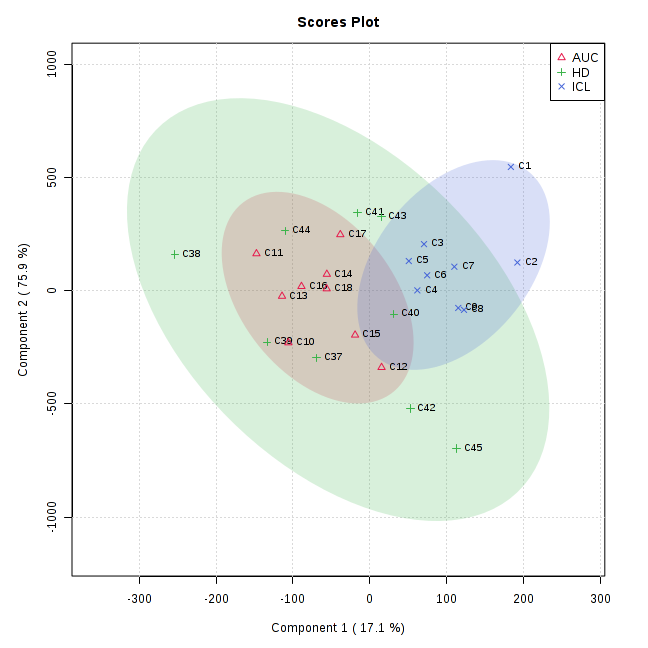


## **Graphs:**

Inter-cohort differences determined by Kruskal-Wallis test followed by Dunn’s post-hoc multiple comparisons test. ICL = Imperial college London; HD = Huntington’s disease; * p < 0.05; ** p < 0.01

## **Substantia Nigra: PDD v HD Cohort Controls**

HD controls were rejected for inclusion due to clear separation on both PCA and PLS-DA plots.

**PCA Plots:**


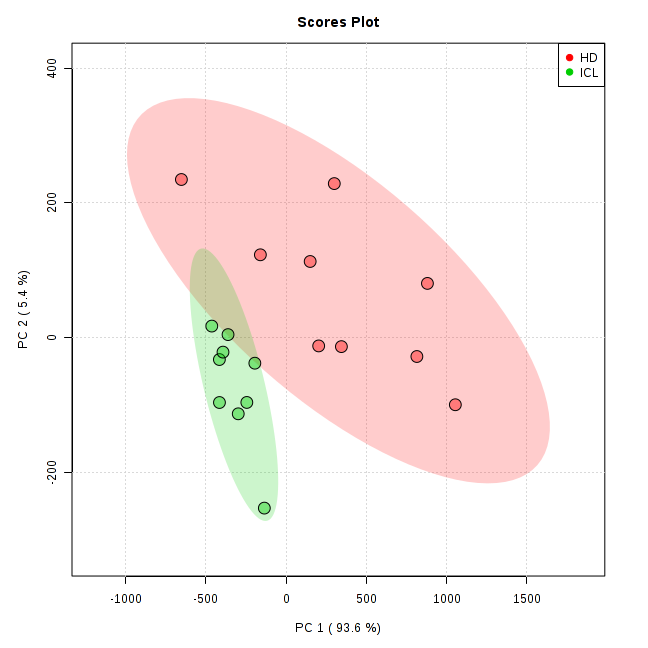

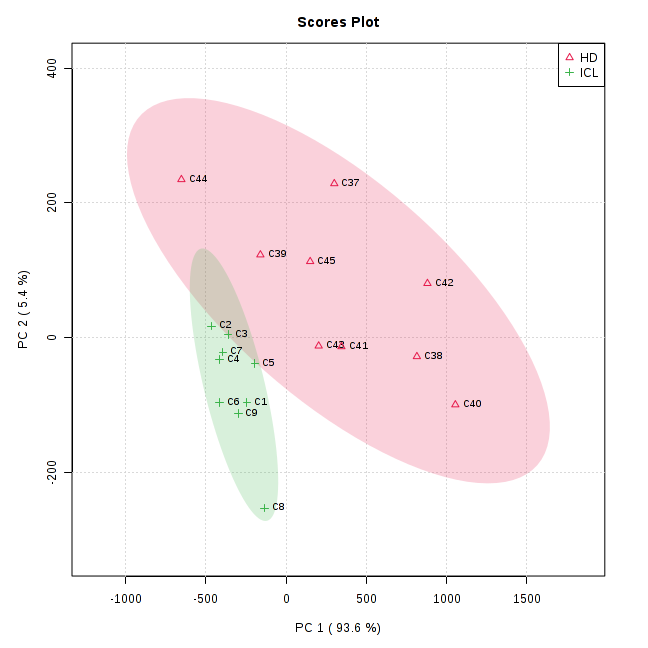


**OPLS-DA Plots:**


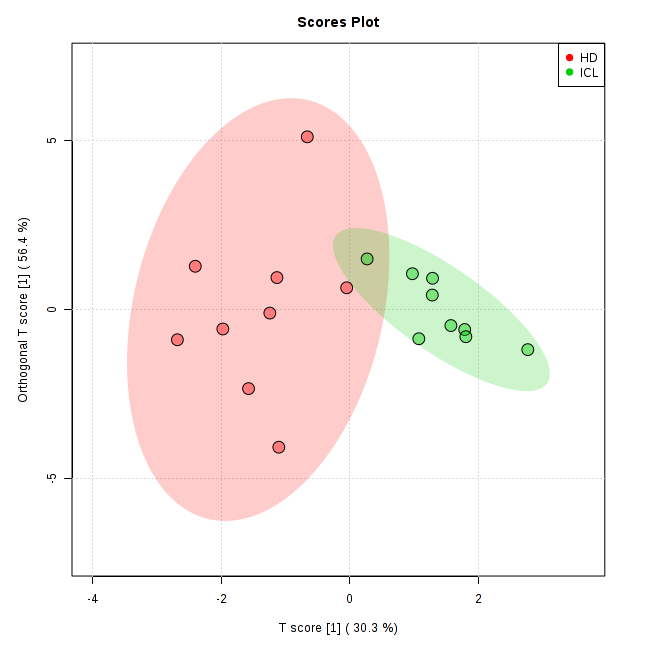

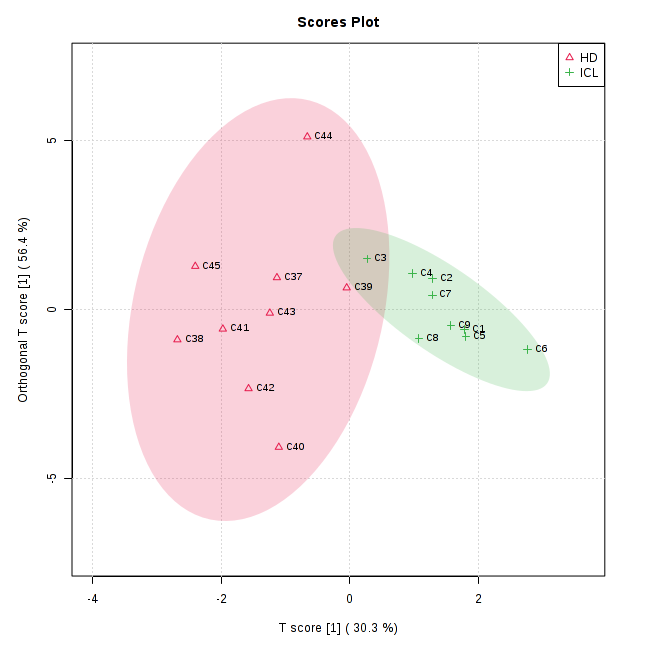


## **Graphs:**

Inter-cohort differences determined by Kruskal-Wallis test followed by Mann-Whitney U test. ICL = Imperial college London; HD = Huntington’s disease; ** p < 0.01

## **Motor Cortex: PDD v AD & HD Cohort Controls**

AD controls from Auckland and HD controls were accepted for inclusion due to no separation on either PCA or PLS-DA plots.

**PCA Plots:**


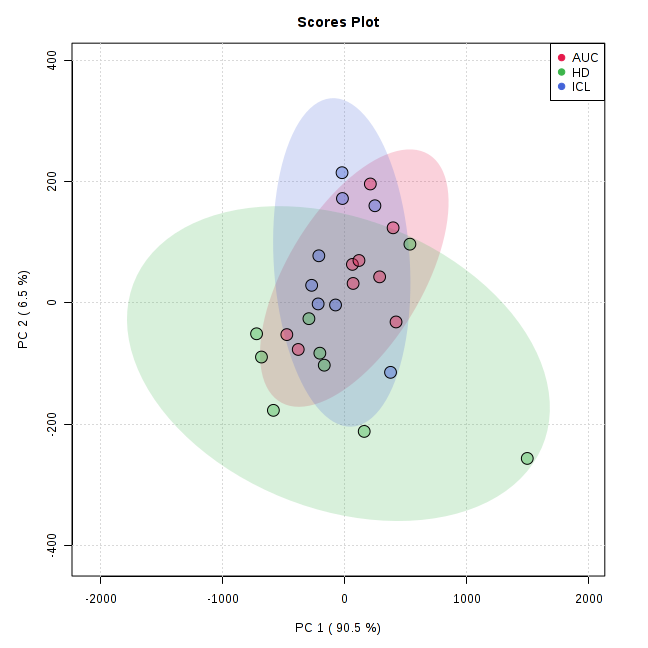

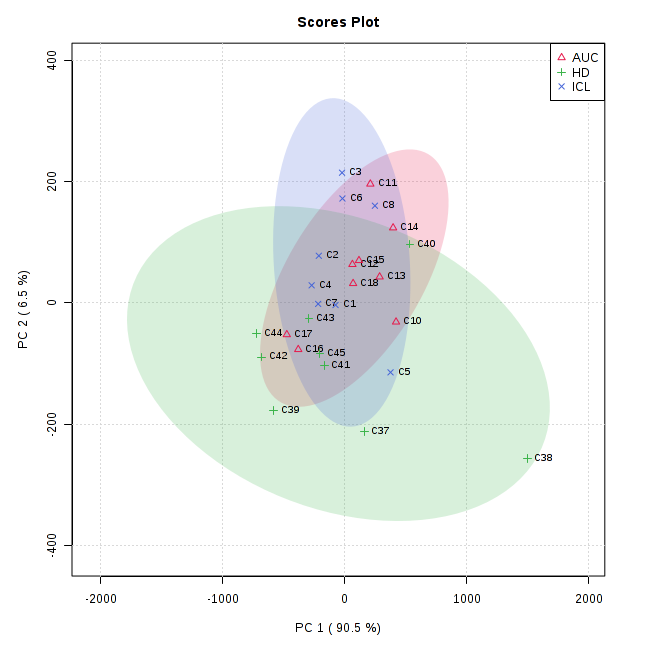


**PLS-DA Plots:**


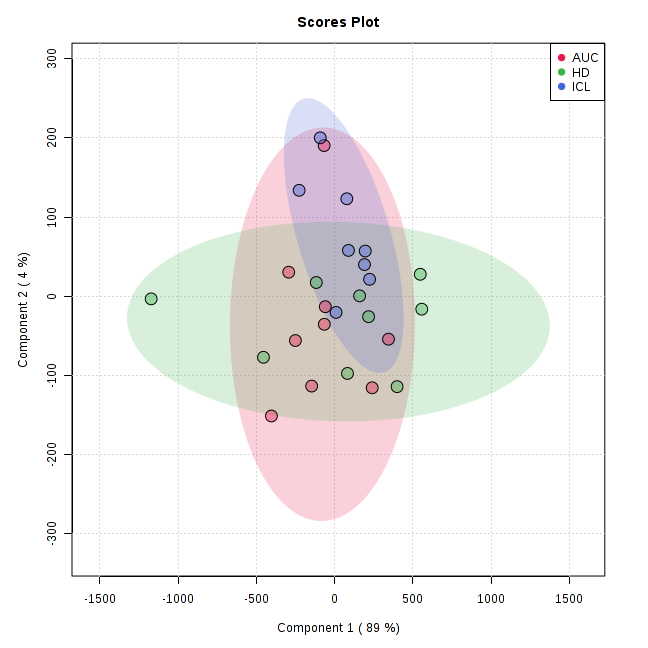

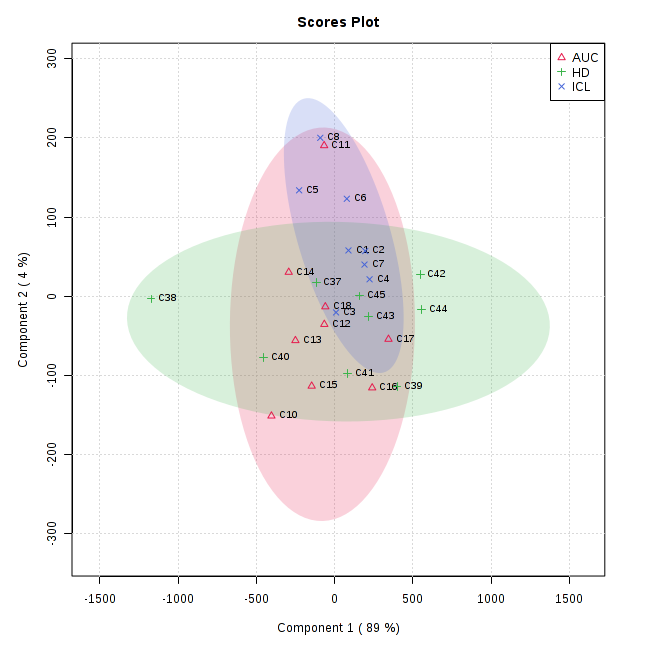


## **Graphs:**

Inter-cohort differences determined by Kruskal-Wallis test followed by Dunn’s post-hoc multiple comparisons test. ICL = Imperial college London; HD = Huntington’s disease; * p < 0.05; ** p < 0.01
