# Supplementary material for: Widespread Decreases in Cerebral Copper Are Common to Parkinson's Disease Dementia and Alzheimer's Disease Dementia
Source: Front Aging Neurosci. 2021 Mar 3;13:641222. doi: 10.3389/fnagi.2021.641222 (PMC7966713; doi:10.3389/fnagi.2021.641222)
Supplement: Supplementary file 1 [file Data_Sheet_1.zip › PDD Paper - Supplementary Material D (Statistical Power).docx]

Supplementary Material D

These tables show tests of statistical power for metal concentrations in individual regions of dry-weight brain tissue. Values highlighted in bold satisfy statistical power minimum requirement of 80% or sample size required of ≤ 18. Values were determined using the DSS Statistical Power and Sample Size Calculators. Link: https://www.dssresearch.com/resources/calculators/statistical-power-calculator-average/

**Supplementary Table 1: Statistical Power in Substantia Nigra (SN)**

| Metal | Statistical Power (p < 0.05) | Sample Size Required (p < 0.05) |
| --- | --- | --- |
| Na | 5.7 | 386 |
| Mg | 11.1 | 47 |
| K | 6.2 | 241 |
| Ca | 10.3 | 55 |
| Mn | 59.1 | **5** |
| Fe | 5.8 | 373 |
| Cu | **96.3** | **2** |
| Zn | 40.2 | **8** |
| Se | 20.4 | 19 |

**Supplementary Table 2: Statistical Power in Cingulate Gyrus (CG)**

| Metal | Statistical Power (p < 0.05) | Sample Size Required (p < 0.05) |
| --- | --- | --- |
| Na | 16.0 | 52 |
| Mg | 14.2 | 64 |
| K | 40.2 | 20 |
| Ca | 43.5 | **16** |
| Mn | 60.8 | **7** |
| Fe | 12.9 | 57 |
| Cu | **85.1** | **6** |
| Zn | 46.5 | **13** |
| Se | 28.6 | 24 |

**Supplementary Table 3: Statistical Power in Hippocampus (HP)**

| Metal | Statistical Power (p < 0.05) | Sample Size Required (p < 0.05) | |
| --- | --- | --- | --- |
| Na | 25.2 | | 23 |
| Mg | 39.3 | | 22 |
| K | 70.5 | | **9** |
| Ca | 18.1 | | 38 |
| Mn | 65.3 | | **11** |
| Fe | 6.3 | | 440 |
| Cu | **98.6** | | **4** |
| Zn | **96.1** | | **4** |
| Se | **85.9** | | **7** |

**Supplementary Table 4: Statistical Power in Locus Coeruleus (LC)**

| Metal | Statistical Power (p < 0.05) | Sample Size Required (p < 0.05) |
| --- | --- | --- |
| Na | 18.5 | 22 |
| Mg | 26.4 | **14** |
| K | 13.7 | 34 |
| Ca | 40.2 | **8** |
| Mn | 63.1 | **5** |
| Fe | 11.6 | 43 |
| Cu | 21.1 | **18** |
| Zn | 5.8 | 344 |
| Se | 5.7 | 406 |

**Supplementary Table 5: Statistical Power in Medulla (MED)**

| Metal | Statistical Power (p < 0.05) | Sample Size Required (p < 0.05) |
| --- | --- | --- |
| Na | 11.4 | 45 |
| Mg | 72.5 | **4** |
| K | 45.3 | **7** |
| Ca | 60.8 | **5** |
| Mn | **91.4** | **2** |
| Fe | 22.1 | **17** |
| Cu | 55.6 | **6** |
| Zn | 36.0 | **10** |
| Se | 9.4 | 65 |

**Supplementary Table 6: Statistical Power in Primary Visual Cortex (PVC)**

| Metal | Statistical Power (p < 0.05) | Sample Size Required (p < 0.05) |
| --- | --- | --- |
| Na | 5.7 | 388 |
| Mg | 35.4 | 10 |
| K | 9.4 | 65 |
| Ca | 33.2 | 10 |
| Mn | 47.4 | **7** |
| Fe | 16.0 | 26 |
| Cu | 62.4 | **5** |
| Zn | 6.4 | 206 |
| Se | 5.0 | 88336 |

**Supplementary Table 7: Statistical Power in Middle Temporal Gyrus (MTG)**

| Metal | Statistical Power (p < 0.05) | Sample Size Required (p < 0.05) |
| --- | --- | --- |
| Na | 34.3 | **14** |
| Mg | 13.8 | 75 |
| K | **83.2** | **7** |
| Ca | 44.6 | 21 |
| Mn | **91.9** | **5** |
| Fe | 13.0 | 71 |
| Cu | **96.1** | **4** |
| Zn | 9.0 | 119 |
| Se | 77.9 | **9** |

**Supplementary Table 8: Statistical Power in Cerebellum (CB)**

| Metal | Statistical Power (p < 0.05) | Sample Size Required (p < 0.05) |
| --- | --- | --- |
| Na | 5.9 | 313 |
| Mg | 7.9 | 94 |
| K | 5.9 | 311 |
| Ca | 7.3 | 118 |
| Mn | 6.8 | 148 |
| Fe | 7.8 | 94 |
| Cu | 5.0 | 7437 |
| Zn | 7.7 | 100 |
| Se | 29.8 | **11** |

**Supplementary Table 9: Statistical Power in Motor Cortex (MCX)**

| Metal | Statistical Power (p < 0.05) | Sample Size Required (p < 0.05) |
| --- | --- | --- |
| Na | 24.5 | 25 |
| Mg | **87.3** | **7** |
| K | **81.3** | **7** |
| Ca | 6.6 | 220 |
| Mn | **96.0** | **4** |
| Fe | 58.5 | **11** |
| Cu | **97.1** | **4** |
| Zn | 73.5 | **7** |
| Se | **91.4** | **6** |
